# Synthesis of covalently linked knotted cage frameworks

**DOI:** 10.1038/s44160-025-00822-7

**Published:** 2025-06-25

**Authors:** Yuchong Yang, Tanya K. Ronson, Paula C. P. Teeuwen, Simone Zucchelli, Andrew W. Heard, Paola Posocco, David J. Wales, Jonathan R. Nitschke

**Affiliations:** 1 Yusuf Hamied Department of Chemistry, University of Cambridge, Cambridge, UK; 2 Department of Engineering and Architecture, University of Trieste, Trieste, Italy

**Keywords:** Interlocked molecules, Molecular capsules

## Abstract

Interwoven molecular structures underpin the functions of many biomolecules, yet synthesizing artificial topologically complex structures in high yield remains challenging. Here we describe a streamlined, high-yield one-pot synthesis of knotted cage frameworks by using a subcomponent designed to bridge over the faces of a predesigned cage framework. A Zn^II^_4_L_3_ (where L corresponds to a tritopic pyridyl-imine ligand that coordinates to the metal vertices) open-faced cage framework was employed as the basis for a topologically chiral perplexane, and a Zn^II^_4_L_4_ tetrahedron was built into a topologically chiral trefoil tetrahedron. Both interwoven architectures can be prepared through one-pot subcomponent self-assembly from a trialdehyde, the bridging triamine and a zinc(II) salt. The trefoil tetrahedron was observed to mechanically lock guests inside the cavity, resulting in a guest exchange half-life 17,000 times longer than that of the original tetrahedral cage. Both cage frameworks were reduced and demetallated to yield metal-free interwoven structures, with the perplexane producing an achiral product and the trefoil tetrahedron maintaining its topological chirality. Our strategy may enable the knotting of many existing cage frameworks produced using subcomponent self assembly, enhancing their robustness and ability to lock guests inside.

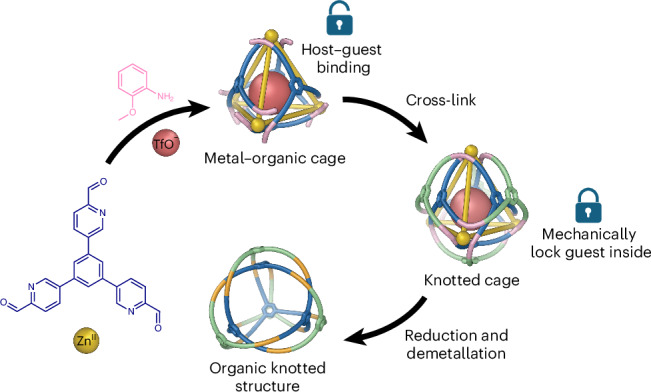

## Abstract (web summary)

Synthesizing topologically complex interwoven molecules with high yield remains challenging due to structural preorganization demands. Here face-bridging ligands on metal–organic cage frameworks are shown to enable high-yield synthesis of knotted cages that mechanically trap guests inside, enhancing guest retention and structural robustness.

## Main

Weaving has enabled human progress for millennia, from baskets and textiles^1^, to the interlaced structures of the latest synthetic molecular machines^2^. Complex topologies underpin the functions of natural biomolecular structures, including proteins^3^, DNA^4^ and RNA^5^. Inspired by these natural systems, the Sauvage group first used metal templation to obtain macroscopic quantities of a trefoil knot^6^. Subsequently, a range of artificial interwoven structures with more complex topologies, such as catenanes^7–10^, molecular knots^11^ and Borromean rings^12–14^, have been developed through various strategies, including selective linking of the ends of interwoven grids^15^, hydrophobic effects^16^ and metal ion templation^17–20^. These topologically complex assemblies have potential in applications such as ion transport^21^, catalysis^22^ and tailoring polymer properties^23^. While key design principles for such interwoven structures are now established, the development of higher order, topologically complex architectures with bifurcated strands remains a challenge^17^. This difficulty arises primarily from the requirement for the preorganization of a stable framework, which must then be linked together in the precise way needed to give the desired topology, while avoiding linkages that produce different product structures or mixtures.

Metal-coordination-driven self-assembly has emerged as a robust approach for synthesizing polyhedral metal–organic cages, encompassing structures such as Platonic and Archimedean solids^24–28^, along with prisms and antiprisms^29,30^. These polyhedral cages, with precise geometric configurations and enclosed internal cavities, are useful in applications including natural product encapsulation^29^, selective molecular separation and delivery^31^, sensing^32^, catalysis^33^ and the stabilization of reactive species^34^.

**Fig. 1:**
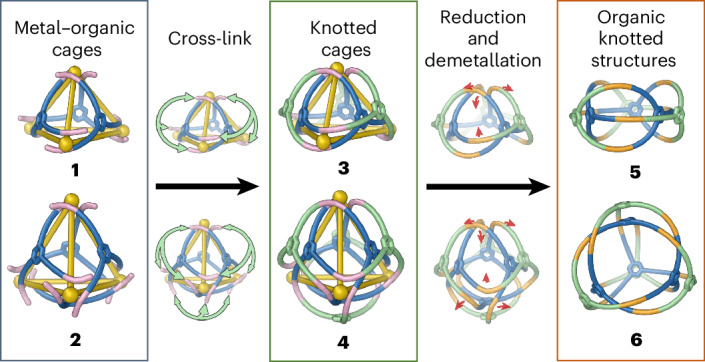
Exterior cross-linking strategy for the construction of knotted cages and organic covalent-interwoven structures. Cage frameworks **1** and **2** serve as scaffolds for cross-linking into knotted cages **3** and **4** through the addition of tritopic linkers. The dynamic-covalent imine bonds of these topologically complex structures may then be reduced to secondary amines, and the metal ions removed, to create metal-free organic perplexane **5** and knotted tetrahedron **6**. The red arrows indicate how the organic backbone structure moves during structural transformations.

We hypothesized that an exterior cross-linking strategy, utilizing a well-defined metal–organic cage as the structural core, could promote the formation of interwoven structures with complex topologies, as shown in Fig. 1. This approach aims to streamline the synthesis while simultaneously enhancing the complexity of the interwoven structure and overall yield of the process. Such a cross-linking strategy could also increase robustness through a greater density of connections between subunits, and hinder guest release via a more rigid and tangled framework for the escaping guest to navigate.

In this Article, we report a high yield, one-pot preparation of interwoven three-dimensional structures **3** and **4** based on open-faced tetrahedron **1** and enclosed tetrahedron **2**, respectively. Both **2** and **4** were observed to bind anionic guests, but guest exchange for interwoven **4** is 17,000 times slower than for the non-interwoven congener **2**. The added layer of cross-linked molecular strands rigidifies the cage and presents a mechanical barrier to guest exit, thereby locking the guests within the cavity. This stronger guest retention could be useful in the context of delivery, where slow and controlled rates of release are necessary.

Both open-faced **3** and fully enclosed **4** were reduced and demetallated to yield fully organic, covalently linked interwoven structures **5** and **6**, respectively. Both **4** and **6** are chiral, with the topological chirality^35^ of **6** being determined by the handedness of the chiral tetrahedral (*T* point group) framework of its precursor **4**. Although the framework of **3** has a handedness determined by the stereochemistry of its constituent metal centres, metal-free **5** is topologically achiral, as noted by Tilley and co-workers^36^, who referred to this topology as a perplexane. As shown in the upper right of Fig. 1, the branches of **5** can adopt an achiral *C*_3h_-symmetric configuration with a central mirror plane. Our method thus allows access to topologically chiral and achiral products from the same set of precursors.

## Results and discussion

**Fig. 2:**
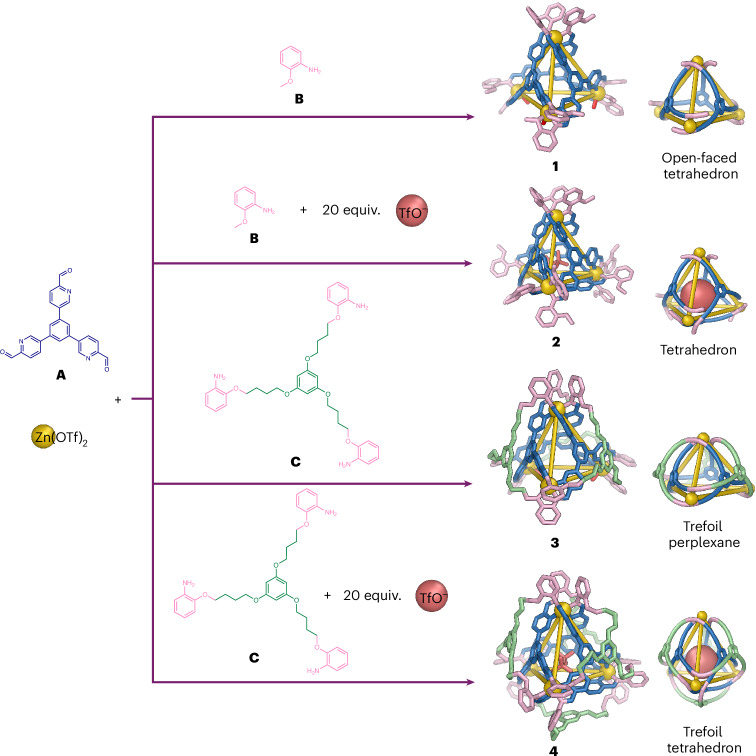
Subcomponent self-assembly of tetrahedral structures 1, 2, 3 and 4. Trialdehyde **A** reacted with *o*-anisidine **B** and zinc(II) triflate to generate open-faced **1**, or enclosed **2** in the presence of excess triflate. Tritopic triamine **C** bridged over the tetrahedron faces formed from **A** residues, producing interwoven open-faced **3** and fully enclosed **4**, again in the presence of excess triflate.

As shown in Fig. 2, trialdehyde **A** (3 equiv.) and zinc(II) trifluoromethanesulfonate (triflate or TfO^–^, 4 equiv.) reacted with *o-*anisidine **B** (9 equiv.) or triamine **C** (3 equiv.) in acetonitrile to produce open-faced assemblies **1** and **3**, respectively. The solution-phase structure of **1** was confirmed through nuclear magnetic resonance (NMR) spectroscopy and electrospray ionization mass spectrometry (ESI-MS), which both gave results consistent with an open-faced Zn^II^_4_L_3_ tetrahedron, as shown in Supplementary Figs. 3–11. L corresponds to the tritopic pyridyl-imine ligand formed from imine condensation of subcomponent **A** with **B**. Analysis of the ^1^H NMR and heteronuclear single quantum coherence (HSQC) spectra (Supplementary Figs. 3 and 8) showed three distinct sets of resonances, consistent with a *C*_3_-symmetric framework. ^1^H NMR diffusion-ordered spectroscopy (DOSY) indicated the presence of a single species with a hydrodynamic radius of 12.8 Å (Supplementary Fig. 9). This radius is consistent with the GFN-FF^37^ minimized structure of **1**, based on the crystal structure of **3** (Fig. 2). All four Zn^II^ centres have the same handedness, with Zn^II…^Zn^II^ distances ranging from 11.4 to 12.2 Å. The observed stoichiometry incorporates one tris(pyridylimine)-chelated Zn^II^ vertex and three bis(pyridylimine) Zn^II^ vertices. Thus, only three of the four faces are covered by ligands, forming an open-faced tetrahedron. A similar open-faced geometry was reported by Shionoya and co-workers^38^, attributed to the coordinative flexibility of Zn^II^.

Tritopic subcomponent **C** joins three anisidine residues covalently in a configuration that we hypothesized would bridge over the faces of a tetrahedral framework, thus resulting in a topologically complex interwoven structure. The formation of open-faced interwoven tetrahedron **3** (Fig. 2) confirmed this hypothesis. The structure of **3** was confirmed by NMR spectroscopy and ESI-MS, as shown in Supplementary Figs. 22–31. DOSY ^1^H NMR (Supplementary Fig. 29) gave a solvodynamic radius of 12.8 Å for **3**. This radius was consistent with the size of **3** obtained in the solid state, as determined by single-crystal X-ray diffraction (XRD) at the Diamond Light Source synchrotron.

**Fig. 3:**
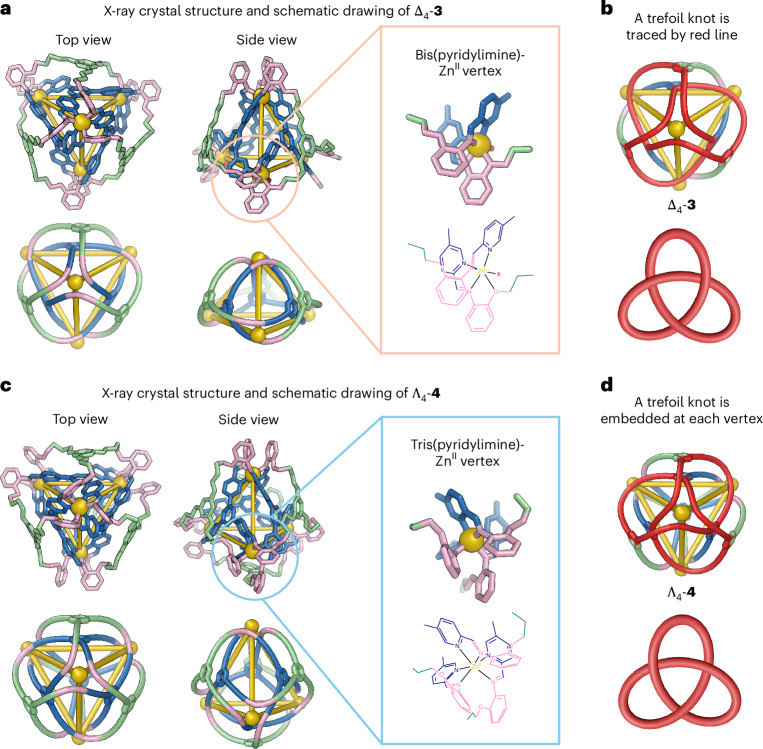
X-ray crystal structures of 3 and 4. Top and side views of both structures are shown above schematic views illustrating their topology. Disorder, hydrogen atoms, counterions and solvent molecules of crystallization are omitted for clarity. **a**,**b**, The structure of **3** (**a**) contains three bis(pyridylimine) vertices with an additional counterion (shown as a red ‘X’ in the expansion) bound to zinc, and one tris(pyridylimine) apical vertex, lending it non-crystallographic *C*_3_ point group symmetry. The shortest closed path along the ligand (shown in red) describes a trefoil knot (**b**). **c**,**d**, Structure **4** (**c**) has idealised chiral tetrahedral (*T*) point group symmetry, with all tris(pyridylimine)zinc vertices coordinatively saturated. Trefoil knot motifs are similarly embedded at each vertex of **4** (**d**).

Complex **3** exhibits *C*_3_ point group symmetry, as reflected in its NMR spectrum (Supplementary Fig. 27). From the top view of **3** (Fig. 3b), a trefoil knot can be traced as the shortest path through the cage framework, as illustrated by the red line in Fig. 3b, thus forming a trefoil perplexane^36^ structure or a branched trefoil knot framework. Compound **3** therefore possesses a continuous graph, with a single bifurcating molecular strand woven around the four templating Zn^II^ centres.

The Zn^II…^Zn^II^ distances between adjacent metal centres range from 10.6 to 11.4 Å, as shown in Fig. 3a. As with **1,** **3** incorporates one tris(pyridylimine)-chelated Zn^II^ vertex and three bis(pyridylimine)-chelated Zn^II^ vertices. This results in one open face (Fig. 2) and reflects the underlying cage framework shared with **1**. All four Zn^II^ vertices within **3** have the same handedness, with both Δ_4_ and Λ_4_ enantiomers related by inversion in the crystal.

Metal–organic cage receptors can reconfigure to bind guests^39,40^. We therefore explored the addition of excess TfO^−^ (20 equiv.) as a template during cage synthesis. In place of **1** or **3**, this triflate was found to drive the formation of coordinatively saturated structure **2** or **4**, as shown in Fig. 2, by binding within the cage cavity.

The tetrahedral structure of **2** was confirmed by NMR spectroscopy and ESI-MS, as shown in Supplementary Figs. 12–21. DOSY ^1^H NMR analysis of **2** showed a hydrodynamic radius of 12.5 Å (Supplementary Fig. 19), consistent with a tetrahedral framework. The ^19^F NMR spectrum of **2** exhibited signals corresponding to both free and encapsulated TfO^−^ (Supplementary Fig. 14), consistent with TfO^−^ templating the formation of the framework of **2** by binding inside its cavity. We infer that all faces of **2** are covered by ligands, similar to a previously reported tetrahedral cage^41^ prepared from **A**, as shown in Fig. 2. We infer the enclosed cavity of **2** serves as a more favourable host for triflate than the open bowl-shaped cavity of **1**. All data are consistent with *T* point symmetry for **2**, with all metal centres of a single handedness within each cage (Supplementary Figs. 12 and 17).

Using a similar strategy, knotted tetrahedron **4** was constructed through the assembly of zinc(II) with tritopic subcomponents **A** and **C** in the presence of excess triflate. This approach yielded a topologically intricate structure where the tetrahedral core of **2** was cross-linked by **C** residues. NMR spectroscopy and ESI-MS provided results consistent with the presence of an interwoven tetrahedral framework, as shown in Supplementary Figs. 32–41. Only one environment per ligand proton of **4** was observed in the ^1^H NMR spectrum, as with **2**. DOSY NMR gave a solvodynamic radius of 12.8 Å (Supplementary Fig. 39), consistent with the crystal structure of **4**. An alternative preparation of **4** was also explored, whereby subcomponent **C** was added to tetrahedron **2**. This reaction did produce some **4**, but the NMR spectrum of the product (Supplementary Fig. 42) showed substantial side-product formation as well, potentially stemming from mismatches or cross-linking of **C** residues between cages.

Single-crystal X-ray diffraction at the Diamond Light Source confirmed the structure of **4**, as shown in Fig. 3c, containing four tris-pyridyl(imine)-chelated Zn^II^ centres bridged by a continuous double-layered woven ligand. The inner core of the structure is analogous to previously reported M_4_L_4_ cages prepared from **A** (where M refers to an octahedral metal cation), with the outer ligand linking each of the three adjacent faces, resulting in the formation of a single knotted ligand wrapping around the four zinc vertices. Looking down the *C*_3_ axis of each vertex, a trefoil knot can be traced as the shortest circular path along the ligand, as illustrated by the red line in Fig. 3d. The Zn^II…^Zn^II^ distances between adjacent Zn^II^ centres range from 11.4 to 11.6 Å. All vertices again have the same Δ or Λ handedness, with *T* point group symmetry. Although **4** was observed to crystallize in the chiral space group *P*2_1_2_1_2_1_ as a racemic twin, we infer that in the bulk **4** exists as a racemic mixture of two topologically chiral enantiomers (Fig. 3d). The crystal structure also shows a TfO^−^ anion is encapsulated within the cavity of **4** (Supplementary Fig. 92), consistent with the data obtained from the ^19^F NMR spectrum (Supplementary Fig. 34), which is also consistent with TfO^−^ adopting the role of template during the synthesis of **4**.

Guest binding affinity is critical in determining the functional utility of host molecules^42–44^. However, the stable encapsulation of guests with weak binding affinities remains a challenge in host–guest chemistry. We hypothesized that the interwoven framework of **4** might rigidify the cage and present a mechanical barrier to guest exit, thus offering an effective strategy to enhance the stabilization of bound guests.

The guest exchange behaviour of knotted cage **4** was investigated, with its congener **2** serving as a control. Previous studies^41^ have shown that ReO_4_^−^ binds with approximately 100-fold greater affinity than TfO^−^ within tetrahedral cages sharing the same framework as **2** and **4**. To probe guest exchange, excess ReO_4_^−^ was added to solutions of TfO^−^⊂**2** and TfO^−^⊂**4**, and guest exchange was monitored using ^1^H NMR spectroscopy. New ^1^H NMR peaks corresponding to the perrhenate adducts were observed and the ^19^F NMR peaks corresponding to encapsulated triflate disappeared during guest exchange (Supplementary Figs. 43–52).

**Fig. 4:**
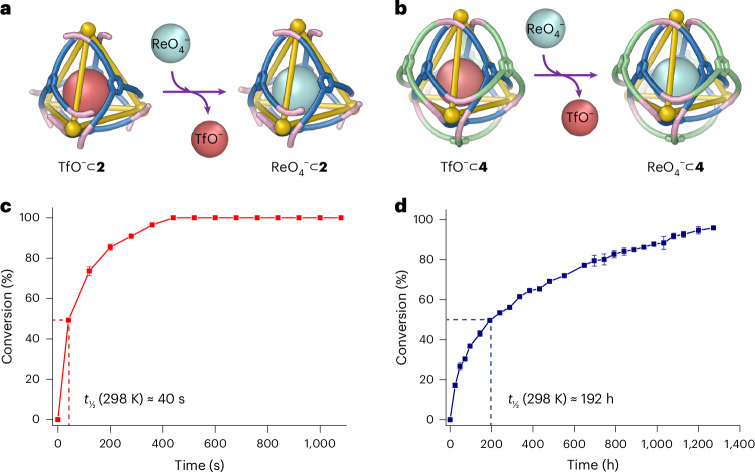
Comparison of anionic guest exchange between 2 and 4. **a**–**d**, Schematic illustrations of the displacement of TfO^−^ by ReO_4_^−^ within **2** (**a**) and **4** (**b**). **c** and **d** show the traces of these processes within **2** and **4**, respectively, as monitored by ^1^H NMR. The conversion data in panels **c** and **d** are presented as mean ± standard deviation, with each point calculated from six individual NMR signals (three pairs). See Supplementary Section 4 for details. The dashed lines in **c** and **d** indicate the time corresponding to the half-life of conversion. Source data

Different guest displacement rates were observed for TfO^−^⊂**2** and TfO^−^⊂**4** upon the addition of ReO_4_^−^ in the presence of a total of 28 equiv. TfO^−^. As shown in Fig. 4, the addition of ReO_4_^−^ to a solution of TfO^−^⊂**2** resulted in a rapid increase in the ^1^H NMR signals corresponding to ReO_4_^−^⊂**2**, while the signals for TfO^−^⊂**2** diminished concurrently. By contrast, the ^1^H NMR signals for ReO_4_^−^⊂**4** increased more slowly under identical conditions. The half life (*t*_1/2_) for conversion of TfO^−^⊂**2** into ReO_4_^−^⊂**2** was measured to be 40 s, whereas the TfO^−^⊂**4** to ReO_4_^−^⊂**4** conversion required 192 hours to reach the same conversion level under identical conditions. The knotting of the cage framework of **4** thus reduced the rate of anionic guest exchange by a factor of 17,000.

To quantify how the woven structure of **4** mechanically restricts the dynamic motion of its tetrahedral core during guest exchange, the enthalpy (Δ*H*^‡^) and entropy (Δ*S*^‡^) of activation for the substitution of perrhenate for triflate within **2** and **4** were analysed using the Eyring equation (Supplementary Figs. 54–57). The enthalpy of activation for **2** (49.5 ± 2.4 kJ mol^-1^) was less than for **4** (65.1 ± 2.4 kJ mol^-1^), consistent with a more rigid host framework for **4**, with a higher barrier to deformation. The entropy of activation is likewise less favourable for **4** (−141.0 ± 9.9 J mol^-1^ K^-1^) than for **2** (−113.1 ± 11.5 J mol^-1^ K^-1^), consistent with a more rigid and tangled framework for the escaping guest to navigate within **4**. Arrhenius analysis (Supplementary Fig. 58) further confirmed a higher activation energy and less favourable pre-exponential factor in the case of **4**.

Molecular dynamics simulations were conducted using a CHARMM general force field^45^ to analyse the dynamics of **2** and **4** relevant to guest exchange. As shown in Supplementary Fig. 96, the apertures within the interior tetrahedral core of **4** exhibit a reduced proton–proton distance between adjacent faces compared to those in framework **2**, corresponding to a decrease of approximately 0.3 Å in largest aperture width. These observations indicated that the steric hindrance imparted by the woven ligand framework mechanically suppresses the dynamic motion of the tetrahedral core of **4**, inducing a contraction in average aperture size. This reduction in aperture^46^ is likely to decrease the frequency of effective collisions between ReO_4_^−^and TfO^−^⊂**2**, thereby more effectively trapping TfO^−^ within the cavity.

We further investigated an additional guest pair, SbF_6_^−^ and TfO^−^, to demonstrate the broader applicability of this system, as presented in Supplementary Figs. 59–61. Upon the introduction of SbF_6_^−^, which is expected to exhibit a weaker binding affinity than ReO_4_^−^ (ref. ^41^), the guest exchange behaviour follows a trend similar to that observed for the previously investigated ReO_4_^−^/ TfO^−^ pair. In knotted cage **4**, TfO^−^ remained mechanically locked within the framework, leading to only ~7% conversion observed after 1,128 h at room temperature. Conversely, in structure **2**, guest exchange proceeded at a notably accelerated rate, reaching approximately 42% conversion within 52.5 min.

We then investigated whether knotted cages **3** and **4** exhibited greater structural stability than their congeners **1** and **2**, thereby conferring greater robustness against chemical stimuli. Their tolerance for dimethyl sulfoxide (DMSO) and water was probed, as DMSO may coordinate competitively to Zn^II^, while water can cleave imine bonds. As shown in Supplementary Figs. 62–65, upon addition of 20 μL deuterated DMSO to an acetonitrile solution of **1**, the structure transformed into **2**, attributed to the loss of Zn^II^ ions. By contrast, knotted **3**, which is structurally related to **1**, remained stable upon addition of the same amount of DMSO. When 120 μL deuterated DMSO was added to **2**, complete decomposition occurred. However, its knotted counterpart **4** remained entirely intact under the same conditions. The effects of water were also examined, as shown in Supplementary Figs. 66–69. Upon the introduction of 10 and 50 μL D_2_O to **1** and **2**, respectively, complete decomposition occurred, regenerating subcomponents **A** and **B**. By contrast, knotted analogues **3** and **4** remained predominantly intact upon exposure to analogous amounts of D_2_O.

The stability of **4** in the presence of the acid HCl and the oxidant H_2_O_2_ was also explored (Supplementary Figs. 70–72). Trefoil tetrahedron **4** was stable to treatment with excess (150 equiv.) H_2_O_2_, whereas it underwent decomposition in the presence of excess HCl (18 equiv.), accompanied by simultaneous guest release. This remarkable stability of knotted cage **4**, along with its ability to encapsulate guests, suggests potential applications in controlled delivery systems, where acidic chemical stimuli could trigger targeted release.

**Fig. 5:**
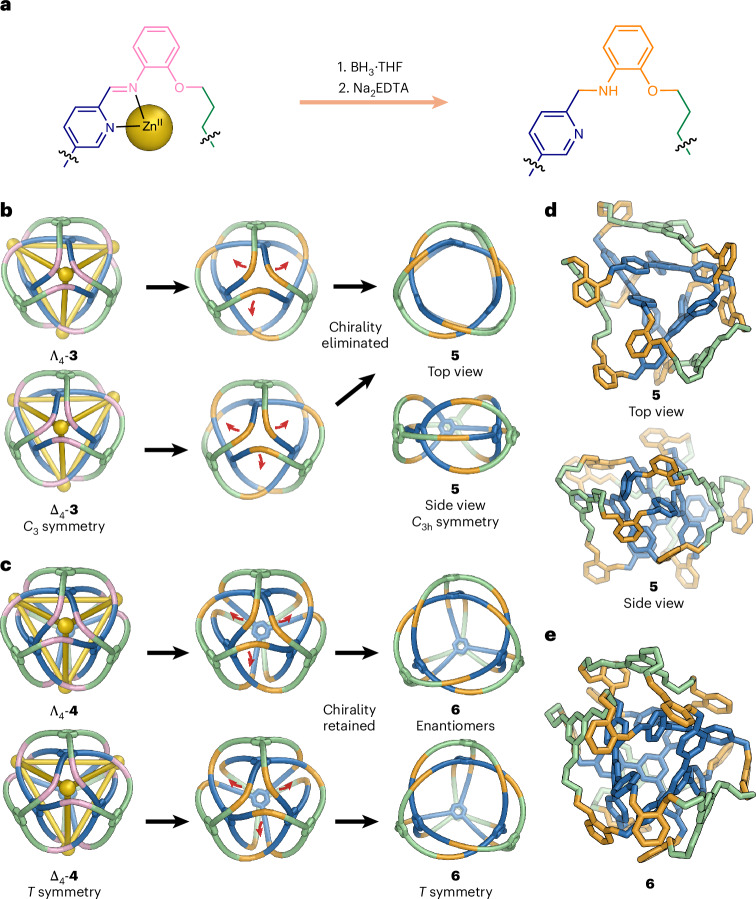
Preparation of 5 and 6. **a**, Reduction and demetallation of the imine bonds in **3** and **4**. **b**,**c**, Schematic illustrations of reduction and demetallation of **3** (**b**) and **4** (**c**). The red arrows indicate how the organic backbone structure moves during structural transformations. **d**, GFN2-xTB^48^-optimized structure of **5**, calculated using GMIN. **e**, GFN2-xTB^48^-optimized structure of **6** (from Δ_4_-**4**), determined with GMIN.

As shown in Fig. 5, we explored the reduction and demetallation of **3** and **4** to obtain the fully organic covalent-interwoven structures **5** and **6**, which proceeded with yields of 87 and 55%, respectively. The stereochemistry of the Zn^II^ centres of **3** led to the formation of a racemic mixture of two enantiomers, as shown in Fig. 5b. However, during the transformation from **3** into **5**, the emergence of a symmetry plane in **5** induced a transition from *C*_3_ symmetry to time-averaged *C*_3h_ symmetry, leading to the elimination of its chirality, and thus yielding achiral organic trefoil perplexane **5** (Fig. 5b, Supplementary Fig. 81 and Supplementary Videos 1 and 2) Treatment of **3** with trihydrido(tetrahydrofuran)boron (BH_3_·THF) and disodium ethylenediaminetetraacetate (Na_2_EDTA) in a mixed acetonitrile/methanol solution transformed the dynamic imine linkages into stable secondary amines, as confirmed by NMR spectra and matrix-assisted laser desorption/ionization time-of-flight (MALDI-TOF) mass spectrometry (Supplementary Figs. 73–80). DOSY ^1^H NMR gave a solvodynamic radius of 8.4 Å (Supplementary Fig. 79). Thisvalue is consistent with the calculated structure of **5**, which was obtained by basin-hopping^47^ global optimization using the GMIN program at the GFN-FF^37^ level followed by relaxation at the GFN2-xTB^48^ level, as shown in Fig. 5d.

The four zinc(II) vertices of trefoil tetrahedron **4** possess either all Δ or all Λ handedness, with **4** forming as a racemic mixture of its two enantiomers. In contrast to **3**, the chirality of **4** is topological in nature, being linked to the specific over/under pathways followed by its ligand strands as they weave together. In contrast to the chirality elimination observed in the transformation from **3** to **5**, this topological chirality of **4** is preserved even after reduction and demetallation (Fig. 5c), as the reduction and demetallation of the vertices does not alter the connectivity of the structure^17,18^. This procedure thus yields the fully covalently linked organic trefoil tetrahedron **6** (Fig. 5c) as a racemate. In contrast with elegant peptide-based structures reported by Fujita and co-workers^49,50^, the structure of **6**, along with that of its precursor **4**, possesses topology that is established along covalent-bond pathways, without considering any labile coordinative bonds. The structure of **6** was confirmed by NMR spectroscopy and MALDI-TOF mass spectrometry (Supplementary Figs. 82–89). Only one environment per ligand proton of **6** was observed in the ^1^H NMR spectrum, as with **4**, suggesting that the *T‑*symmetric structure of **4** was retained in **6**, as shown in Supplementary Fig. 82. The ¹H DOSY spectrum of **6** indicated a solvodynamic radius of 11.2 Å (Supplementary Fig. 88). As shown in Fig. 5e, the optimized geometry of **6** was obtained using basin-hopping implemented in the GMIN program at the GFN-FF^37^ level, followed by further optimizations at the GFN2-xTB^48^ level, producing a compact structure consistent in size with our DOSY results.

## Conclusion

The construction of interwoven knotted cages **3** and **4**, using a simple, one-pot, high-yielding procedure underscores the effectiveness of subcomponent self-assembly in creating entangled, topologically complex species. Our design strategy of bridging over the panels of metal–organic cage cores could be extended for construction of other interwoven frameworks. The observation that knotting causes cage **4** to release its guest 17,000 times more slowly than non-knotted congener **2** suggests that these cages may find use in controlled-release applications. The transformations of **3** and **4** into fully covalently linked structures **5** and **6** also show the utility of our methods to prepare metal-free complex knotted structures, with the topological chirality of structures such as **6** potentially leading to applications in chiral recognition. Future work will probe these applications, as well as the transfer of stereochemical information during the formation and reduction of these structures. The ability to control the geometrical configuration and topological chirality of these structures will advance the design of woven materials and molecular devices.

## Methods

### Self assembly of 3

**A** (5.0 mg, 1.0 equiv., 12.7 µmol), **C** (8.6 mg, 1.1 equiv., 14.0 µmol) and zinc(II) trifluoromethanesulfonate (6.2 mg, 1.3 equiv., 16.9 µmol) were mixed in 2 mL acetonitrile. The reaction mixture was heated and stirred in a microwave reactor for 2 h at 130 °C. Then the mixture was concentrated to 0.5 mL and diethyl ether (14 mL) was added. The precipitate was collected by centrifugation and washed two times with excess diethyl ether to give **3** in 91% yield (16.7 mg).

### Self assembly of 4

**A** (5.0 mg, 1.0 equiv., 12.7 µmol), **C** (8.6 mg, 1.1 equiv., 14.0 µmol), potassium trifluoromethanesulfonate (12.0 mg, 5.0 equiv., 63.5 µmol) and zinc(II) trifluoromethanesulfonate (4.6 mg, 1.0 equiv., 12.7 µmol) were mixed in 2 mL acetonitrile. The reaction mixture was heated and stirred in a microwave reactor for 4 h at 150 °C. Then the mixture was concentrated to 0.5 mL and diethyl ether (14 mL) was added. The precipitate was collected by centrifugation and washed two times with excess diethyl ether. Flash size-exclusion chromatography was used to remove excess potassium trifluoromethanesulfonate, giving **4** in 79% yield (13.2 mg).

### Synthesis of 5

To a stirred solution of **3** (15 mg, 3.5 µmol, 1 equiv.) in MeCN/MeOH (5/1, v/v, 2 mL total volume) at room temperature was added BH_3_·THF (1 M, 400 µL, 400 µmol, 12 equiv. per imine). The mixture was stirred for 2 h during which time the colour of the solution changed from orange to light yellow. CH_2_Cl_2_ (30 mL) and Na_2_EDTA (10 mg) were then added and the mixture was stirred for 10 min. The resulting suspension was poured into 30 mL H_2_O and the mixture was extracted with 10 × 5 mL CH_2_Cl_2_. The combined organic layers were filtered over cotton covered with sand and then the solvent was removed under reduced pressure. The residue was washed with methanol three times (3 × 10 mL) and dried under reduced pressure affording the reduced and demetallated structure **5** (8.6 mg, 3.0 µmol, 87%).

### Synthesis of 6

To a stirred solution of **4** (20 mg, 3.8 µmol, 1 equiv.) in MeCN/MeOH (5/1, v/v, 2 mL total volume) at room temperature was added BH_3_·THF (1 M, 500 µL, 500 µmol, 11 equiv. per imine). The mixture was stirred for 2 h during which time the colour of the solution changed from yellow to light yellow. CH_2_Cl_2_ (30 mL) and Na_2_EDTA (10 mg) were then added and the mixture was stirred for 10 min. The resulting suspension was poured into 30 mL H_2_O and the mixture was extracted with 10 × 5 mL CH_2_Cl_2_. The combined organic layers were filtered over cotton covered with sand and then the solvent was removed under reduced pressure. The residue was washed with methanol three times (3 × 10 mL) and dried under reduced pressure affording the reduced and demetallated structure **6** (8.0 mg, 2.1 µmol, 55%).

## Supplementary information

Supplementary Information Supplementary Figs. 1–96 and Tables 1–5.

Supplementary Data 1 Crystallographic data for **3**; (CCDC reference 2415403).

Supplementary Data 2 Crystallographic data for **4**; (CCDC reference 2415402).

Supplementary Data 3 Cartesian coordinates of structure **5**.

Supplementary Data 4 Cartesian coordinates of structure **6**.

Supplementary Video 1 Side view video of molecular dynamics simulation of **5**.

Supplementary Video 2 Top view video of molecular dynamics simulation of **5**.

Supplementary Video 3 Side view video of molecular dynamics simulation of **6**.

Supplementary Video 4 Top view video of molecular dynamics simulation of **6**.

## Source data

Source Data Fig. 4 Statistical source data of guest displacement.

## Data Availability

All data needed to evaluate the conclusions are included in the paper and Supplementary Information. Crystallographic data for the structures reported in this Article have been deposited at the Cambridge Crystallographic Data Centre, under deposition numbers CCDC 2415403 (**3**) and 2415402 (**4**). Copies of the data can be obtained free of charge via https://www.ccdc.cam.ac.uk/structures/. Source data are provided with this paper.

## References

[1] Postrel, V. *The Fabric of Civilization: How Textiles Made the World* (Basic Books, 2020).

[2] Zhang, L., Marcos, V. & Leigh, D. A. Molecular machines with bio-inspired mechanisms. *Proc. Natl Acad. Sci. USA* **115**, 9397–9404 (2018). 29483259 10.1073/pnas.1712788115 PMC6156679

[3] Sułkowska, J. I., Rawdon, E. J., Millett, K. C., Onuchic, J. N. & Stasiak, A. Conservation of complex knotting and slipknotting patterns in proteins. *Proc. Natl Acad. Sci. USA* **109**, E1715–E1723 (2012). 22685208 10.1073/pnas.1205918109 PMC3387036

[4] Wasserman, S. A. & Cozzarelli, N. R. Biochemical topology: applications to DNA recombination and replication. *Science* **232**, 951–960 (1986). 3010458 10.1126/science.3010458

[5] Wikoff, W. R. et al. Topologically linked protein rings in the bacteriophage HK97 capsid. *Science* **289**, 2129–2133 (2000). 11000116 10.1126/science.289.5487.2129

[6] Dietrich‐Buchecker, C. O. & Sauvage, J. A synthetic molecular trefoil knot. *Angew. Chem. Int. Ed.* **28**, 189–192 (1989).

[7] Prakasam, T. et al. Simultaneous self-assembly of a [2]catenane, a trefoil knot, and a Solomon link from a simple pair of ligands. *Angew. Chem. Int. Ed.* **52**, 9956–9960 (2013). 10.1002/anie.201302425 23832610

[8] Wood, C. S., Ronson, T. K., Belenguer, A. M., Holstein, J. J. & Nitschke, J. R. Two-stage directed self-assembly of a cyclic [3]catenane. *Nat. Chem.* **7**, 354–358 (2015). 25803475 10.1038/nchem.2205

[9] Li, H. et al. Quantitative self-assembly of a purely organic three-dimensional catenane in water. *Nat. Chem.* **7**, 1003–1008 (2015). 26587716 10.1038/nchem.2392

[10] Liu, Y., O’Keeffe, M., Treacy, M. J. M. & Yaghi, O. M. The geometry of periodic knots, polycatenanes and weaving from a chemical perspective: a library for reticular chemistry. *Chem. Soc. Rev.* **47**, 4642–4664 (2018). 29726872 10.1039/c7cs00695k

[11] Ashbridge, Z. et al. Knotting matters: orderly molecular entanglements. *Chem. Soc. Rev.* **51**, 7779–7809 (2022). 35979715 10.1039/d2cs00323f PMC9486172

[12] Chichak, K. S. et al. Molecular Borromean rings. *Science* **304**, 1308–1312 (2004). 15166376 10.1126/science.1096914

[13] Zhu, R., Lübben, J., Dittrich, B. & Clever, G. H. Stepwise halide-triggered double and triple catenation of self-assembled coordination cages. *Angew. Chem. Int. Ed.* **54**, 2796–2800 (2015). 10.1002/anie.201408068 25395277

[14] Schalley, C. A. Borromean rings: a one‐pot synthesis. *Angew. Chem. Int. Ed.* **43**, 4399–4401 (2004). 10.1002/anie.200460583 15317001

[15] Leigh, D. A. et al. A molecular endless (74) knot. *Nat. Chem.* **13**, 117–122 (2021). 33318672 10.1038/s41557-020-00594-x

[16] Cougnon, F. B. L., Caprice, K., Pupier, M., Bauzá, A. & Frontera, A. A strategy to synthesize molecular knots and links using the hydrophobic effect. *J. Am. Chem. Soc.* **140**, 12442–12450 (2018). 30152696 10.1021/jacs.8b05220

[17] Wu, L. et al. Synthesis of contra-helical trefoil knots with mechanically tuneable spin-crossover properties. *Nat. Synth.* **2**, 17–25 (2022).

[18] Carpenter, J. P. et al. Controlling the shape and chirality of an eight-crossing molecular knot. *Chem* **7**, 1534–1543 (2021).

[19] Liu, Y. et al. Weaving of organic threads into a crystalline covalent organic framework. *Science* **351**, 365–369 (2016). 26798010 10.1126/science.aad4011

[20] Leigh, D. A. et al. Tying different knots in a molecular strand. *Nature* **584**, 562–568 (2020). 32848222 10.1038/s41586-020-2614-0

[21] August, D. P. et al. Transmembrane ion channels formed by a star of David [2]catenane and a molecular pentafoil knot. *J. Am. Chem. Soc.* **142**, 18859–18865 (2020). 33084320 10.1021/jacs.0c07977 PMC7745878

[22] Prakasam, T. et al. Metal–organic self-assembled trefoil knots for C—Br bond activation. *ACS Catal.* **9**, 1907–1914 (2019).

[23] Zhang, M. et al. Mechanical scission of a knotted polymer. *Nat. Chem.* **16**, 1366–1372 (2024). 38649468 10.1038/s41557-024-01510-3 PMC11321991

[24] Olenyuk, B., Levin, M. D., Whiteford, J. A., Shield, J. E. & Stang, P. J. Self-assembly of nanoscopic dodecahedra from 50 predesigned components. *J. Am. Chem. Soc.* **121**, 10434–10435 (1999).

[25] Wang, H. et al. Hierarchical self-assembly of nanowires on the surface by metallo-supramolecular truncated cuboctahedra. *J. Am. Chem. Soc.* **143**, 5826–5835 (2021). 33848163 10.1021/jacs.1c00625

[26] Fujita, D. et al. Self-assembly of M_30_L_60_ icosidodecahedron. *Chem* **1**, 91–101 (2016).

[27] Koo, J. et al. Gigantic porphyrinic cages. *Chem* **6**, 3374–3384 (2020).

[28] Wu, K. et al. Systematic construction of progressively larger capsules from a fivefold linking pyrrole-based subcomponent. *Nat. Synth.* **2**, 789–797 (2023).

[29] Rizzuto, F. J., Carpenter, J. P. & Nitschke, J. R. Multisite binding of drugs and natural products in an entropically favorable, heteroleptic receptor. *J. Am. Chem. Soc.* **141**, 9087–9095 (2019). 31079455 10.1021/jacs.9b03776

[30] Sudan, S. et al. Identification of a heteroleptic Pd_6_L_6_L′_6_ coordination cage by screening of a virtual combinatorial library. *J. Am. Chem. Soc.* **143**, 1773–1778 (2021). 33476512 10.1021/jacs.0c12793

[31] Zhang, D., Ronson, T. K., Zou, Y.-Q. & Nitschke, J. R. Metal–organic cages for molecular separations. *Nat. Rev. Chem.* **5**, 168–182 (2021). 37117530 10.1038/s41570-020-00246-1

[32] Kreno, L. E. et al. Metal–organic framework materials as chemical sensors. *Chem. Rev.* **112**, 1105–1125 (2012). 22070233 10.1021/cr200324t

[33] Saha, R., Mondal, B. & Mukherjee, P. S. Molecular cavity for catalysis and formation of metal nanoparticles for use in catalysis. *Chem. Rev.* **122**, 12244–12307 (2022). 35438968 10.1021/acs.chemrev.1c00811

[34] Mal, P., Breiner, B., Rissanen, K. & Nitschke, J. R. White phosphorus is air-stable within a self-assembled tetrahedral capsule. *Science* **324**, 1697–1699 (2009). 19556504 10.1126/science.1175313

[35] Forgan, R. S., Sauvage, J.-P. & Stoddart, J. F. Chemical topology: complex molecular knots, links, and entanglements. *Chem. Rev.* **111**, 5434–5464 (2011). 21692460 10.1021/cr200034u

[36] Bergman, H. M. et al. Discovery of an interlocked and interwoven molecular topology in nanocarbons via dynamic C–C bond formation. *J. Am. Chem. Soc.* **147**, 19132–19138 (2025). 40408623 10.1021/jacs.5c04268 PMC12147124

[37] Spicher, S. & Grimme, S. Robust atomistic modeling of materials, organometallic, and biochemical systems. *Angew. Chem. Int. Ed.* **59**, 15665–15673 (2020). 10.1002/anie.202004239 PMC7267649 32343883

[38] Endo, K., Ube, H. & Shionoya, M. Multi-stimuli-responsive interconversion between bowl- and capsule-shaped self-assembled zinc(II) complexes. *J. Am. Chem. Soc.* **142**, 407–416 (2020). 31804816 10.1021/jacs.9b11099

[39] Mirtschin, S., Slabon-Turski, A., Scopelliti, R., Velders, A. H. & Severin, K. A coordination cage with an adaptable cavity size. *J. Am. Chem. Soc.* **132**, 14004–14005 (2010). 20860361 10.1021/ja1063789

[40] Freye, S. et al. Template control over dimerization and guest selectivity of interpenetrated coordination cages. *J. Am. Chem. Soc.* **135**, 8476–8479 (2013). 23697828 10.1021/ja403184a

[41] Castilla, A. M., Ronson, T. K. & Nitschke, J. R. Sequence-dependent guest release triggered by orthogonal chemical signals. *J. Am. Chem. Soc.* **138**, 2342–2351 (2016). 26799196 10.1021/jacs.5b13016

[42] Lehn, J.-M. Supramolecular chemistry: receptors, catalysts, and carriers. *Science* **227**, 849–856 (1985). 17821215 10.1126/science.227.4689.849

[43] Zhu, H. et al. Applications of macrocycle-based solid-state host–guest chemistry. *Nat. Rev. Chem.* **7**, 768–782 (2023). 37783822 10.1038/s41570-023-00531-9

[44] Geng, W.-C., Sessler, J. L. & Guo, D.-S. Supramolecular prodrugs based on host–guest interactions. *Chem. Soc. Rev.* **49**, 2303–2315 (2020). 32181453 10.1039/c9cs00622b

[45] Vanommeslaeghe, K. et al. CHARMM general force field: a force field for drug‐like molecules compatible with the CHARMM all‐atom additive biological force fields. *J. Comput. Chem.* **31**, 671–690 (2010). 19575467 10.1002/jcc.21367 PMC2888302

[46] Pluth, M. D. & Raymond, K. N. Reversible guest exchange mechanisms in supramolecular host–guest assemblies. *Chem. Soc. Rev.* **36**, 161–171 (2007). 17264920 10.1039/b603168b

[47] Wales, D. J. & Doye, J. P. K. Global optimization by basin-hopping and the lowest energy structures of Lennard-Jones clusters containing up to 110 atoms. *J. Phys. Chem. A* **101**, 5111–5116 (1997).

[48] Bannwarth, C., Ehlert, S. & Grimme, S. GFN2-xTB—an accurate and broadly parametrized self-consistent tight-binding quantum chemical method with multipole electrostatics and density-dependent dispersion contributions. *J. Chem. Theory Comput.* **15**, 1652–1671 (2019). 30741547 10.1021/acs.jctc.8b01176

[49] Sawada, T. & Fujita, M. Folding and assembly of metal-linked peptidic nanostructures. *Chem* **6**, 1861–1876 (2020).

[50] Domoto, Y., Abe, M. & Fujita, M. A highly entangled (M_3_L_2_)_8_ truncated cube from the anion-controlled oligomerization of a π-coordinated M_3_L_2_ subunit. *J. Am. Chem. Soc.* **143**, 8578–8582 (2021). 34100600 10.1021/jacs.1c03208

