# Supplementary material for: Synthesis of covalently linked knotted cage frameworks
Source: Nat Synth. 2025 Jun 25;4(10):1270–7. doi: 10.1038/s44160-025-00822-7 (PMC12513843; doi:10.1038/s44160-025-00822-7)
Supplement: Supplementary file 1 — Supplementary Figs. 1–96 and Tables 1–5. [file 44160_2025_822_MOESM1_ESM.pdf]

nature synthesis

Supplementary information

https://doi.org/10.1038/s44160-025-00822-7

Synthesis of covalently linked knotted cage frameworks

In the format provided by the authors and unedited

Table of Contents

1. General 3

2. Subcomponent synthesis and characterisation 4

3. Self-assembly of coordination architectures 6

3.1 Self-assembly of **1** 6

3.2 Self-assembly of **2** 13

3.2 Self-assembly of **3** 20

3.4 Self-assembly of **4** 27

4. Guest exchange investigations 35

4.1 General methods 35

4.2 NMR spectra of the anionic guest exchange experiments (TfO^- and ReO_4^-) 35

4.3 Calculations of $\Delta H^‡$ and $\Delta S^‡$ of guest exchange 43

4.4 Calculations of apparent activation energy and pre-exponential factor 48

4.5 Proposed mechanism 50

4.5 NMR spectra of the anionic guest exchange experiments (TfO^- and SbF_6^-) 52

5. Robustness investigations 53

5.1 Experimental details 53

5.2 Addition of d_6-DMSO 53

5.3 Addition of D_2O 56

5.4 Addition of HCl 58

5.5 Addition of H_2O_2 59

6. Reduction and demetallation of 3 and 4 60

6.1 Reduction and demetallation of **3** 60

6.2 Reduction and demetallation of **4** 67

7. X-ray Crystallography 73

8. Calculations 80

8.1 General procedure and discussions 80

8.2 Input files 89

9. References 92

1. General

Materials and methods

Unless otherwise stated, all chemicals were obtained from commercial sources and used as received. **A** was prepared according to a literature procedures.[1] A mixture of 1,3,5-tris(4,4,5,5-tetramethyl-1,3,2-dioxaborolan-2-yl)benzene (300 mg, 0.66 mmol), 5-bromo-2-(dimethoxymethyl)pyridine (0.57 g, 2.1 mmol), K_2CO_3 (1.3 g, 9.7 mmol), and $Pd(PPh_3)_4$ (223 mg, 0.19 mmol) in anhydrous DMF (45 mL) was stirred under a nitrogen atmosphere at 90 °C for 24 h. Then, the solvent was removed, and the residue was extracted with chloroform 3 times. The combined organic phases were filtered and concentrated under reduced pressure. The crude product was purified by flash chromatography on silica gel (hexane/EtOAc 1:1, then EtOAc). The resulting product was suspended in a mixture of AcOH (1.5 mL) and H_2O (3.0 mL) and refluxed with vigorous stirring for 2 h. After cooling to room temperature, the reaction mixture was neutralized with saturated aqueous $NaHCO_3$ and evaporated to dryness. The resulting solid was dissolved in chloroform; after washing with water and brine, the combined organic layers were concentrated under reduced pressure to afford compound **A** as a white solid (186 mg, 72%) without further purification.

Nuclear Magnetic Resonance (NMR)

NMR experiments were measured on Bruker AVANCE III and NEO (400 and 500 MHz) spectrometers. Chemical shifts for ^1H and ^{13}C NMR are reported in ppm with residual solvent as reference: Acetonitrile (1.94 ppm for ^1H, 1.32 ppm for ^{13}C). Abbreviations for signal multiplicity of ^1H NMR spectra are shown as following: s: singlet, d: doublet, t: triplet, dd: doublet of doublets; dt: doublet of triplets; m: multiplet, br: broad.

Mass spectrometry (MS)

Low-resolution electrospray ionization mass spectrometry (LR ESI-MS) was undertaken on a Waters XevoTQD (cone voltage 5-20 eV; desolvation temperature 307 K; ionization temperature 325 K), infused from a Harvard syringe pump at a rate of 10 $\mu L \cdot min^{-1}$. High-resolution electrospray ionization mass spectrometry (HR ESI-MS) was undertaken on a Waters Synapt G2-Si mass spectrometer. MALDI-TOF MS was undertaken on NMSF's Bruker ultrafleXtreme MALDI-TOF MS. CsI_3 in DCTB/THF matrix was used for calibration of the mass range in reflectron (*m/z* 1000-5000) positive ion modes. A small amount of each of the samples was dissolved at ~1 mg/ml in DCM. The DCTB matrix

was prepared at 10 mg/ml in MeCN. Analysis mixtures of either a ratio 1:10:1 sample: matrix: NaOAc or 1:10 sample: matrix was prepared, vortexed then 0.5μl spotted onto a ground steel target for measurement. DCTB matrix was selected as it generally requires very low laser energy to generate ions, minimizing potential fragmentation (and/or metastable formation) NaOAc was added with the intention to preferentially promote $[M+Na]^+$ formation.

2. Subcomponent synthesis and characterisation

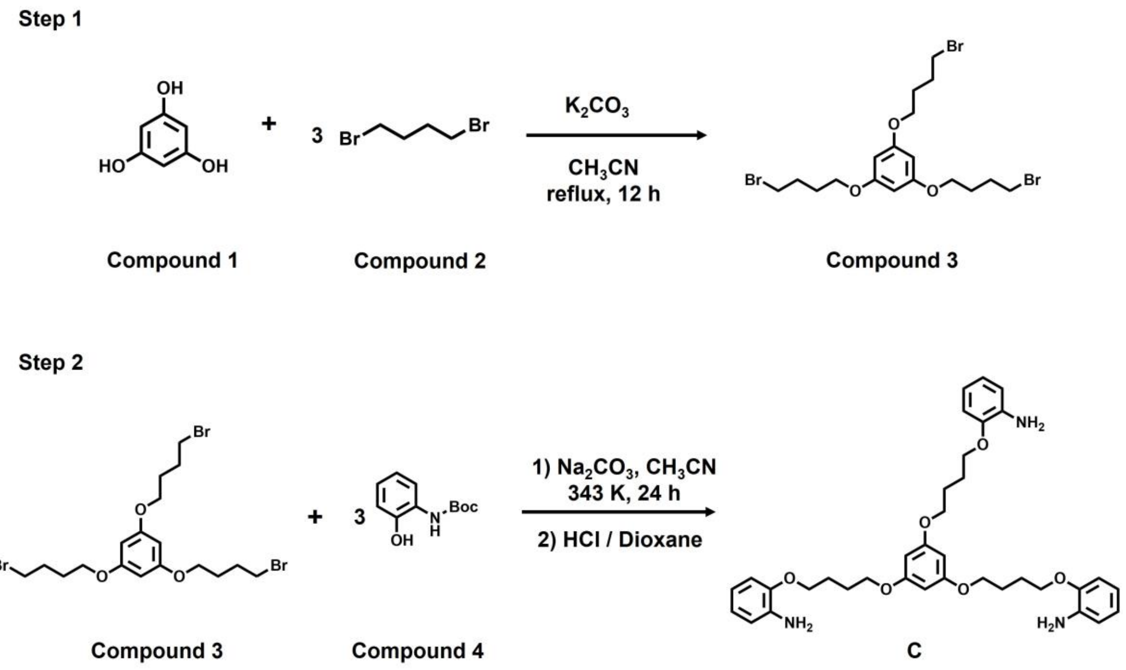

Supplementary Scheme 1 | Synthesis of **C**.

Step 1:

Compound **3** was synthesized according to the literature.[2]

Step 2:

To a Schlenk flask were added Compound **3** (100 mg, 0.19 mmol, 1.0 equiv), Compound **4** (118 mg, 0.57 mmol, 3.0 equiv), and Na_2CO_3 (120 mg, 1.14 mmol, 6.0 equiv). Then 20 mL acetonitrile was added to the mixture. The resulting mixture was then heated at 70 °C for 24 h. The solvent was removed under reduced pressure, and then CH_2Cl_2 and water were added. The aqueous layer was extracted three times with CH_2Cl_2 and then washed with brine. The organic phases were combined and dried over Na_2SO_4, filtered, and concentrated under reduced pressure. Then 10 mL 4 M HCl/Dioxane and 10 mL $CHCl_3$

were add and the mixture stirred for 12 h at room temperature. The aqueous layer was neutralized with Na_2CO_3 aqueous solution and washed three times with CH_2Cl_2/H_2O. The organic phases were combined and dried over Na_2SO_4, filtered, and concentrated under reduced pressure. The crude product was purified by silica gel column chromatography with EtOAc/Hexane (v/v 1:10 to 1:1) to give **C** as a brown sticky solid (73 mg, 63%).

^{1}H NMR (500 MHz, $CDCl_3$) δ 6.83 – 6.79 (m, 6H), 6.76 – 6.71 (m, 6H), 6.10 (s, 3H), 4.09 (t, J = 5.8 Hz, 6H), 4.01 (t, J = 5.8 Hz, 6H), 2.05 – 1.96 (m, 12H).
^{13}C NMR (126 MHz, $CDCl_3$) δ 160.8, 146.6, 136.3, 121.1, 118.5, 115.1, 111.5, 94.0, 77.3, 77.0, 76.8, 67.7, 67.5, 26.1, 26.1.
ESI-MS (CH_3CN) m/z: $[M+H]^+$ calc. for $C_{36}H_{46}N_3O_6$, 616.33, found 616.34.

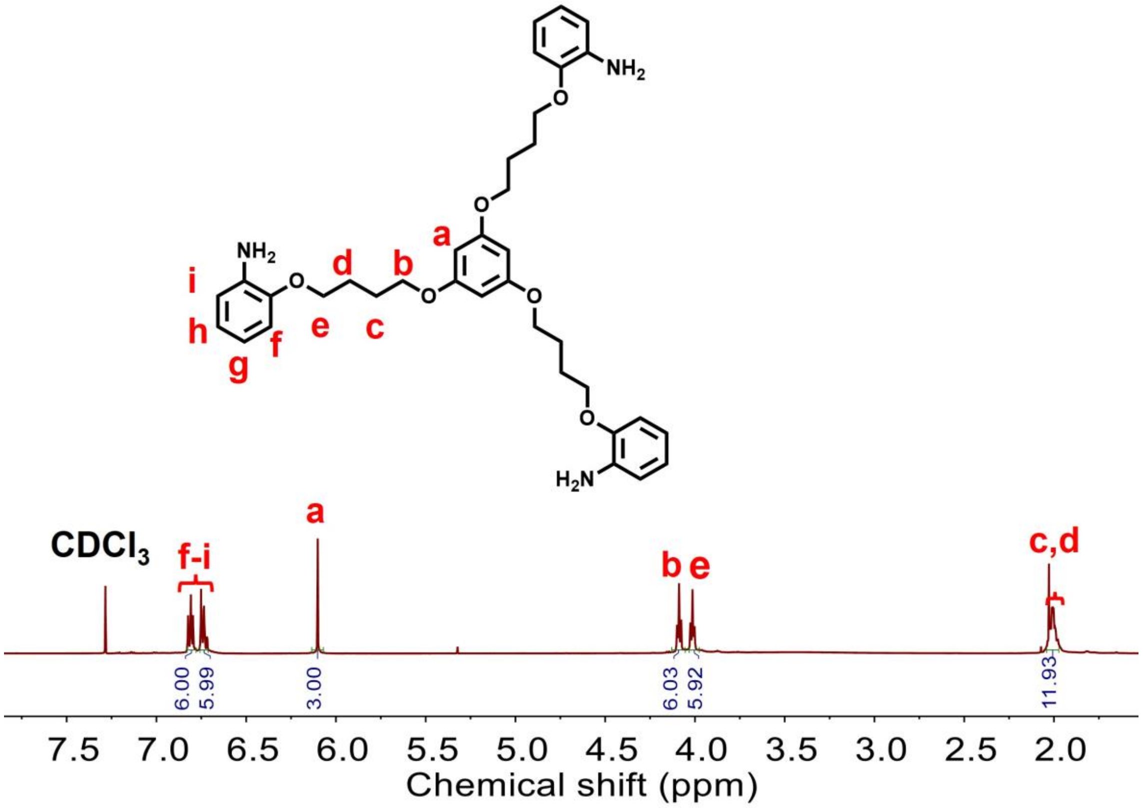

Supplementary Figure 1 | ^{1}H NMR spectrum (500 MHz, 298K, $CDCl_3$) of **C**.

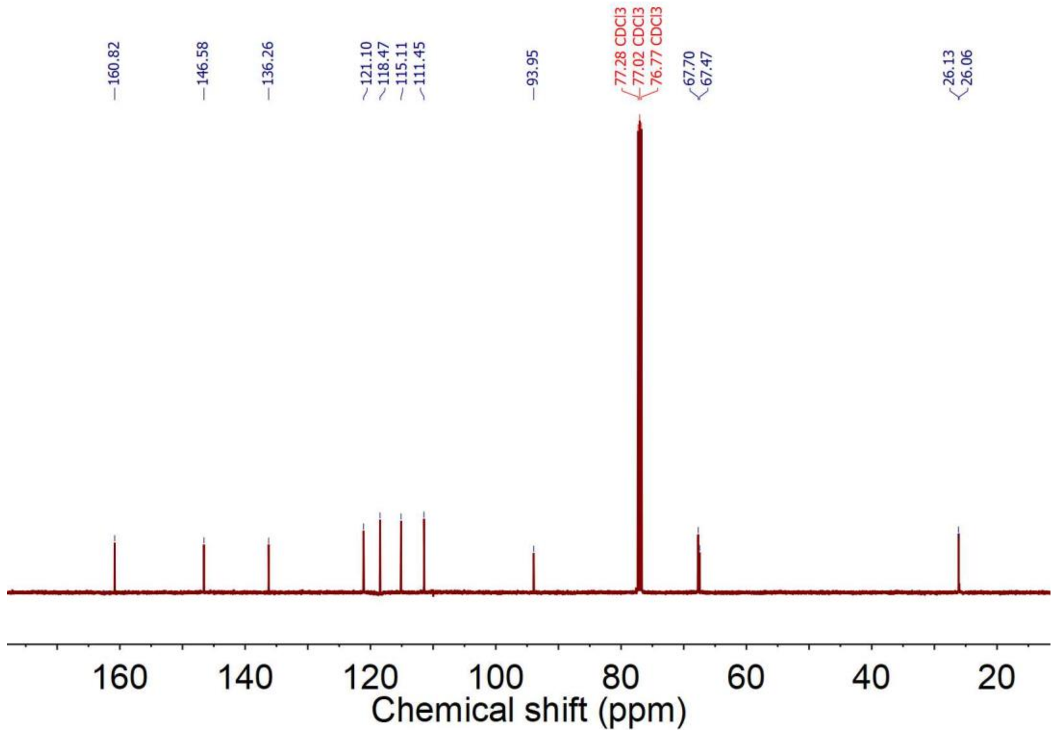

Supplementary Figure 2 | ^{13}C NMR spectrum (126 MHz, 298K, $CDCl_3$) of **C**.

3. Self-assembly of coordination architectures

3.1 Self-assembly of **1**

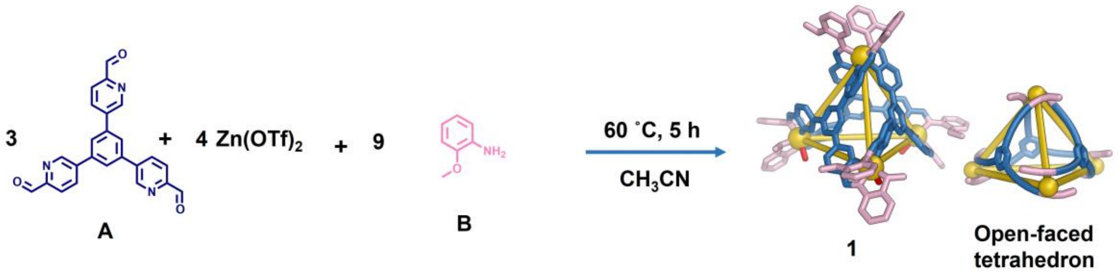

Supplementary Scheme 2 | Construction of **1**

A (5.0 mg, 1.0 equiv, 12.7 µmol), **B** (5.2 mg, 3.3 equiv, 41.9 µmol) and zinc (II) trifluoromethanesulfonate (6.2 mg, 1.3 equiv, 16.9 µmol) were mixed in 2 mL acetonitrile. The reaction mixture was heated and stirred in a microwave reactor for 5 h at 60 ˚C. Then the mixture was concentrated to 0.5 mL and diethyl ether (14 mL) was added. The precipitate was collected by centrifugation and washed two times with excess diethyl ether to give **1** in 82 % yield (12.4 mg).

^{1}H NMR (500 MHz, CD_3CN) δ 9.30 (s, 3H), 9.27 (d, J = 2.0 Hz, 3H), 9.10 (s, 3H), 8.78 (dd, J = 8.3, 2.1 Hz, 3H), 8.73-8.70 (m, 6H), 8.65 (dd, J = 8.1, 2.1 Hz, 3H), 8.49 (d, J =

8.1 Hz, 3H), 8.40 (d, J = 8.1 Hz, 3H), 8.33 – 8.26 (m, 9H), 7.80 – 7.74 (m, 6H), 7.72 – 7.67 (m, 6H), 7.43 (t, J = 7.7 Hz, 3H), 7.38 (t, J = 7.7 Hz, 3H), 7.19 (t, J = 7.7 Hz, 3H), 7.14 (dd, J = 7.8, 1.6 Hz, 3H), 7.11 – 7.06 (m, 12H), 6.88 (t, J = 7.7 Hz, 3H), 6.83 (d, J = 8.4 Hz, 3H), 6.74 (t, J = 7.6 Hz, 3H), 5.36 (dd, J = 7.8, 1.6 Hz, 2H), 3.85 (s, 9H), 3.33 (s, 9H), 3.21 (s, 9H).

^{13}C NMR (126 MHz, CD_3CN) δ 166.0, 162.2, 153.5, 152.0, 151.4, 151.0, 148.7, 147.4, 147.2, 146.8, 146.6, 146.5, 141.2, 140.7, 140.4, 139.7, 139.4, 138.6, 138.3, 137.9, 137.3, 136.3, 134.5, 132.7, 130.5, 130.3, 130.3, 129.9, 129.4, 128.9, 128.9, 127.5, 127.4, 127.4, 122.5, 122.4, 121.8, 121.2, 120.9, 120.8 (m, TfO^-), 118.5, 117.3, 112.6, 111.8, 111.7, 56.7, 55.2, 55.1.

^{19}F NMR (376 MHz, CD_3CN) δ –79.31 (s, CF_3).

ESI-MS (CH_3CN) for 1: *m/z* = 362.5 $[\mathbf{1}\cdot(TfO^-)_1]^{7+}$, 447.8 $[\mathbf{1}\cdot(TfO^-)_2]^{6+}$, 567.0 $[\mathbf{1}\cdot(TfO^-)_3]^{5+}$, 746.2 $[\mathbf{1}\cdot(TfO^-)_4]^{4+}$,1045.0 $[\mathbf{1}\cdot(TfO^-)_5]^{3+}$. Calculated peaks: m/z = 362.4 $[\mathbf{1}\cdot(TfO^-)_1]^{7+}$, 447.7 $[\mathbf{1}\cdot(TfO^-)_2]^{6+}$, 567.0 $[\mathbf{1}\cdot(TfO^-)_3]^{5+}$, 746.1 $[\mathbf{1}\cdot(TfO^-)_4]^{4+}$,1044.5 $[\mathbf{1}\cdot(TfO^-)_5]^{3+}$.

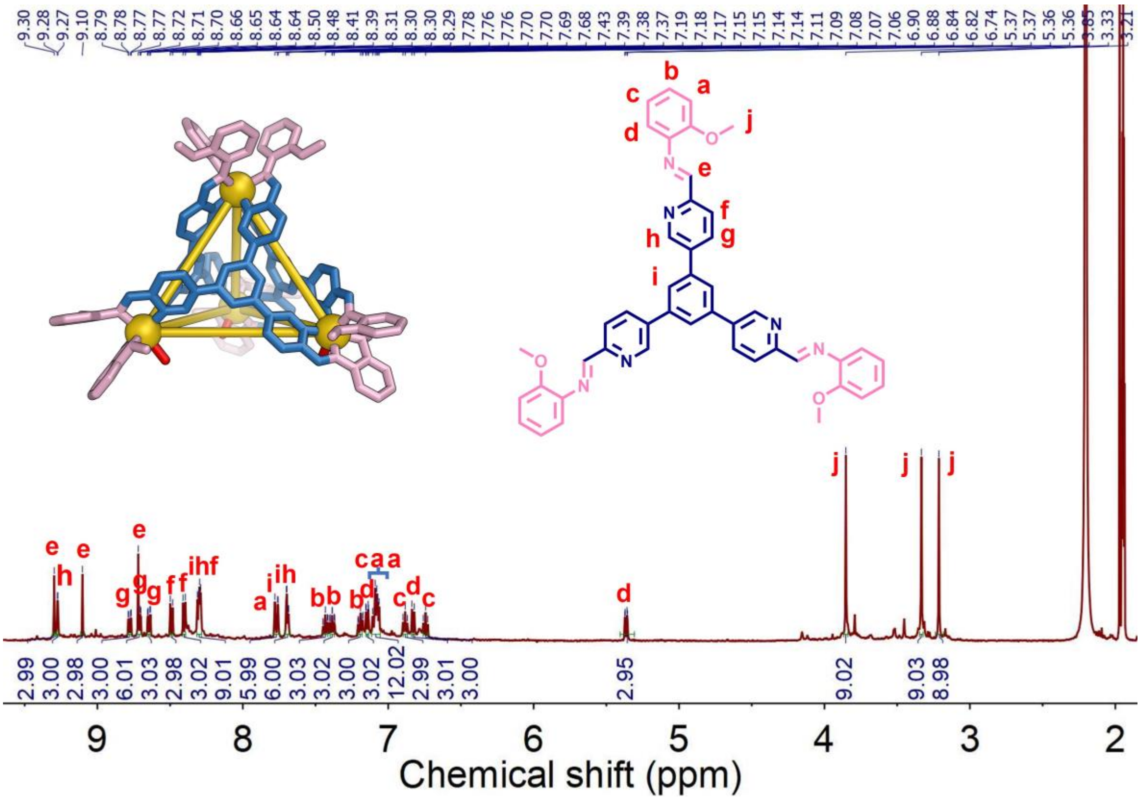

Supplementary Figure 3 | ^{1}H NMR spectrum (500 MHz, 298 K, CD_3CN) of **1**.

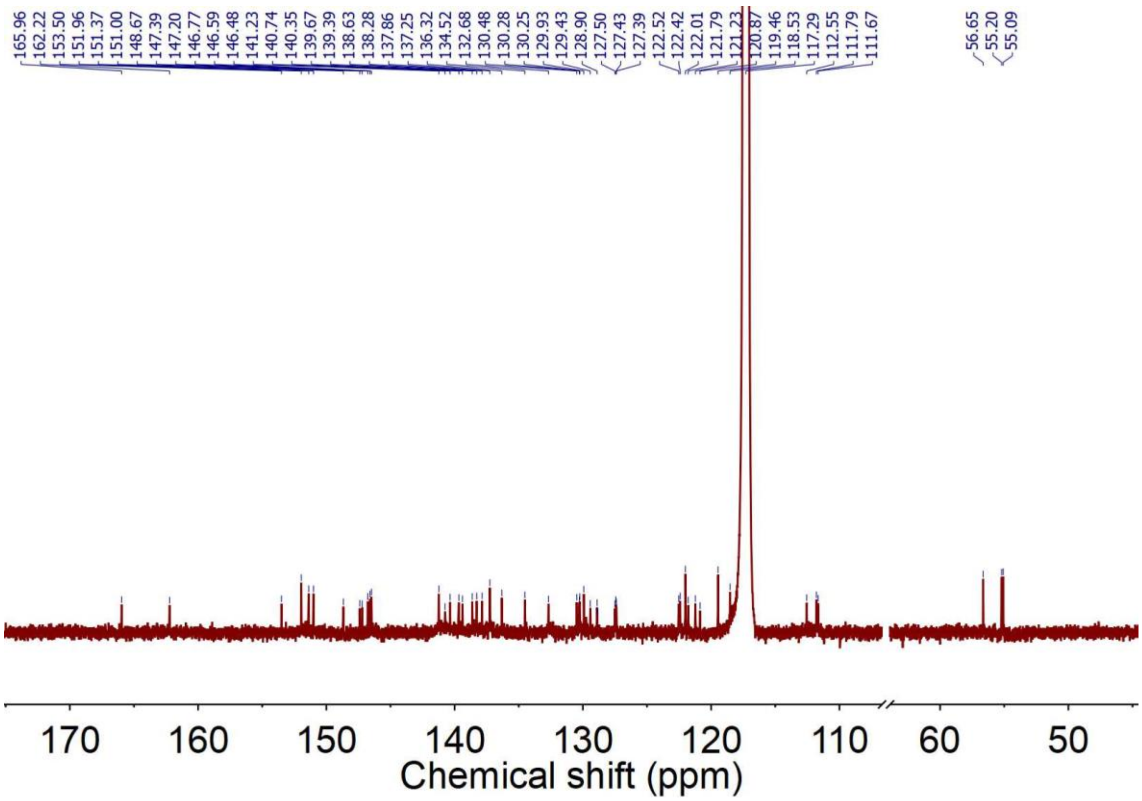

Supplementary Figure 4 | ^{13}C NMR spectrum (126 MHz, 298 K, CD_3CN) of **1**.

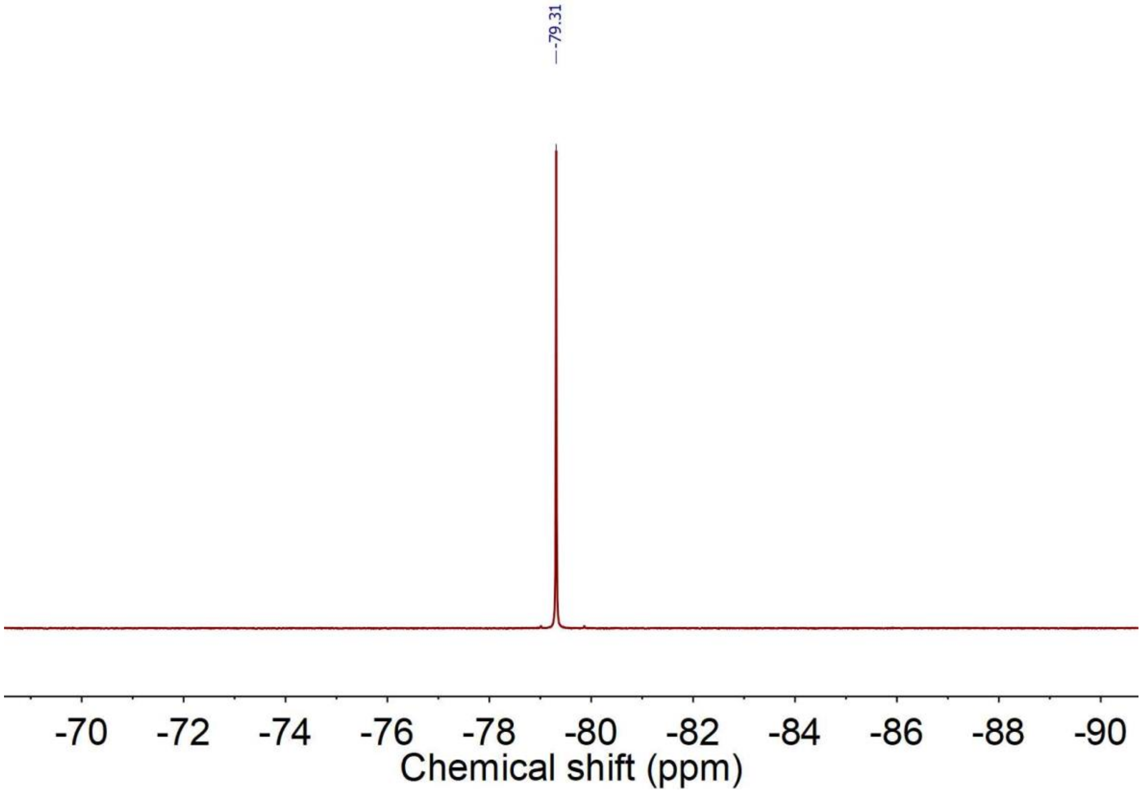

Supplementary Figure 5 | ^{19}F NMR spectrum (376 MHz, 298 K, CD_3CN) of **1.** δ –79.31 (s, CF_3).

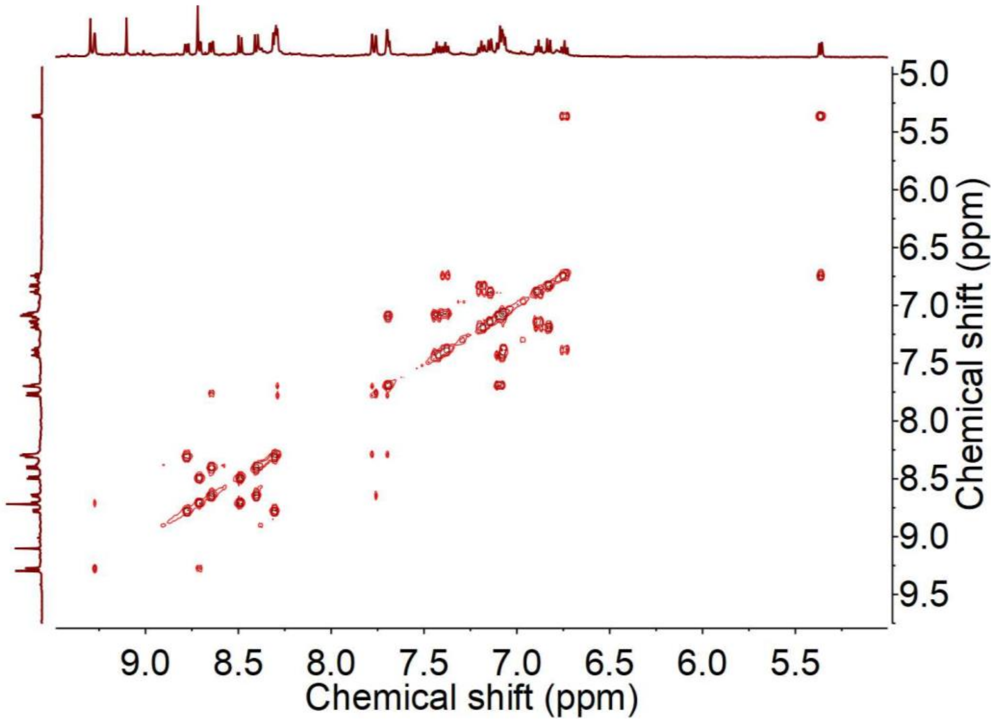

Supplementary Figure 6 | Aromatic region of the ^{1}H–^{1}H COSY spectrum (500 MHz, 298 K, CD_3CN) of **1**.

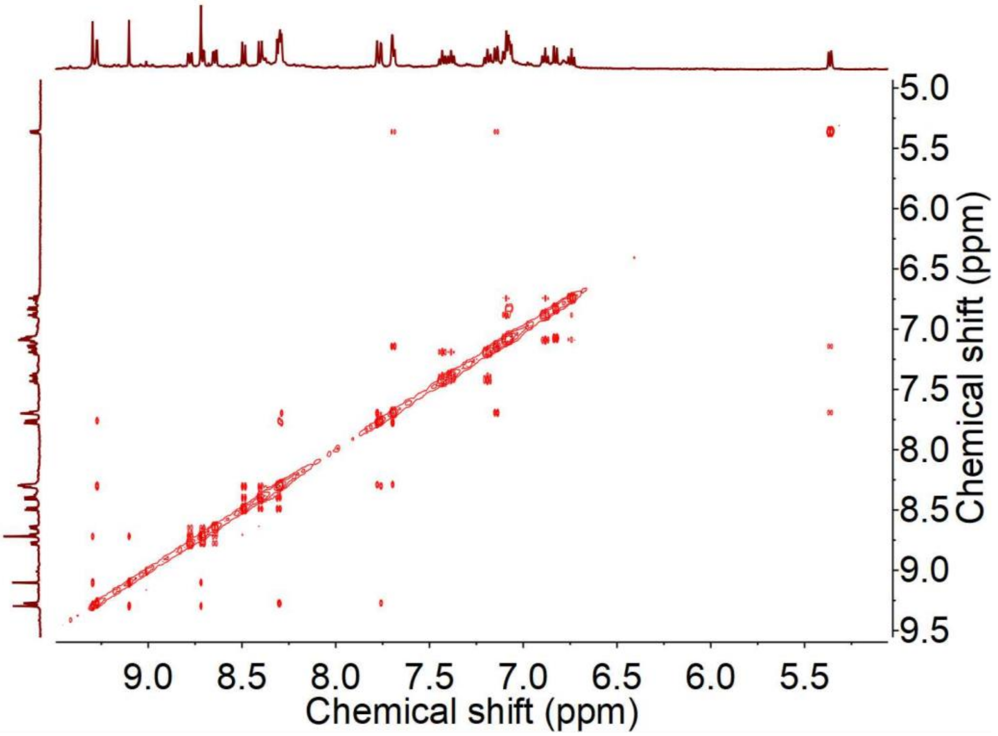

Supplementary Figure 7 | Aromatic region of the ^{1}H–^{1}H NOESY spectrum (500 MHz, 298 K, CD_3CN) of **1**.

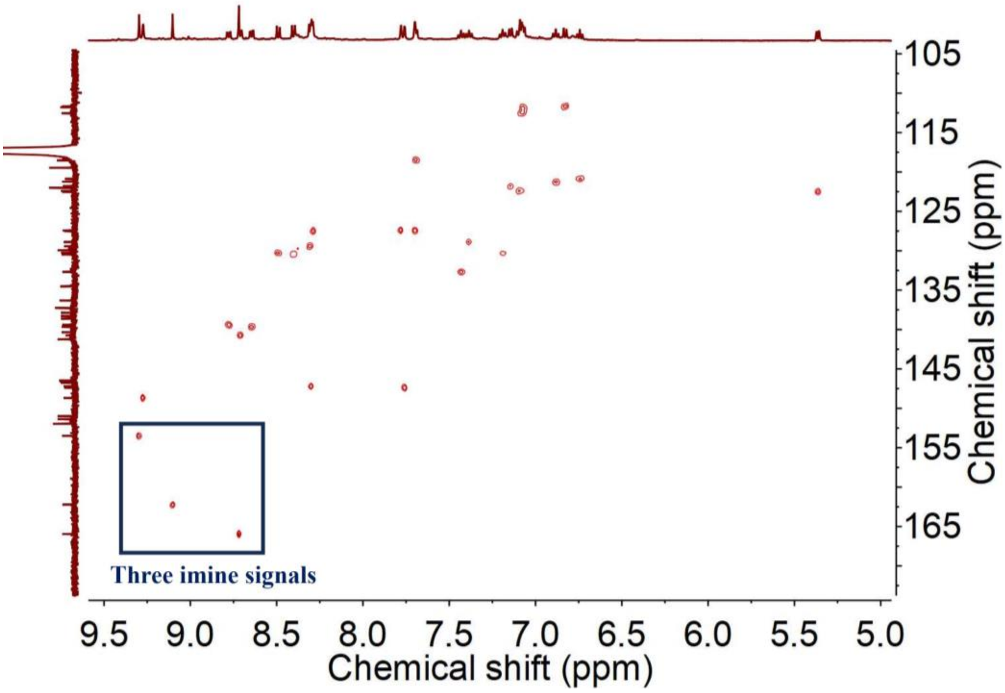

Supplementary Figure 8 | Aromatic region of the ^{1}H–^{13}C HSQC spectrum (500 MHz, 298 K, CD_3CN) of **1**.

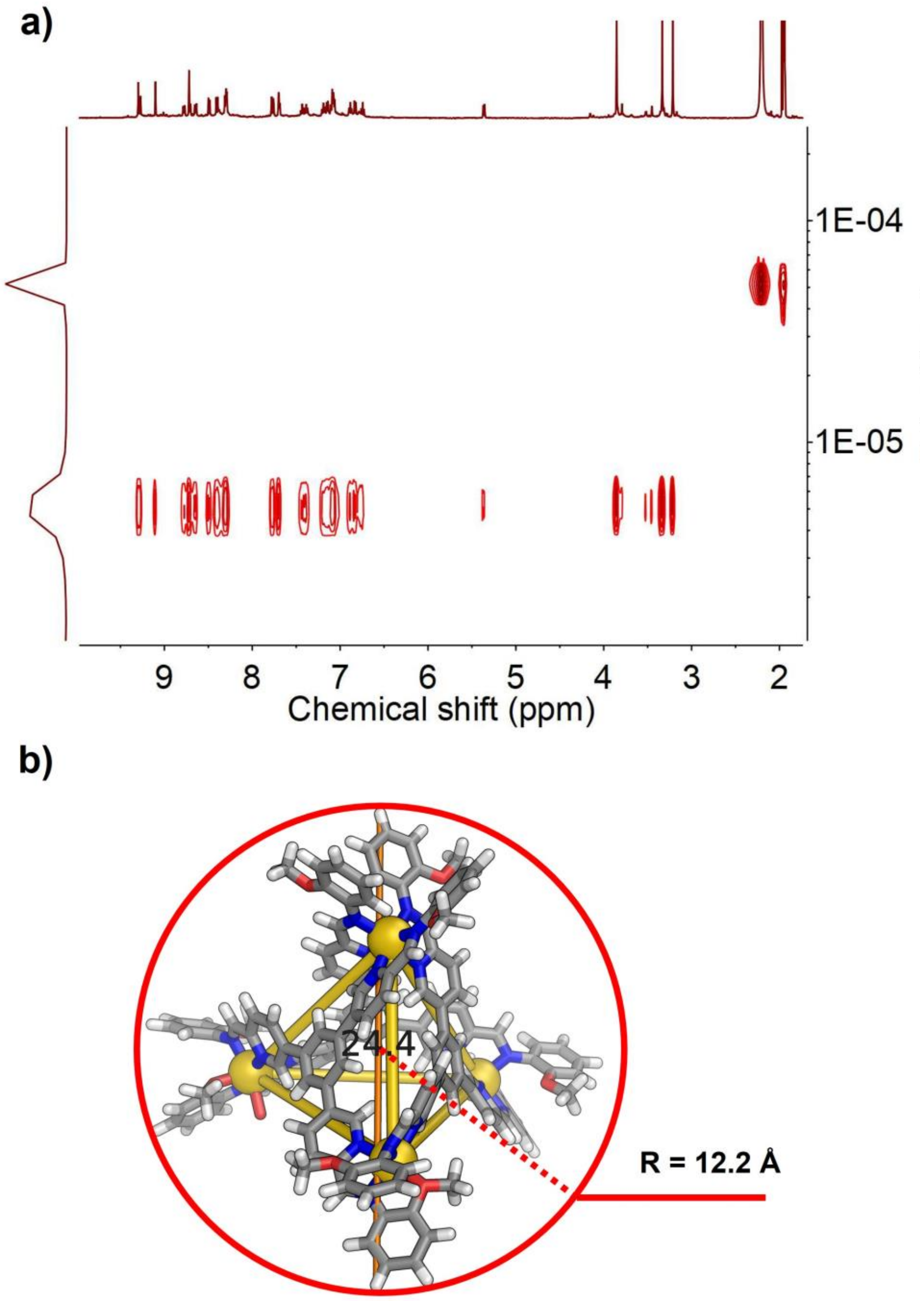

Supplementary Figure 9 | a) ^{1}H DOSY spectrum (400 MHz, 298 K, CD_3CN) of **1**. Diffusion coefficient: D = 5.11 $\times 10^{-10}$ m^2 s^{-1}, with radius of 12.8 Å; **b)** The radius of the modelled structure was calculated to be 12.2 Å, consistent with the DOSY result obtained for **1**.

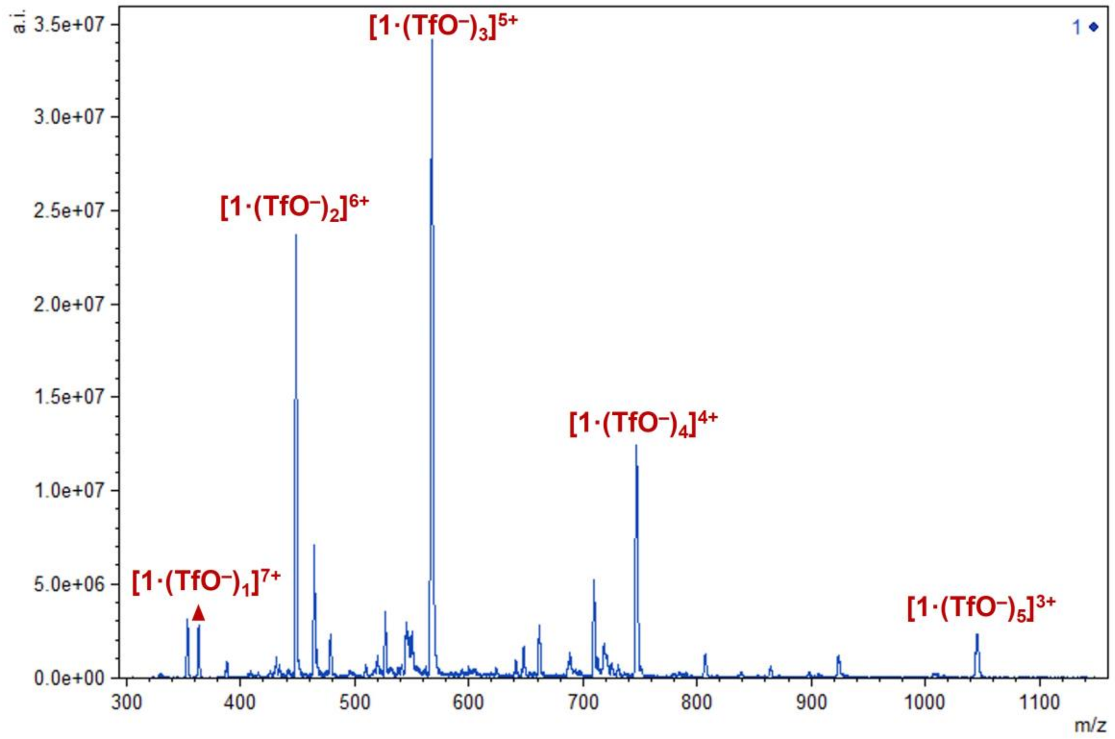

Supplementary Figure 10 | Low resolution ESI-MS of **1**. m/z = 362.5 [**1**·(TfO$^-$)$_1$]$^{7+}$, 447.8 [**1**·(TfO$^-$)$_2$]$^{6+}$, 567.0 [**1**·(TfO$^-$)$_3$]$^{5+}$, 746.2 [**1**·(TfO$^-$)$_4$]$^{4+}$,1045.0 [**1**·(TfO$^-$)$_5$]$^{3+}$. Calculated results: m/z = 362.4 [**1**·(TfO$^-$)$_1$]$^{7+}$, 447.7 [**1**·(TfO$^-$)$_2$]$^{6+}$, 567.0 [**1**·(TfO$^-$)$_3$]$^{5+}$, 746.1 [**1**·(TfO$^-$)$_4$]$^{4+}$,1044.5 [**1**·(TfO$^-$)$_5$]$^{3+}$.

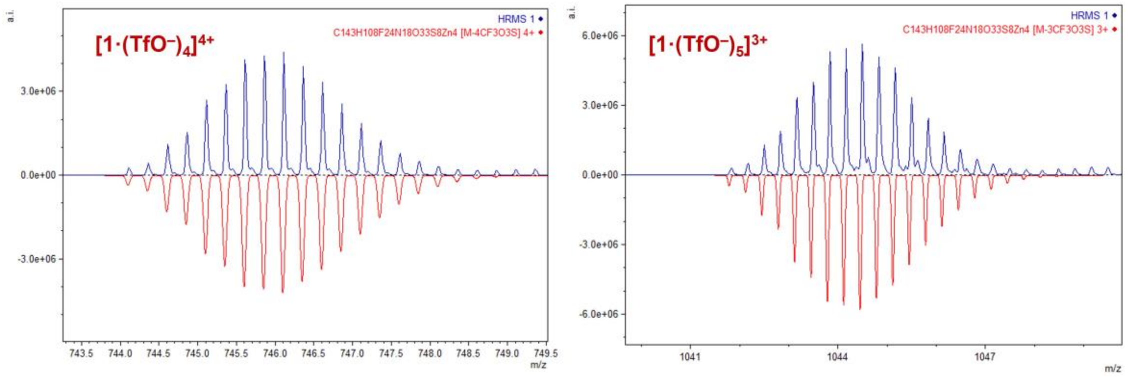

Supplementary Figure 11 | High resolution ESI-MS of **1**, showing the observed (top blue) and theoretical (bottom red) isotope patterns for the 4+, and 3+ peaks.

3.2 Self-assembly of **2**

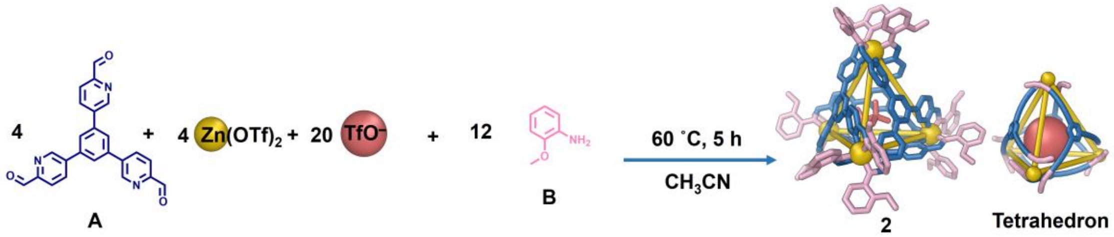

Supplementary Scheme 3 | Construction of **2**

A (5.0 mg, 1.0 equiv, 12.7 μmol), **B** (5.2 mg, 3.3 equiv, 41.9 μmol), potassium trifluoromethanesulfonate (12.0 mg, 5.0 equiv, 63.5 μmol) and zinc(II) trifluoromethanesulfonate (4.6 mg, 1.0 equiv, 12.7 μmol) were mixed in 2 mL acetonitrile. The reaction mixture was heated and stirred in a microwave reactor for 5 h at 60 ˚C. Then the mixture was concentrated to 0.5 mL and diethyl ether (14 mL) was added. The precipitate was collected by centrifugation and washed two times with excess diethyl ether. Flash size-exclusion chromatography (MeCN/DCM) was used to remove excess potassium trifluoromethanesulfonate, giving **2** in 90% yield (12.3 mg).

^{1}H NMR (500 MHz, CD_3CN) δ 8.69 (s, 12H), 8.65 (dd, *J* = 8.1, 2.1 Hz, 12H), 7.84 (d, *J* = 2.0 Hz, 12H), 7.41 (s, 12H), 7.35 (td, *J* = 8.3, 1.6 Hz, 12H), 7.03 (d, *J* = 7.7 Hz, 12H), 6.71 (t, *J* = 8.1 Hz, 12H), 5.31 (dd, *J* = 7.8, 1.5 Hz, 12H), 3.17 (s, 36H).
^{19}F NMR (376 MHz, CD_3CN) δ –79.34 (*exo*- TfO^-, s, CF_3), –79.45(*endo*- TfO^-, s, CF_3).
^{13}C NMR (126 MHz, CD_3CN) δ 166.0, 151.0, 146.6, 146.3, 140.5, 138.3, 137.4, 136.7, 130.5, 128.7, 127.4, 122.5, 121.1 (q, $^{1}J_{CF}$ = 320.9 Hz, TfO^-), 120.8, 117.3, 111.7, 55.3.

ESI-MS: *m*/*z*: 463.8 [**2**·$(TfO^-)_1]^{7+}$, 566.1 [**2**·$(TfO^-)_2]^{6+}$, 709.1 [**2**·$(TfO^-)_3]^{5+}$, 923.7 [**2**·$(TfO^-)_4]^{4+}$.

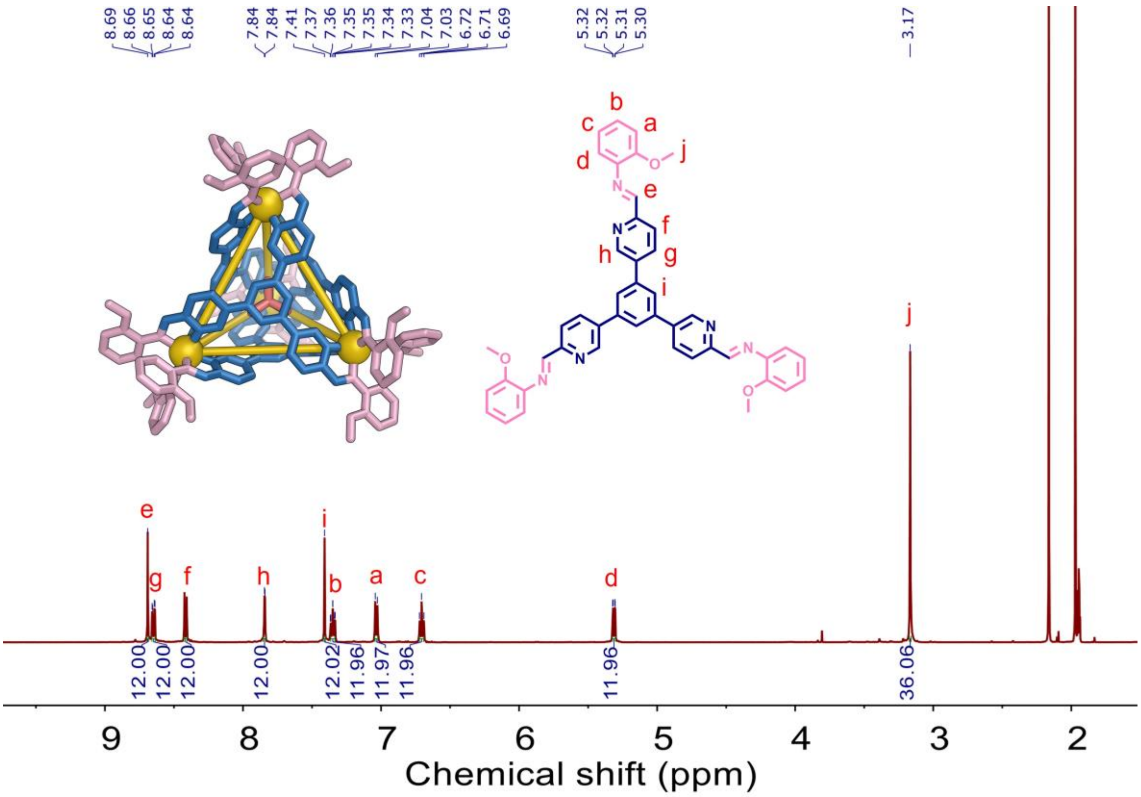

Supplementary Figure 12 | ^{1}H NMR spectrum (500 MHz, 298 K, CD_3CN) of **2**

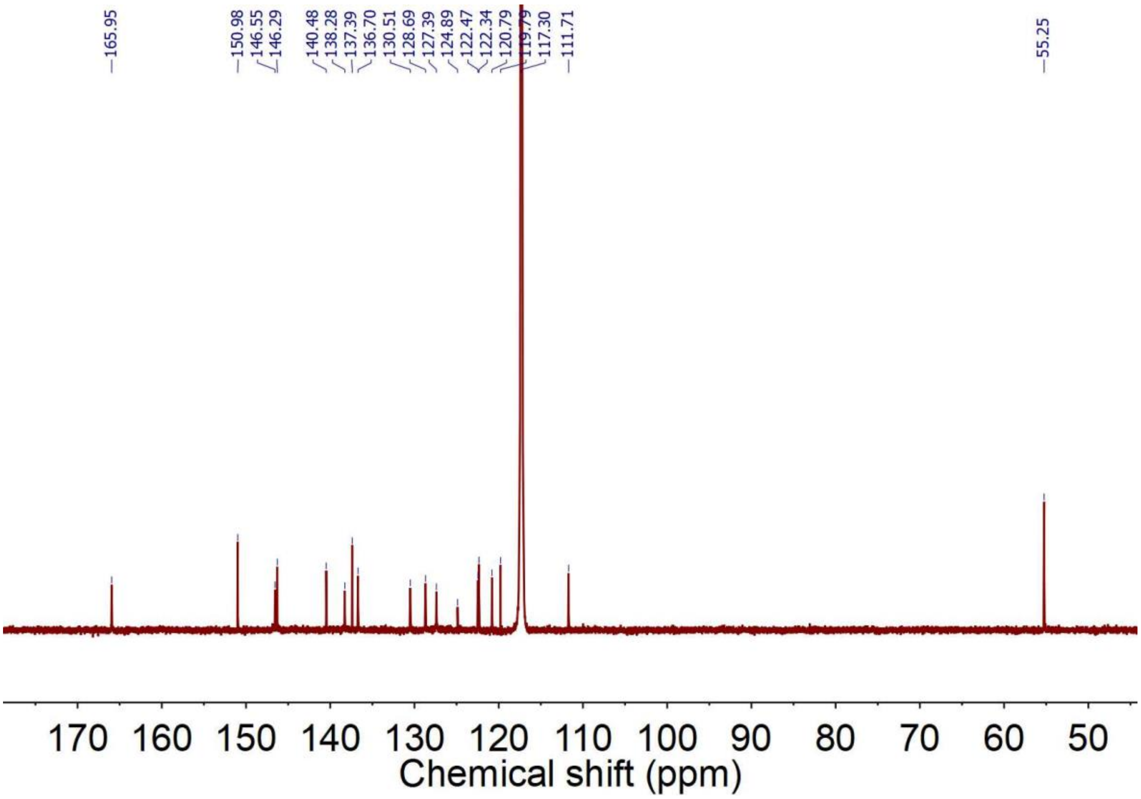

Supplementary Figure 13 | ^{13}C NMR spectrum (126 MHz, 298 K, CD_3CN) of **2**

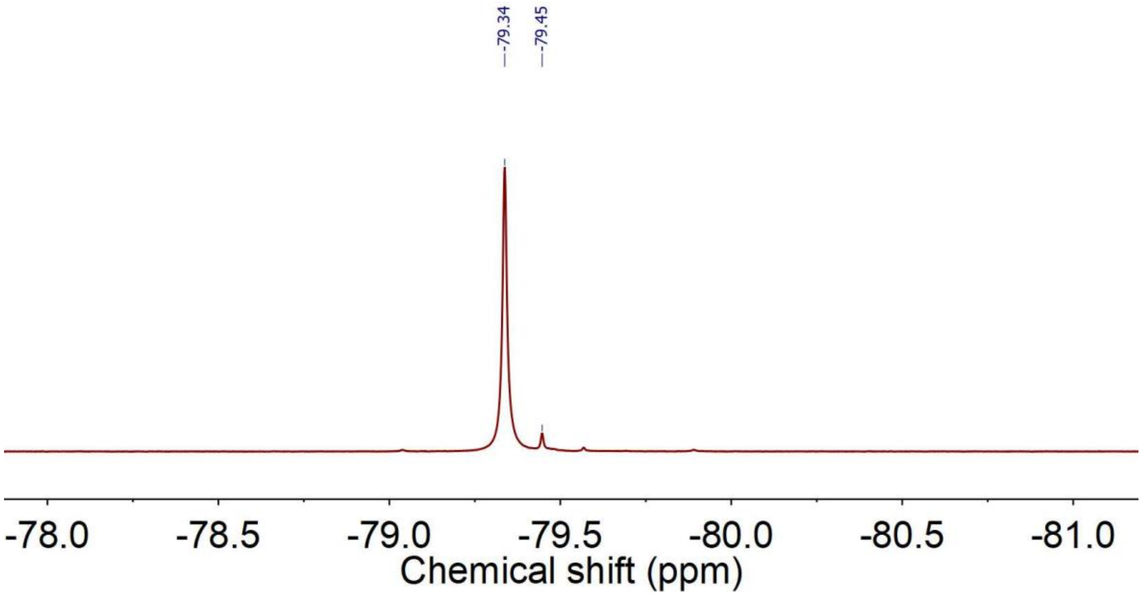

Supplementary Figure 14 | ^{19}F NMR spectrum (376 MHz, 298 K, CD_3CN) of **2**.

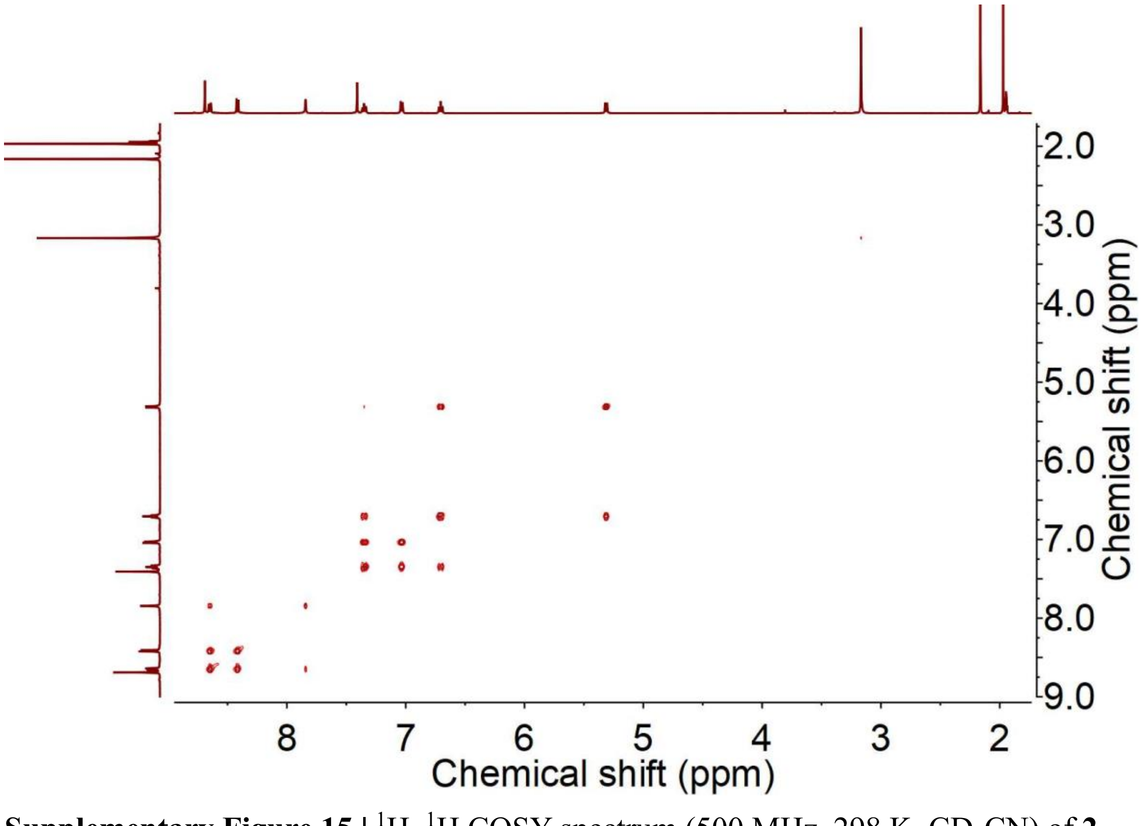

Supplementary Figure 15 | ^{1}H–^{1}H COSY spectrum (500 MHz, 298 K, CD_3CN) of **2**.

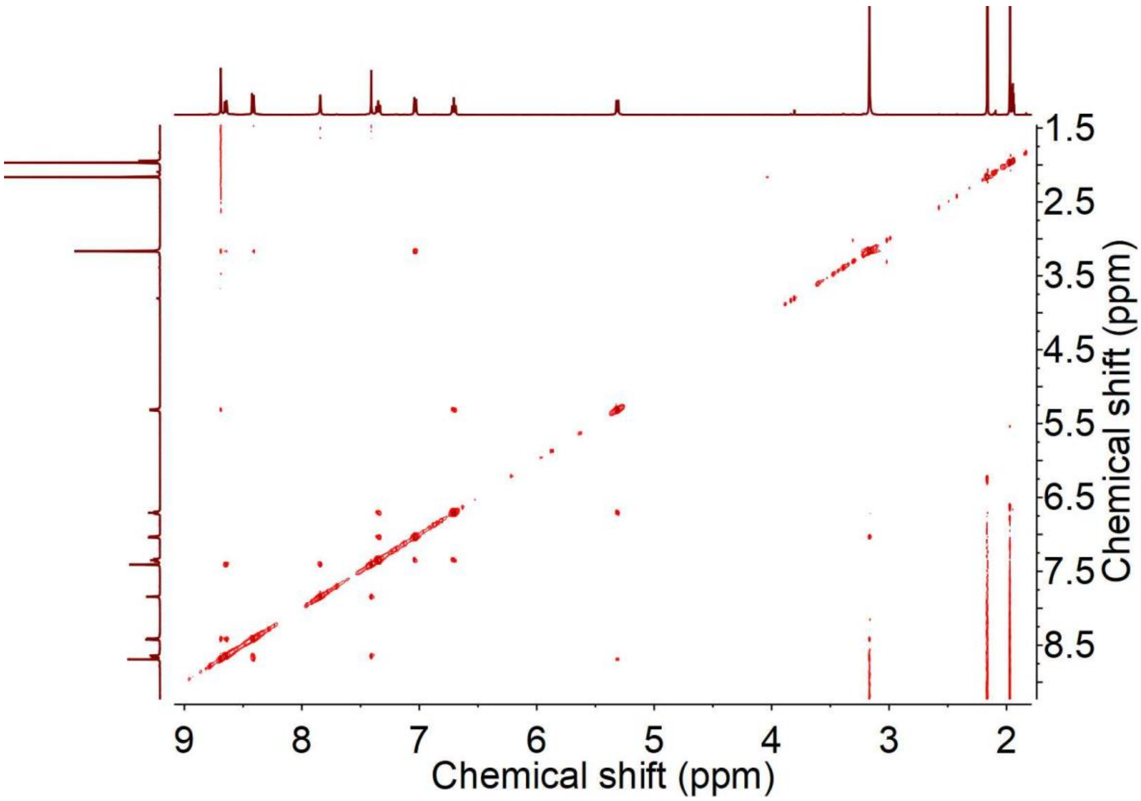

Supplementary Figure 16 | ^{1}H–^{1}H NOESY spectrum (500 MHz, 298 K, CD_3CN) of **2**.

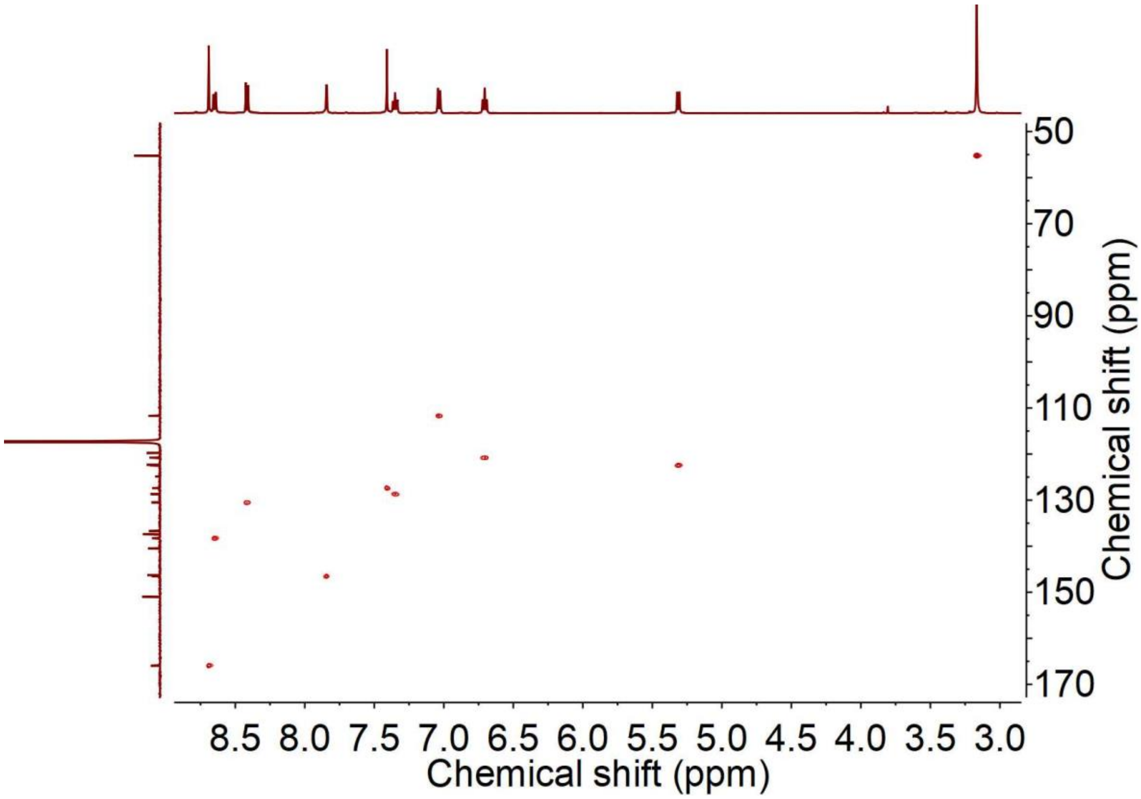

Supplementary Figure 17 | ^{1}H–^{13}C HSQC spectrum (500 MHz, 298 K, CD_3CN) of **2**.

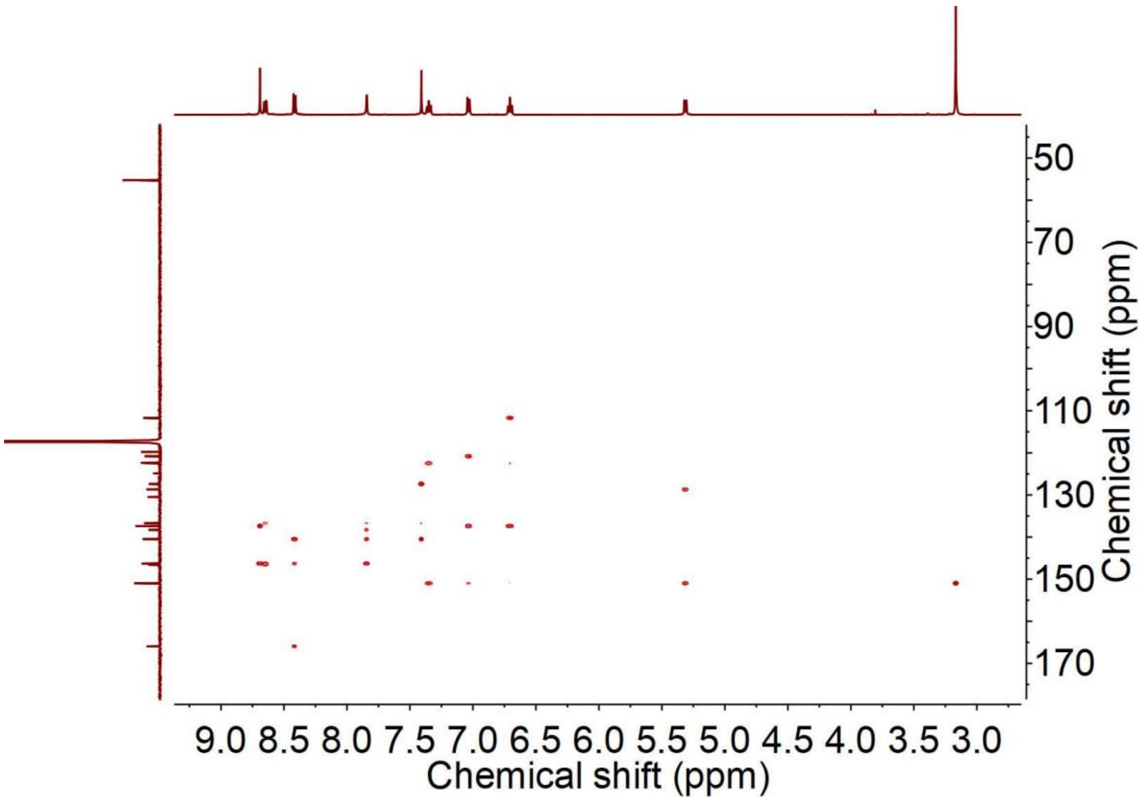

Supplementary Figure 18 | ^{1}H–^{13}C HMBC spectrum (500 MHz, 298 K, CD_3CN) of **2**.

a)

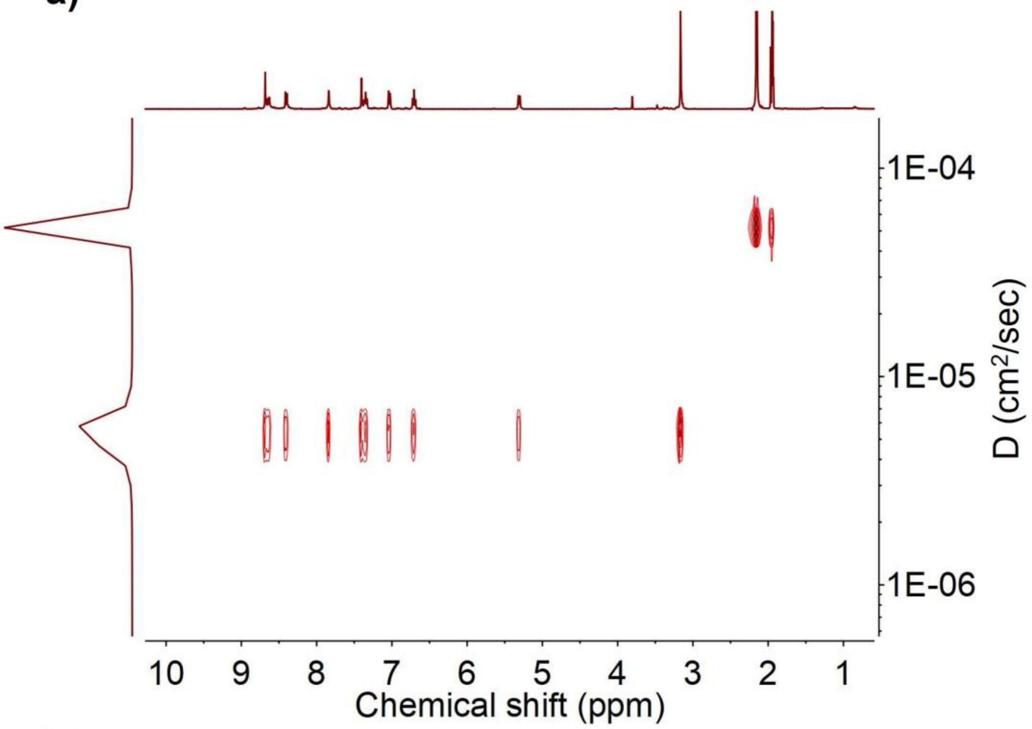

b)

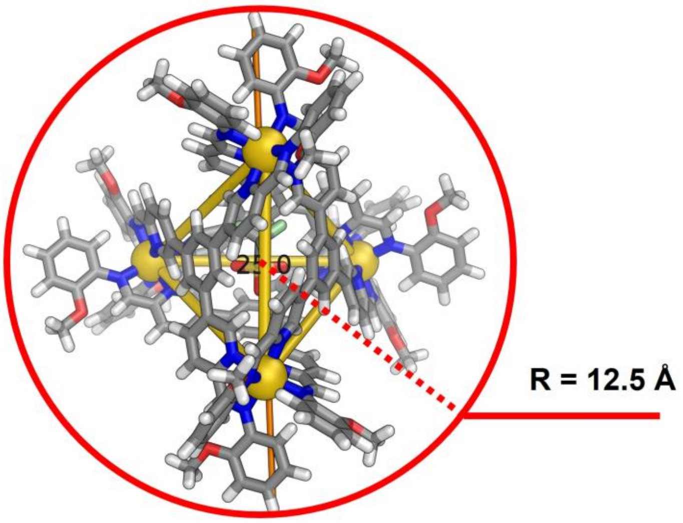

Supplementary Figure 19 | a) ^{1}H DOSY spectrum (400 MHz, 298 K, CD_3CN) of **2**. Diffusion coefficient: D = 5.48×10^{-10} m^2 s^{-1}, with radius of = 11.9 Å; **b)** The radius of the modelled structure was calculated to be 12.5 Å, consistent with the DOSY result obtained for **2**.

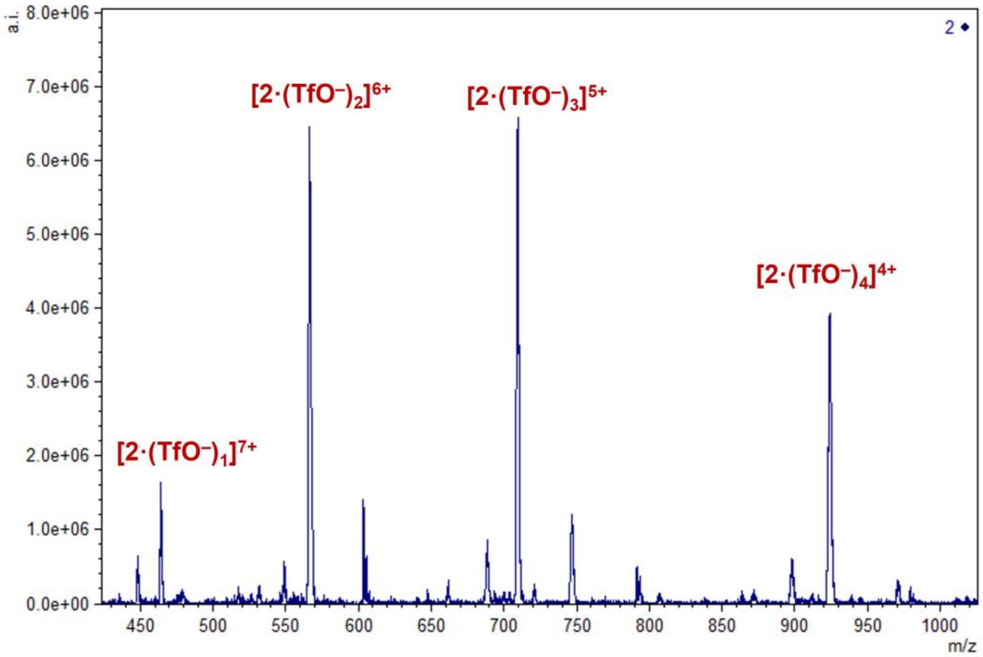

Supplementary Figure 20 | Low-resolution ESI-MS spectrum of **2**. *m/z* = 463.8 [**2**·$(TfO^{-})_1$]$^{7+}$, 566.1 [**2**·$(TfO^{-})_2$]$^{6+}$, 709.1 [**2**·$(TfO^{-})_3$]$^{5+}$, 923.7 [**2**·$(TfO^{-})_4$]$^{4+}$. Calculated results: *m/z* = 463.7 [**2**·$(TfO^{-})_1$]$^{7+}$, 565.8 [**2**·$(TfO^{-})_2$]$^{6+}$, 708.8 [**2**·$(TfO^{-})_3$]$^{5+}$, 923.3 [**2**·$(TfO^{-})_4$]$^{4+}$.

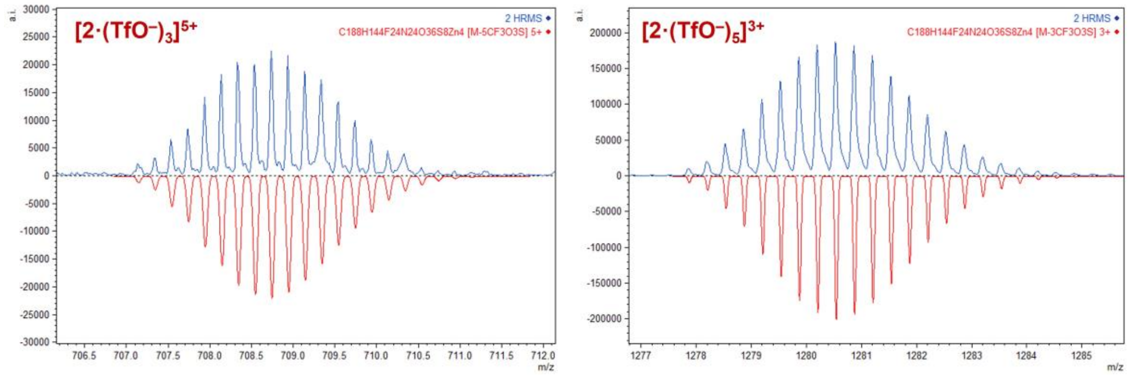

Supplementary Figure 21 | High-resolution ESI-mass spectrometry analysis of **2** showing the observed (top blue) and theoretical (bottom red) isotope patterns for the 5+ and 3+ peaks.

3.2 Self-assembly of **3**

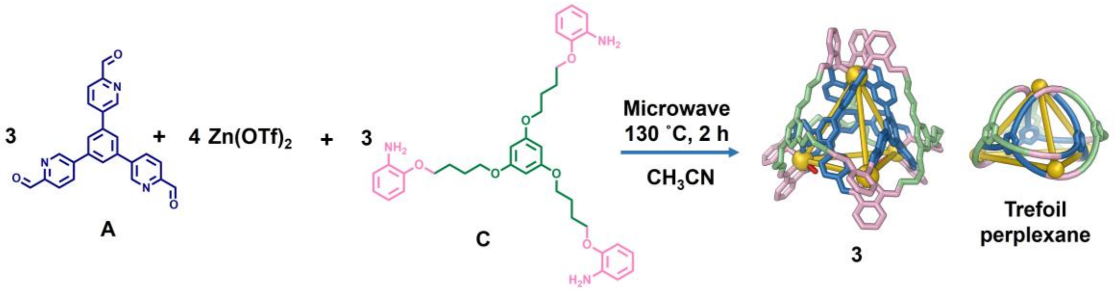

Supplementary Scheme 4 | Construction of **3**

A (5.0 mg, 1.0 equiv, 12.7 μmol), **C** (8.6 mg, 1.1 equiv, 14.0 μmol) and zinc(II) trifluoromethanesulfonate (6.2 mg, 1.3 equiv, 16.9 μmol) were mixed in 2 mL acetonitrile. The reaction mixture was heated and stirred in a microwave reactor for 2 h at 130 ˚C. Then the mixture was concentrated to 0.5 mL and diethyl ether (14 mL) was added. The precipitate was collected by centrifugation and washed two times with excess diethyl ether to give **3** in 91% yield (16.7 mg).

^{1}H NMR (500 MHz, CD_3CN) δ 9.25 (s, 3H), 9.24 (s, 3H), 9.08 (s, 3H), 8.86 (dd, *J* = 8.1, 2.1 Hz, 3H), 8.80 – 8.73 (m, 9H), 8.60 (d, *J* = 8.0 Hz, 3H), 8.52 (dd, *J* = 8.3, 2.3 Hz, 3H), 8.29 (d, *J* = 8.2 Hz, 3H), 8.00 (s, 3H), 7.72 – 7.66 (m, 6H), 7.52 (s, 3H), 7.49 (s, 3H), 7.45 (t, *J* = 7.8 Hz, 6H), 7.35 (t, *J* = 7.8 Hz, 6H), 7.20 – 7.12 (m, 9H), 7.10 – 7.03 (m, 6H), 7.00 (d, *J* = 8.4 Hz, 3H), 6.88 – 6.81 (m, 6H), 6.69 (d, *J* = 8.4 Hz, 3H), 5.90 (s, 3H), 5.28 (dd, *J* = 7.8, 1.7 Hz, 3H), 5.23 (d, *J* = 2.1 Hz, 6H), 4.29 – 4.21 (br, 3H), 4.20 – 4.13 (br, 3H), 3.88 – 3.81 (br, 12H), 3.68 (q, *J* = 7.2 Hz, 3H), 3.51 – 3.40 (m, 9H), 3.28 (q, *J* = 7.2 Hz, 3H), 3.01 (q, *J* = 7.2 Hz, 3H), 2.14 – 2.12 (br, 3H), 1.82 – 1.80 (br, 3H), 1.74 – 1.70 (br, 3H), 1.60 – 1.57 (m, 6H), 1.51 – 1.48 (br, 3H), 1.32 – 1.27 (m, 9H), 0.98 – 0.86 (m, 6H), 0.81 – 0.76 (br, 3H).

^{13}C NMR (126 MHz, CD_3CN) δ 167.1, 162.4, 160.9, 160.0, 159.5, 153.0, 151.7, 151.5, 150.6, 146.7, 146.7, 146.7, 146.0, 145.7, 141.0, 140.5, 140.3, 139.4, 138.2, 137.3, 136.8, 136.6, 136.4, 136.0, 135.2, 132.6, 131.5, 130.7, 130.0, 129.9, 129.6, 129.0, 125.5, 125.4, 124.8, 123.1, 122.3, 121.9, 120.6 (m, TfO^-), 121.0, 120.8, 118.4, 113.7, 113.3, 112.8, 96.8, 91.3, 90.1, 71.5, 69.1, 68.7, 68.5, 68.1, 67.5, 29.9, 27.3, 25.7, 25.6, 24.8, 24.4.

^{19}F NMR (376 MHz, CD_3CN) δ –79.50 (s, CF_3).

ESI-MS (CH_3CN) for 3: *m/z* = 570.8 $[\mathbf{3}\cdot(TfO^-)_2]^{6+}$, 714.8 $[\mathbf{3}\cdot(TfO^-)_3]^{5+}$, 930.7 $[\mathbf{3}\cdot(TfO^-)_4]^{4+}$, 1290.6 $[\mathbf{3}\cdot(TfO^-)_5]^{3+}$, 2010.3 $[\mathbf{3}\cdot(TfO^-)_6]^{2+}$.

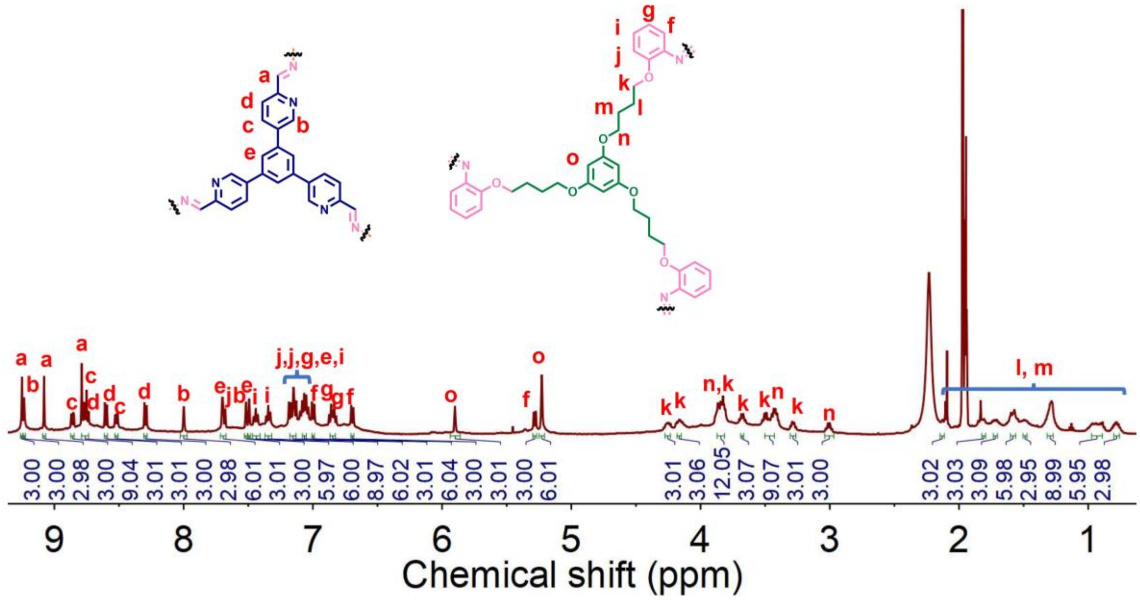

Supplementary Figure 22 | ^{1}H NMR spectrum (500 MHz, 298 K, CD_3CN) of **3**.

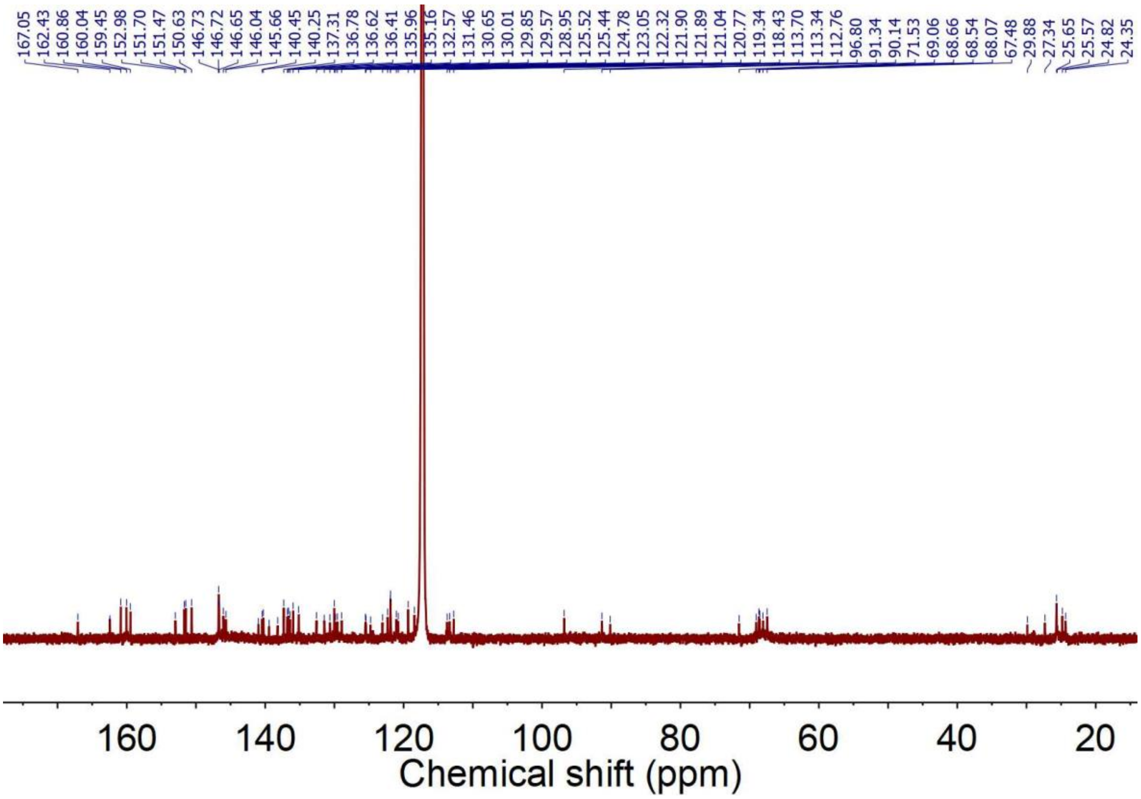

Supplementary Figure 23 | ^{13}C NMR spectrum (500 MHz, 298 K, CD_3CN) of **3**.

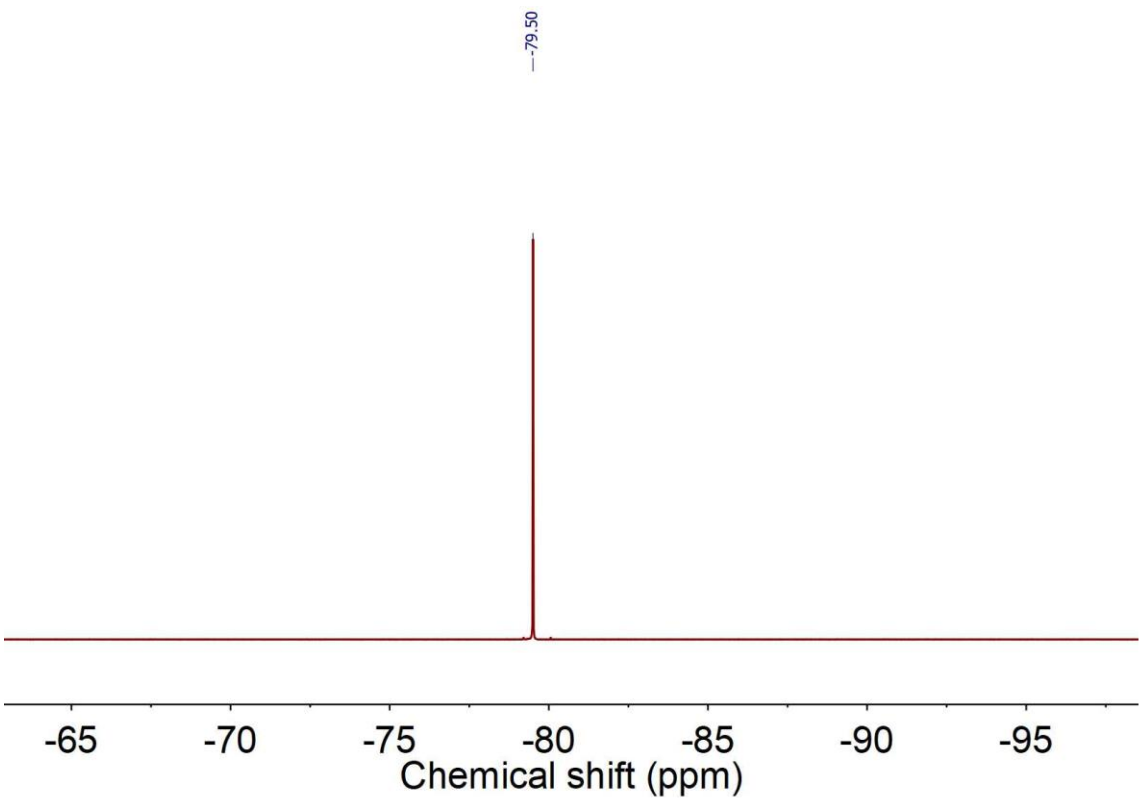

Supplementary Figure 24 | ^{19}F NMR spectrum (376 MHz, 298 K, CD_3CN) of **3.** δ –79.50 (s, CF_3).

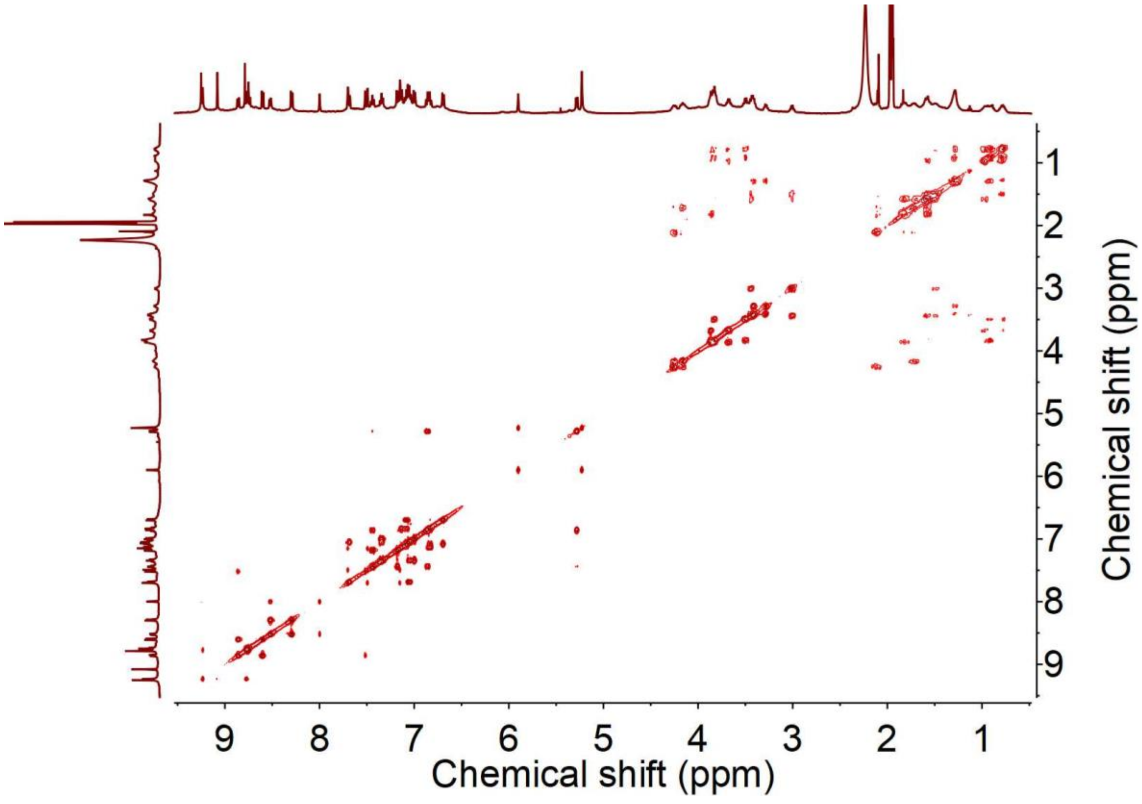

Supplementary Figure 25 | ^{1}H–^{1}H COSY spectrum (500 MHz, 298 K, CD_3CN) of **3**.

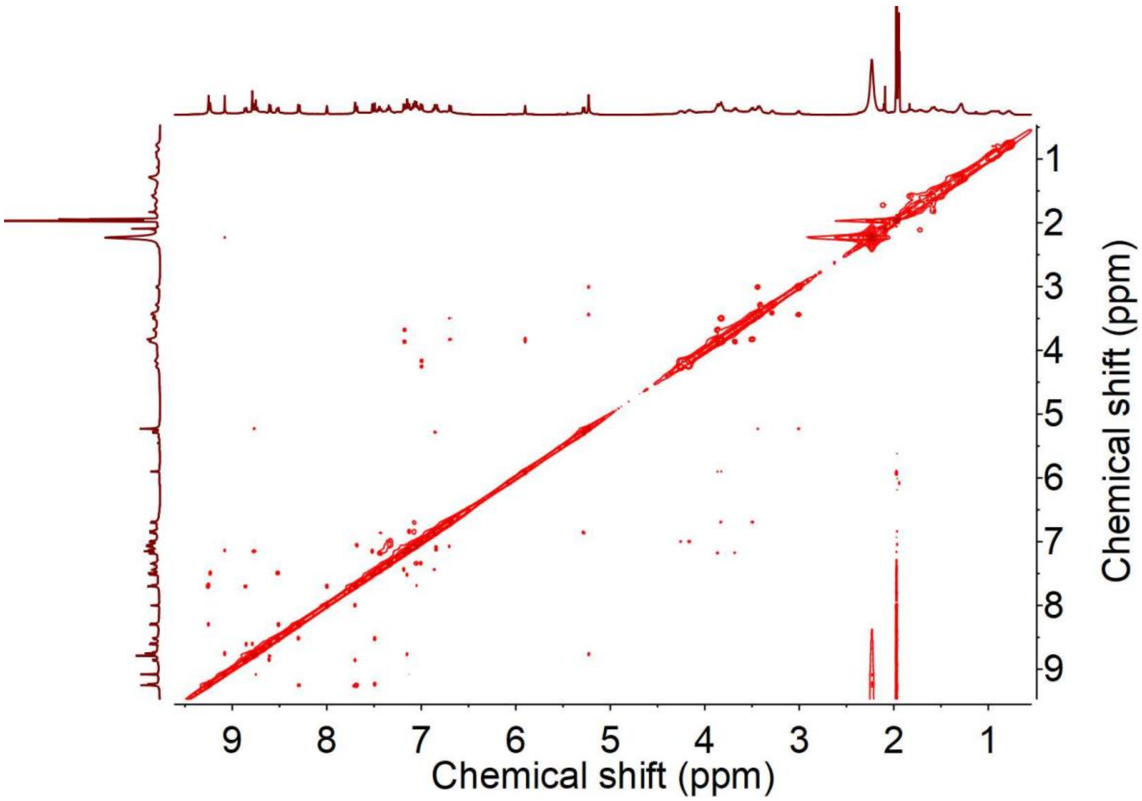

Supplementary Figure 26 | ^{1}H–^{1}H NOESY spectrum (500 MHz, 298 K, CD_3CN) of **3**.

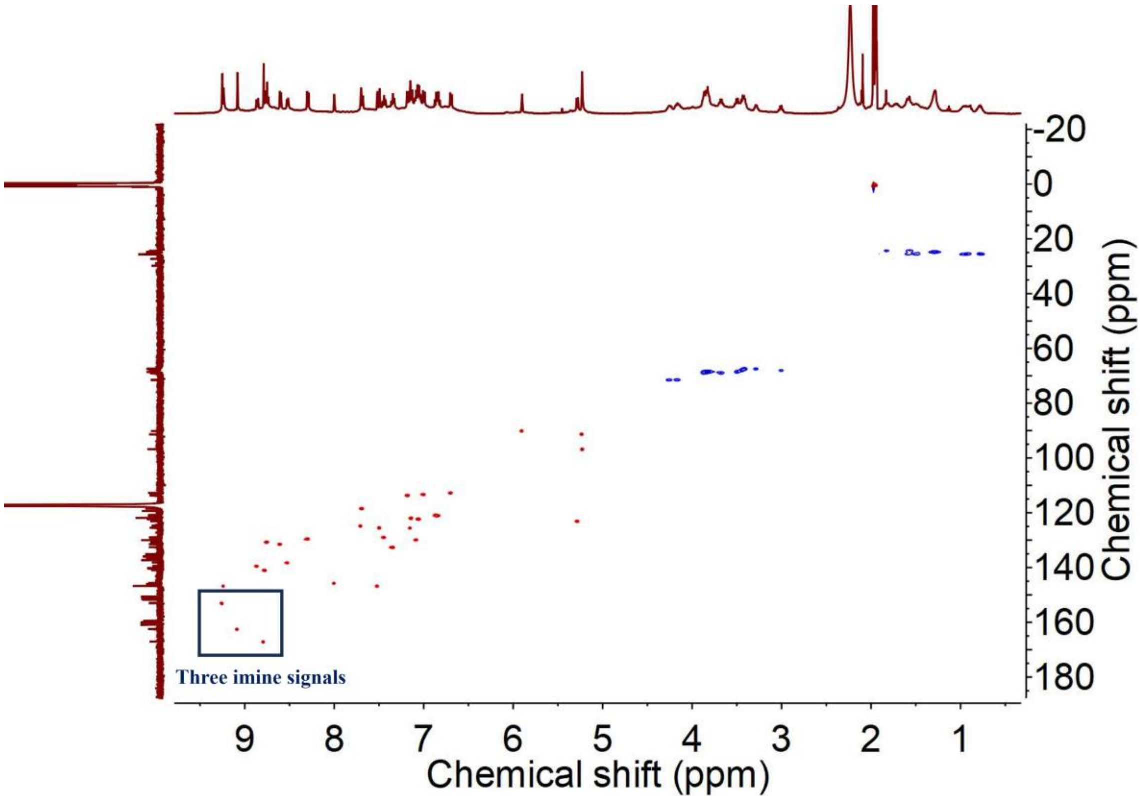

Supplementary Figure 27 | ^{1}H–^{13}C HSQC spectrum (500 MHz, 298 K, CD_3CN) of **3**.

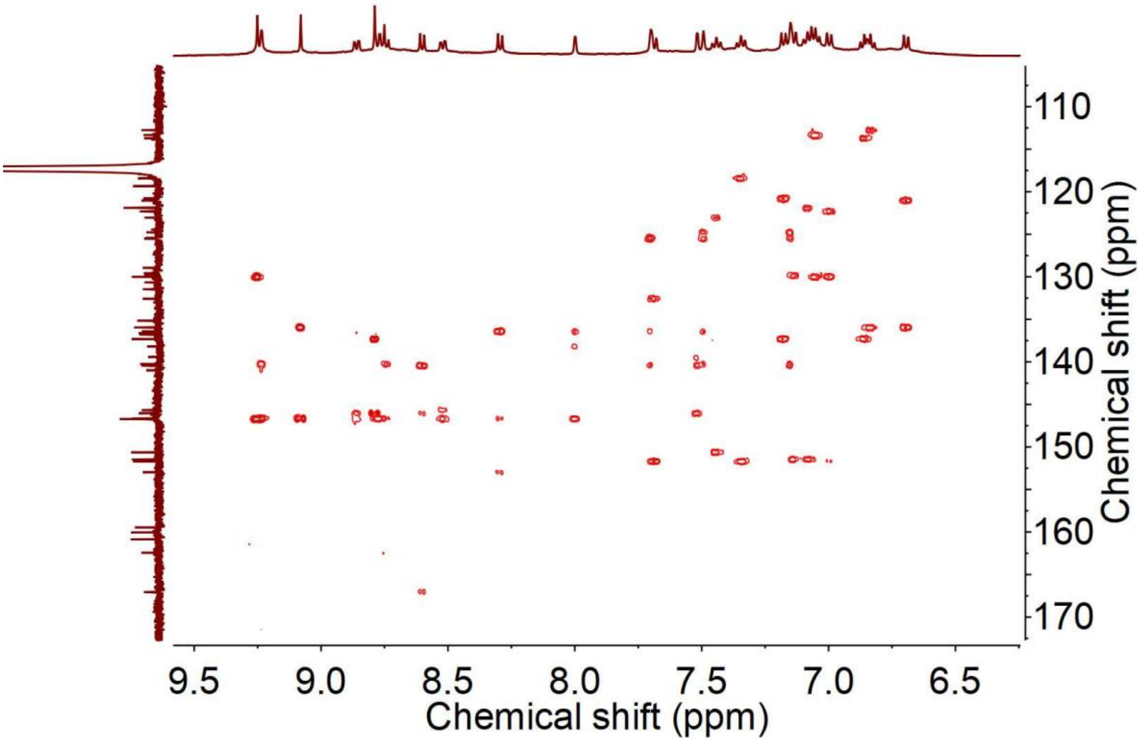

Supplementary Figure 28 | Aromatic region of the ^{1}H–^{13}C HMBC spectrum (500 MHz, 298 K, CD_3CN) of **3**.

a)

b)

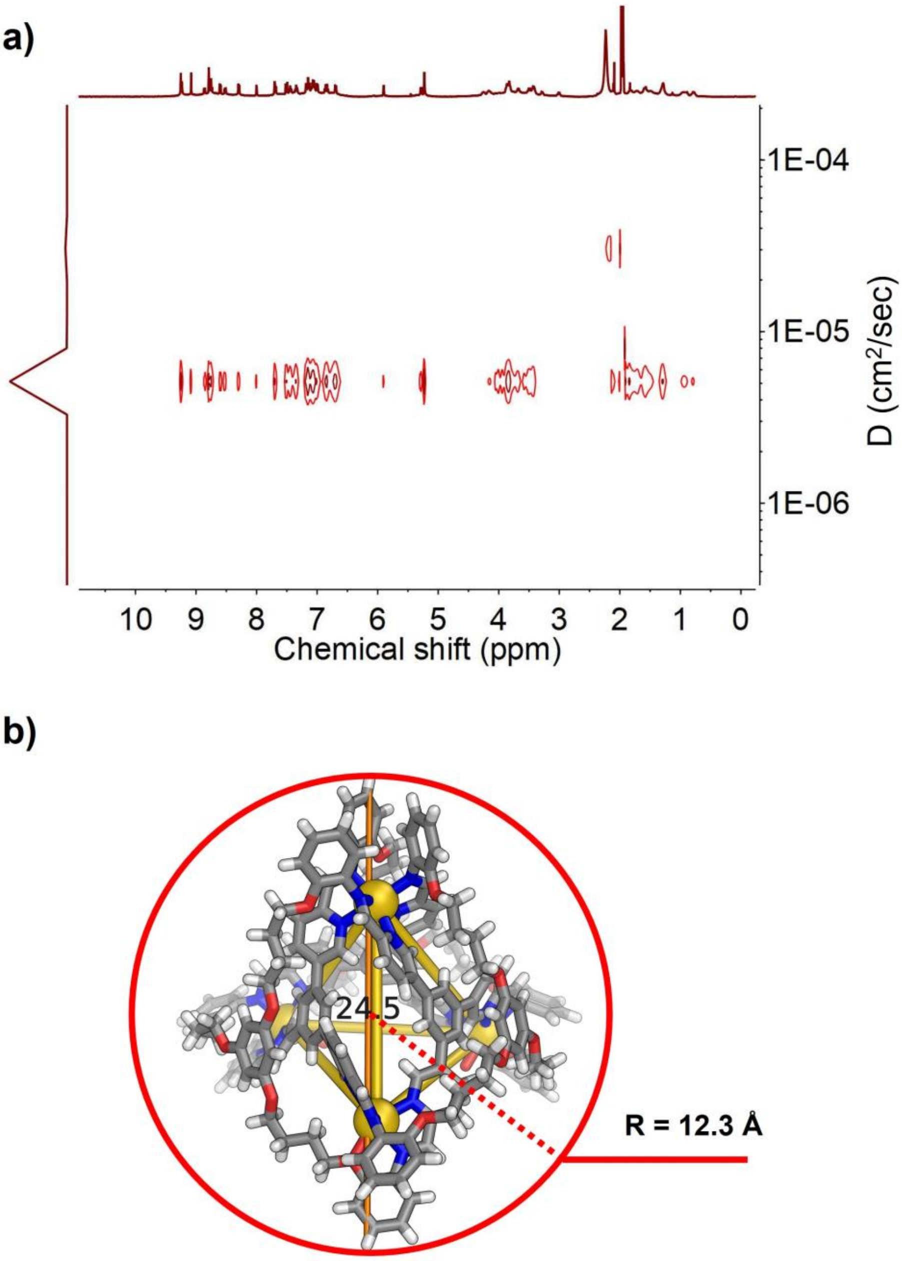

Supplementary Figure 29 | ^{1}H DOSY spectrum (400 MHz, 298 K, CD_3CN) of **3**. Diffusion coefficient: D = 5.12 × 10^{-10} m^2 s^{-1}, with diameter of = 12.8 Å; **b)** The radius of the crystal structure was measured to be 12.3 Å, consistent with the DOSY result obtained for **3**.

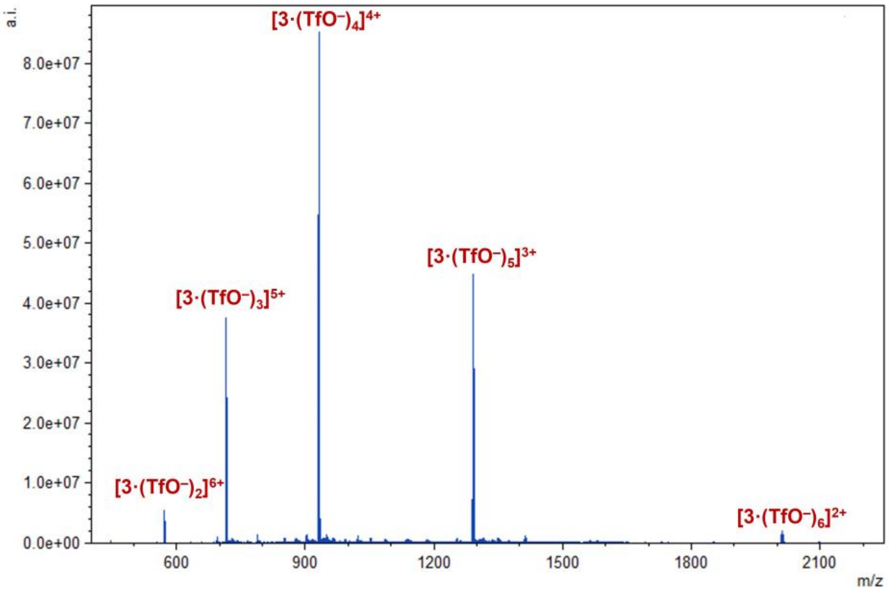

Supplementary Figure 30 | Low-resolution ESI-MS spectrum of **3**. *m/z* = 570.8 $[\mathbf{3}\cdot(\mathrm{TfO}^-)_2]^{6+}$, 714.8 $[\mathbf{3}\cdot(\mathrm{TfO}^-)_3]^{5+}$, 930.7 $[\mathbf{3}\cdot(\mathrm{TfO}^-)_4]^{4+}$, 1290.6 $[\mathbf{3}\cdot(\mathrm{TfO}^-)_5]^{3+}$, 2010.3 $[\mathbf{3}\cdot(\mathrm{TfO}^-)_6]^{2+}$. Calculated peaks: m/z = 570.8 $[\mathbf{3}\cdot(\mathrm{TfO}^-)_2]^{6+}$, 714.8 $[\mathbf{3}\cdot(\mathrm{TfO}^-)_3]^{5+}$, 930.8 $[\mathbf{3}\cdot(\mathrm{TfO}^-)_4]^{4+}$, 1290.8 $[\mathbf{3}\cdot(\mathrm{TfO}^-)_5]^{3+}$, 2010.7 $[\mathbf{3}\cdot(\mathrm{TfO}^-)_6]^{2+}$.

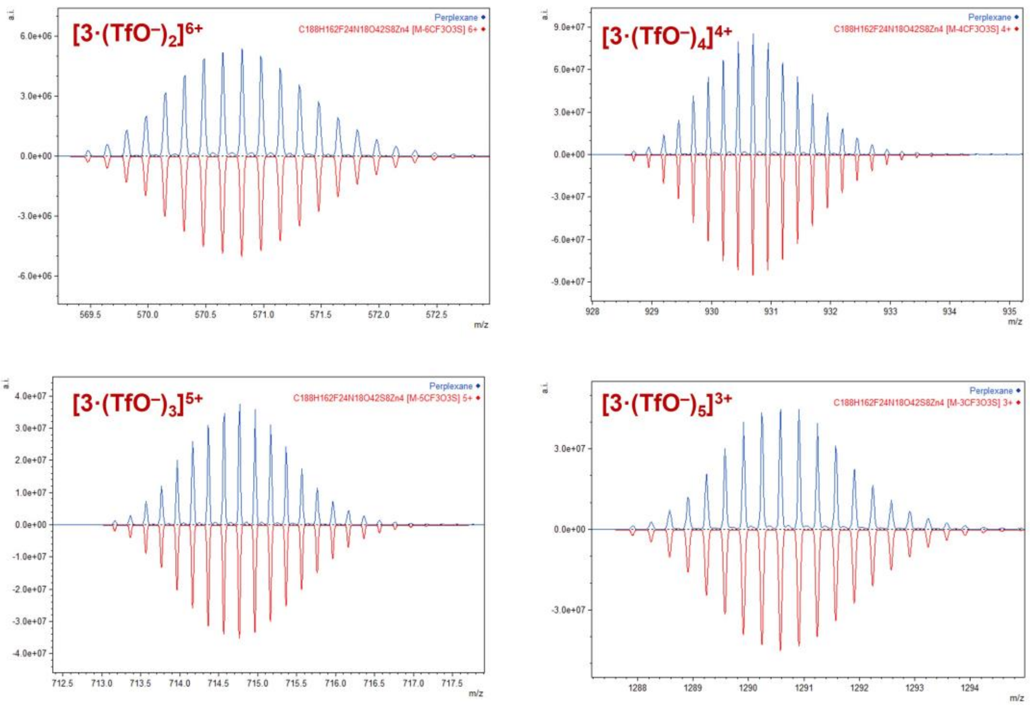

Supplementary Figure 31 | High-resolution ESI-mass spectrometry analysis of **3** showing the observed (top blue) and theoretical (bottom red) isotope patterns for the 6+, 5+, 4+ and 3+ peaks.

3.4 Self-assembly of **4**

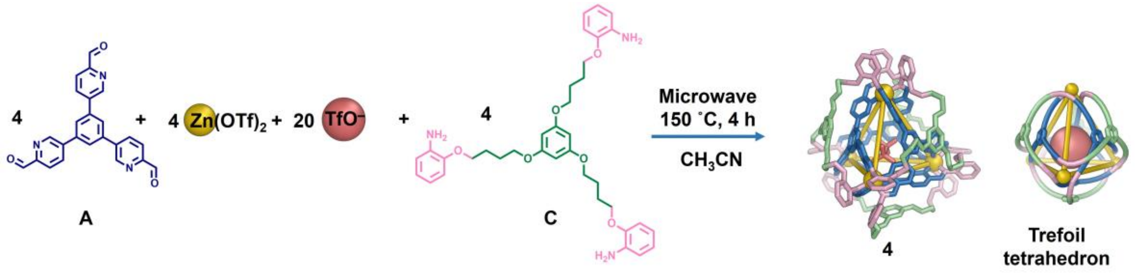

Supplementary Scheme 5 | Construction of **4**

A (5.0 mg, 1.0 equiv, 12.7 μmol), **C** (8.6 mg, 1.1 equiv, 14.0 μmol), potassium trifluoromethanesulfonate (12.0 mg, 5.0 equiv, 63.5 μmol) and zinc(II) trifluoromethanesulfonate (4.6 mg, 1.0 equiv, 12.7 μmol) were mixed in 2 mL acetonitrile. The reaction mixture was heated and stirred in a microwave reactor for 4 h at 150 ˚C. Then the mixture was concentrated to 0.5 mL and diethyl ether (14 mL) was added. The precipitate was collected by centrifugation and washed two times with excess diethyl ether. A flash size-exclusion chromatography (MeCN/DCM) was used to remove excess potassium trifluoromethanesulfonate, giving **4** in 79% yield (13.2 mg).

^{1}H NMR (500 MHz, CD_3CN) δ 8.77 (s, 12H), 8.56 (d, *J* = 8.1 Hz, 12H), 8.44 (dd, *J* = 8.1, 2.1 Hz, 12H), 7.60 (d, *J* = 2.0 Hz, 12H), 7.33 (td, *J* = 9.6, 8.2, 1.7 Hz, 12H), 7.12 (d, *J* = 8.4 Hz, 12H), 6.97 (s, 12H), 6.58 (t, *J* = 7.7 Hz, 12H), 5.53 (s, 12H), 5.40 (dd, *J* = 7.8, 1.6 Hz, 12H), 3.95 – 3.89 (br, 12H), 3.64 – 3.53 (m, 24H), 3.48 – 3.39 (br, 12H), 1.48 – 1.25 (m, 48H).

^{13}C NMR (126 MHz, CD_3CN) δ 166.1, 160.1, 150.0, 146.4, 146.0, 141.0, 138.7, 138.3, 136.0, 131.2, 128.8, 126.7, 123.4, 121.9, 121.2, 120.1, 117.3, 113.7, 93.1, 67.4, 24.3, 24.0.
^{19}F NMR (376 MHz, CD_3CN) δ –78.57 (*endo*- TfO^-, s, CF_3), –79.28 (*exo*- TfO^-, s, CF_3).
ESI-MS: *m/z*: = 604.3 $[\mathbf{4}\cdot(TfO^-)_1]^{7+}$, 730.1 $[\mathbf{4}\cdot(TfO^-)_2]^{6+}$, 905.7 $[\mathbf{4}\cdot(TfO^-)_3]^{5+}$, 1169.4 $[\mathbf{4}\cdot(TfO^-)_4]^{4+}$, 1609.2 $[\mathbf{4}\cdot(TfO^-)_5]^{3+}$. Calculated results: m/z = 604.4 $[\mathbf{4}\cdot(TfO^-)_1]^{7+}$, 730.0 $[\mathbf{4}\cdot(TfO^-)_2]^{6+}$, 905.8 $[\mathbf{4}\cdot(TfO^-)_3]^{5+}$, 1169.6 $[\mathbf{4}\cdot(TfO^-)_4]^{4+}$, 1609.1 $[\mathbf{4}\cdot(TfO^-)_5]^{3+}$.

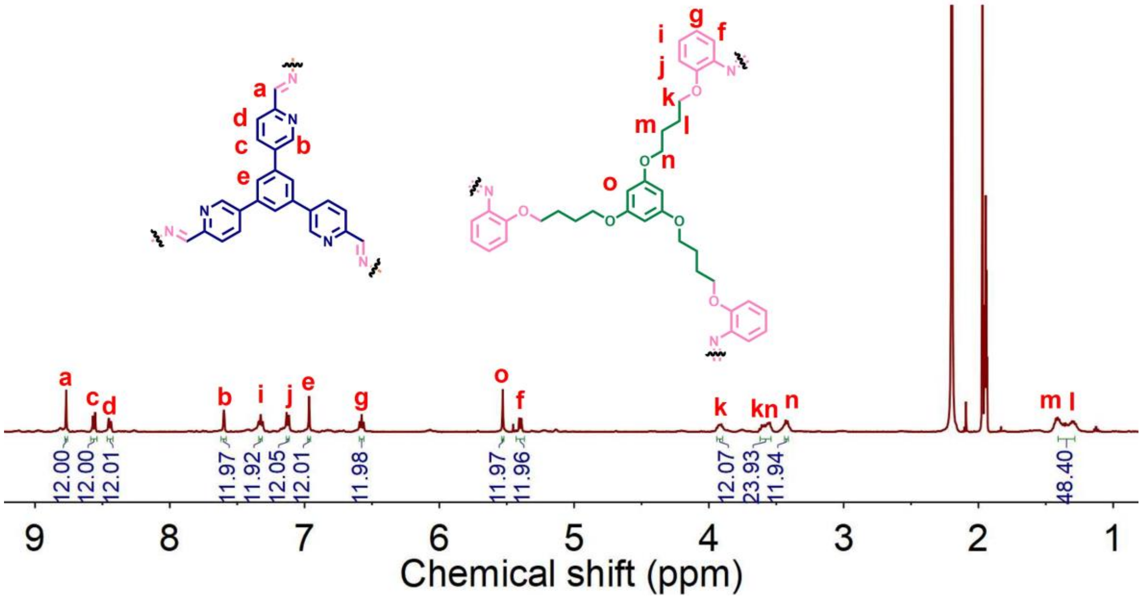

Supplementary Figure 32 | ^{1}H NMR spectrum (500 MHz, 298 K, CD_3CN) of **4**

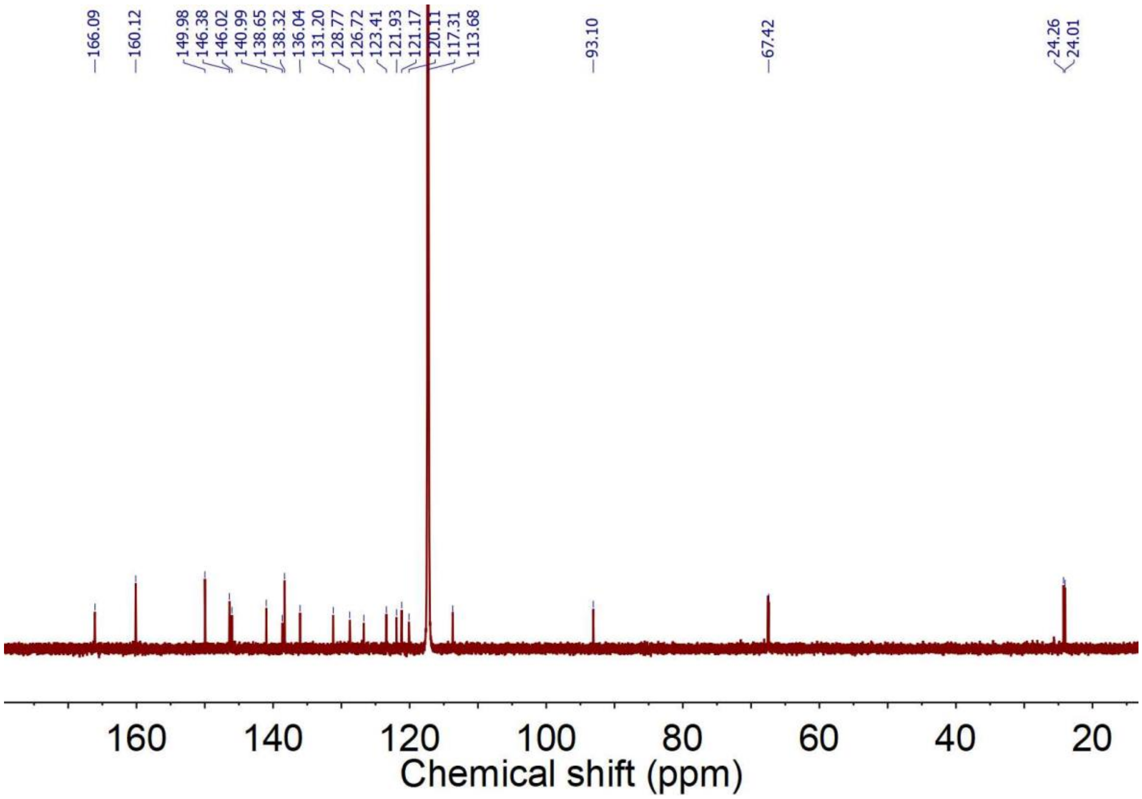

Supplementary Figure 33 | ^{13}C NMR spectrum (500 MHz, 298 K, CD_3CN) of **4**.

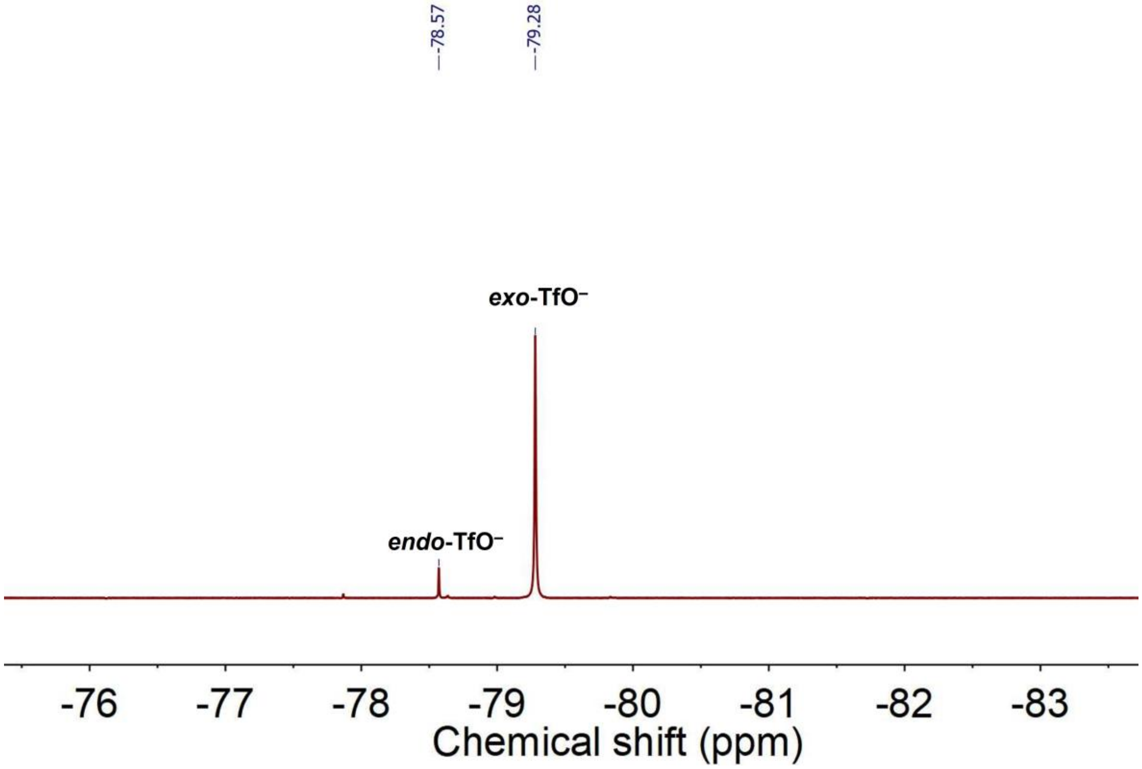

Supplementary Figure 34 | ^{19}F NMR spectrum (376 MHz, 298 K, CD_3CN) of **4**.

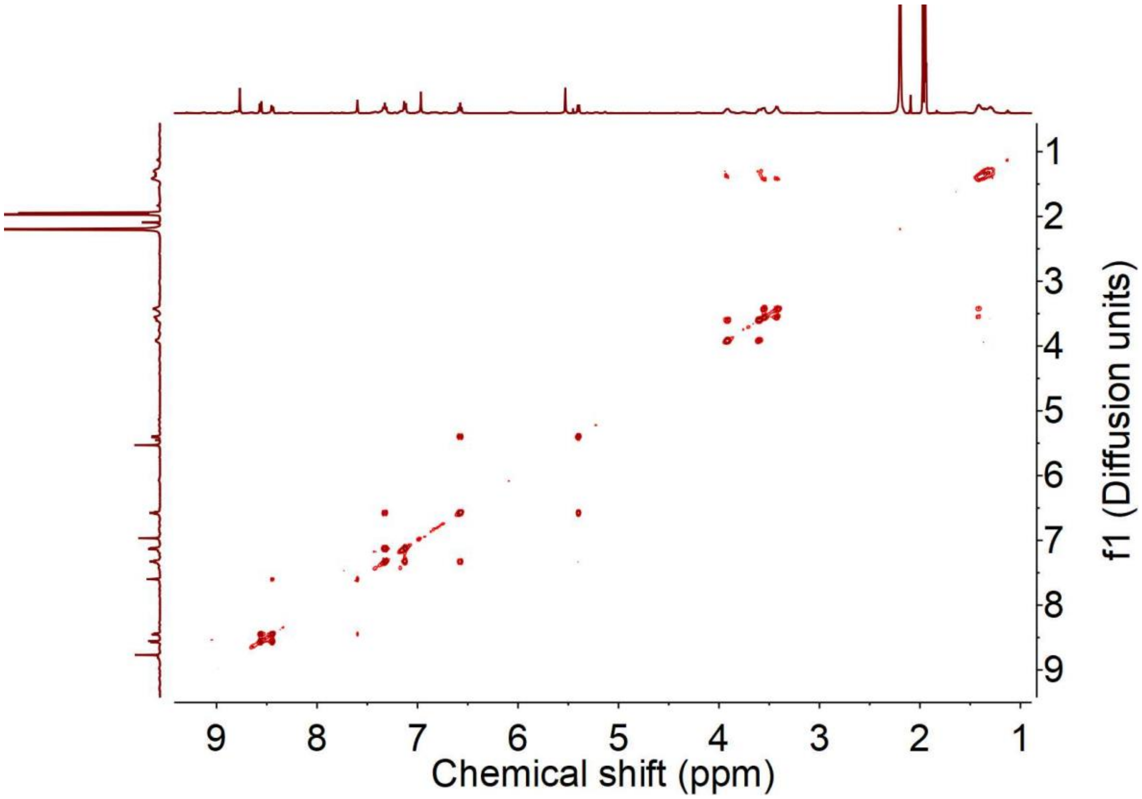

Supplementary Figure 35 | ^{1}H–^{1}H COSY spectrum (500 MHz, 298 K, CD_3CN) of **4**.

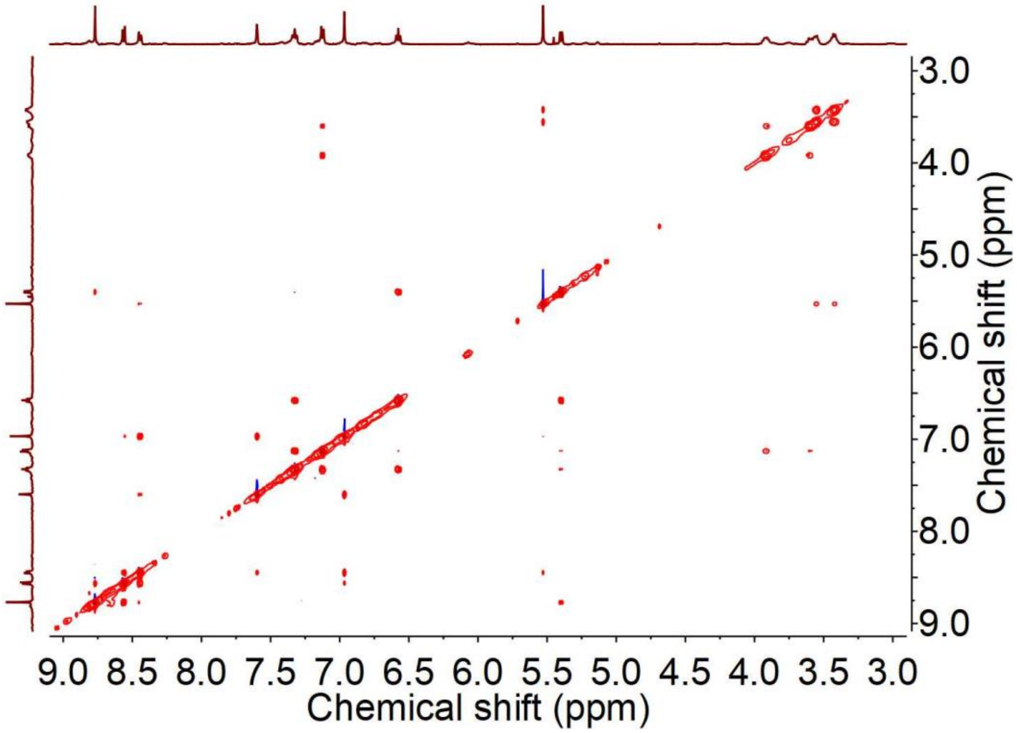

Supplementary Figure 36 | Partial ^1H–^1H NOESY spectrum (500 MHz, 298 K, CD_3CN) of **4**.

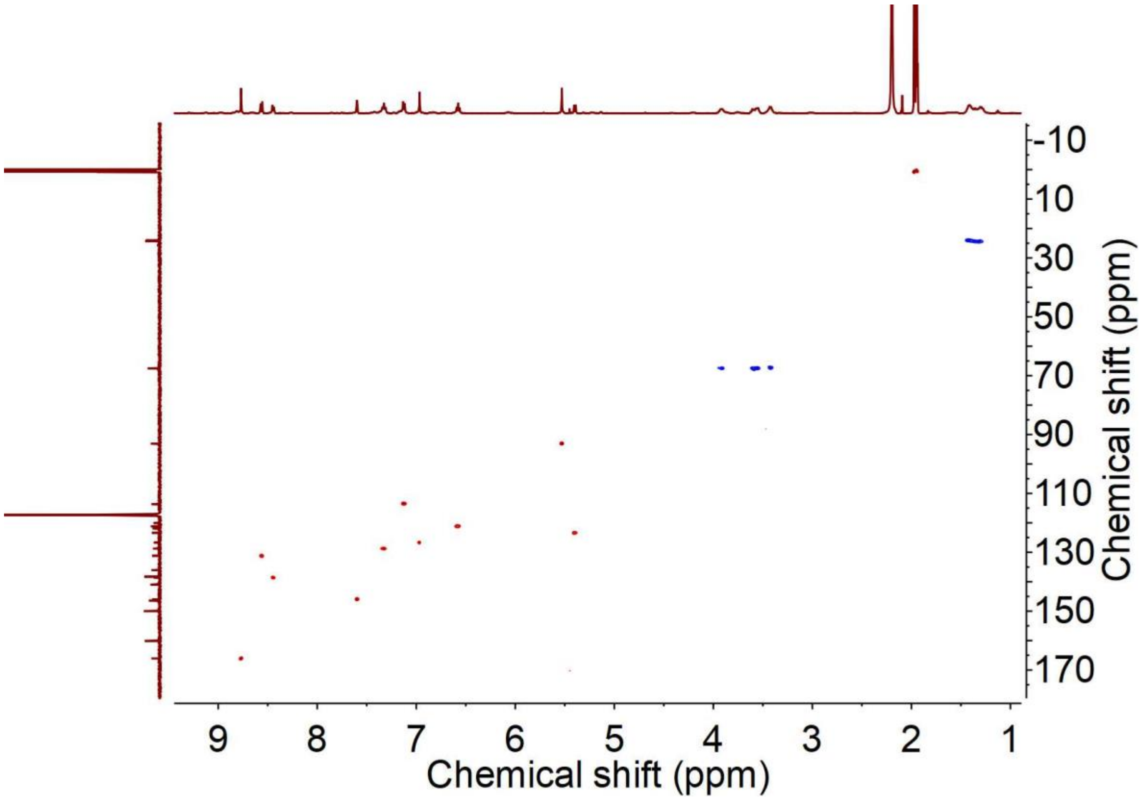

Supplementary Figure 37 | ^1H–^{13}C HSQC spectrum (500 MHz, 298 K, CD_3CN) of **4**.

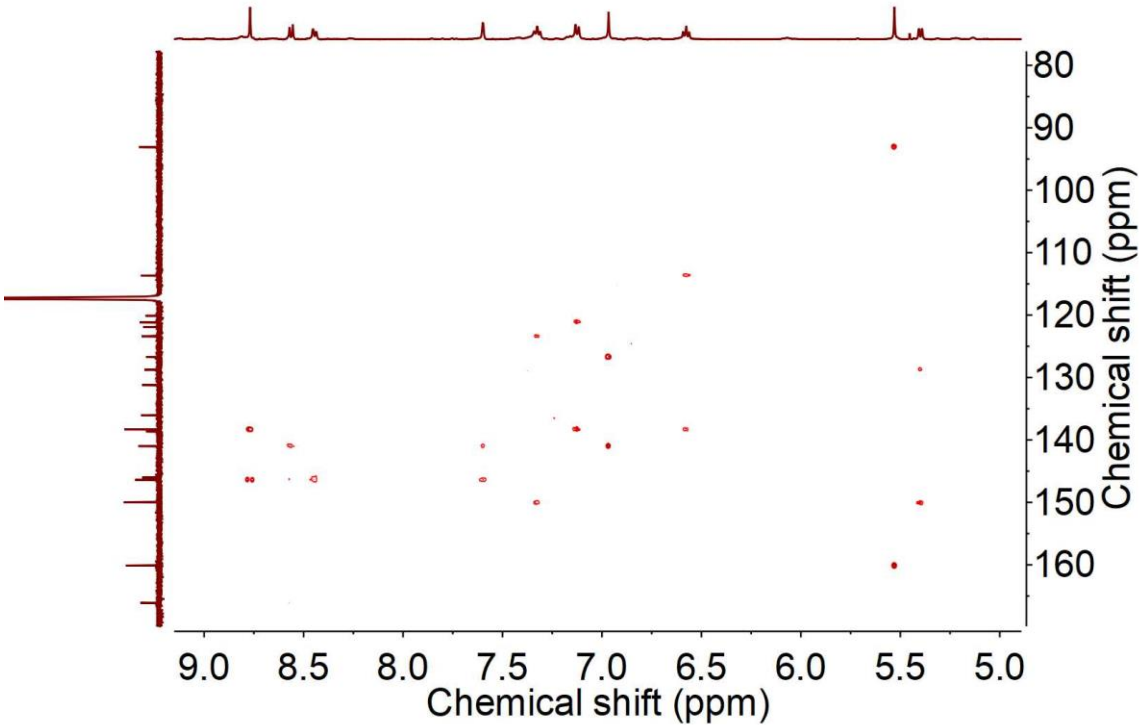

Supplementary Figure 38 | Aromatic region of ^{1}H–^{13}C HMBC spectrum (500 MHz, 298 K, CD_3CN) of **4**.

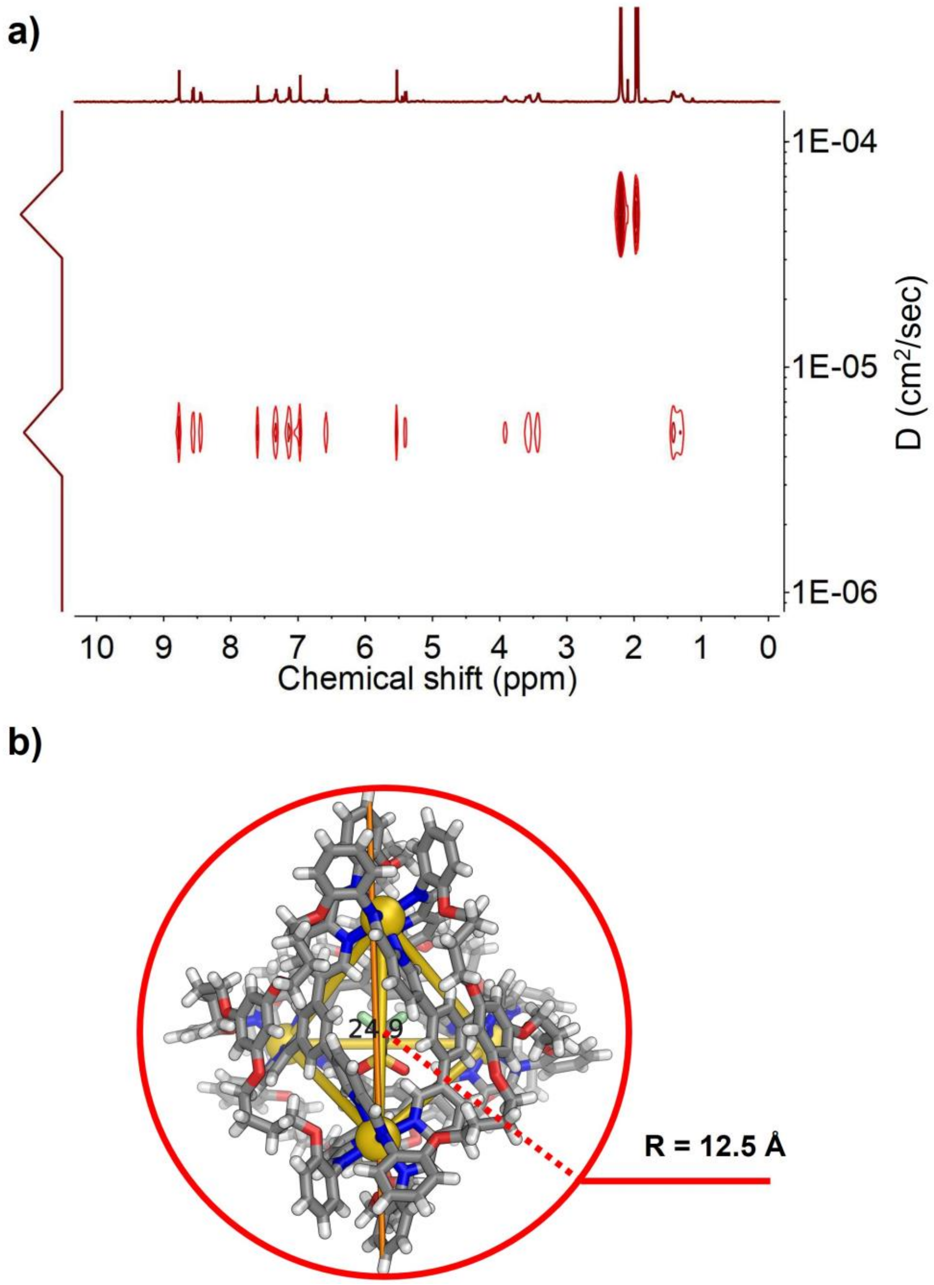

Supplementary Figure 39 | a) ^{1}H DOSY spectrum (400 MHz, 298 K, CD_3CN) of **4**. Diffusion coefficient: D = 5.10×10^{-10} m^2 s^{-1}, with radius of 12.8 Å; **b)** The radius of the crystal structure was measured to be 12.5 Å, consistent with the DOSY result obtained for **4**.

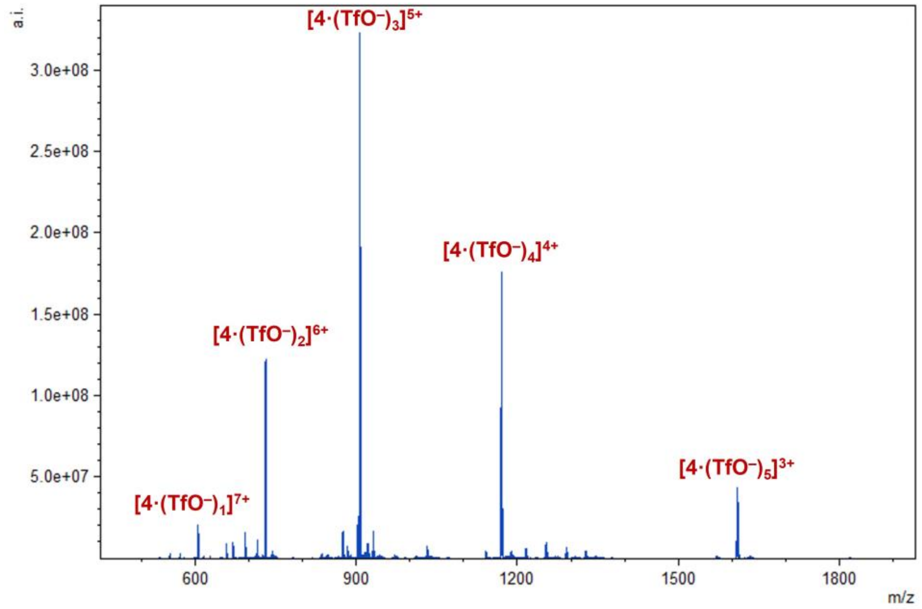

Supplementary Figure 40 | Low-resolution ESI-MS spectrum of **4**. *m/z* = 604.3 [**4**·(TfO$^-$)$_1$]$^{7+}$, 730.1 [**4**·(TfO$^-$)$_2$]$^{6+}$, 905.7 [**4**·(TfO$^-$)$_3$]$^{5+}$, 1169.4 [**4**·(TfO$^-$)$_4$]$^{4+}$, 1609.2 [**4**·(TfO$^-$)$_5$]$^{3+}$. Calculated peaks: m/z = 604.4 [**4**·(TfO$^-$)$_1$]$^{7+}$, 730.0 [**4**·(TfO$^-$)$_2$]$^{6+}$, 905.8 [**4**·(TfO$^-$)$_3$]$^{5+}$, 1169.6 [**4**·(TfO$^-$)$_4$]$^{4+}$, 1609.1 [**4**·(TfO$^-$)$_5$]$^{3+}$.

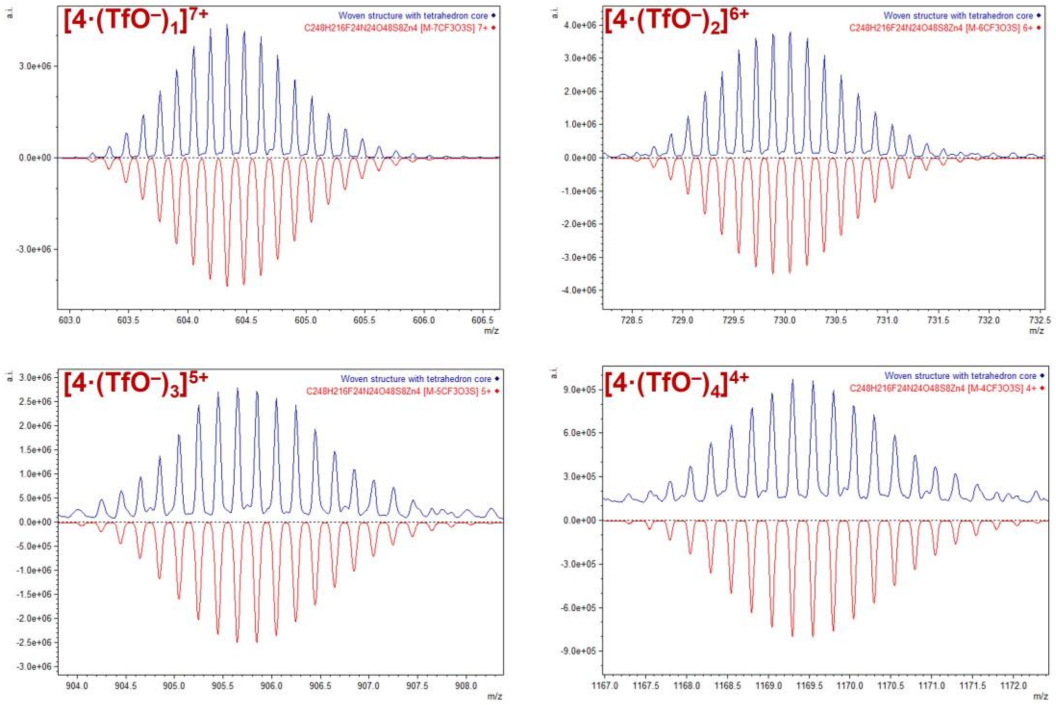

Supplementary Figure 41 | High-resolution ESI-mass spectrometry analysis of **4** showing the observed (top blue) and theoretical (bottom red) isotope patterns for the 7+, 6+, 5+ and 4+ peaks.

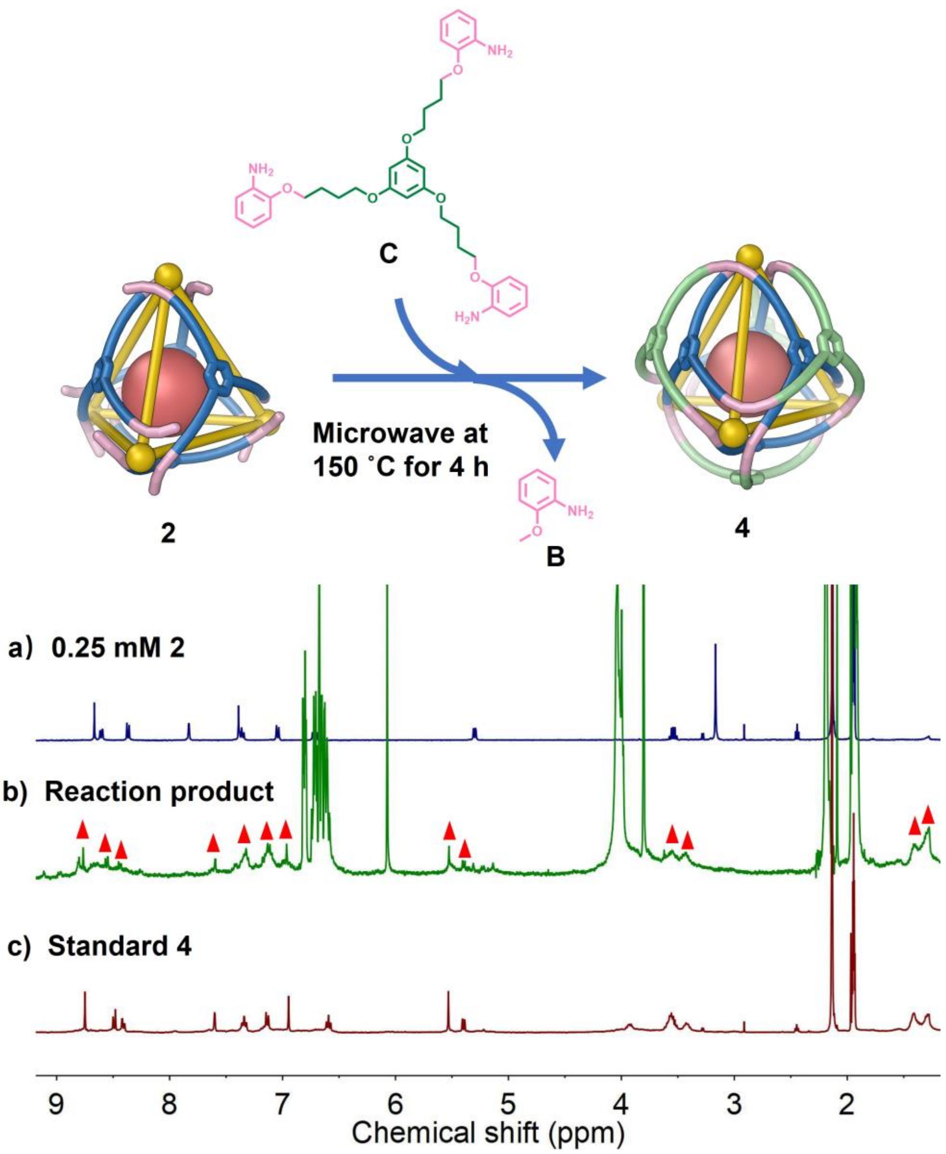

Supplementary Figure 42 | ^{1}H NMR spectra (400 MHz, CD_3CN) of a) 0.25 mM **2**; b) A 0.25 mM solution of **2** was treated with 8 equiv of triamine **C** under microwave irradiation at 150 ˚C for 4 h; c) Standard 0.25 mM **4**.

4. Guest exchange investigations

4.1 General methods

Guest exchange experiments were analysed by NMR spectroscopy. A mixed solution of 0.5 mM **2** or **4** containing 20 equiv of KOTf in CD_3CN was transferred to an NMR tube and 8 equiv of $TBAReO_4$ or $KSbF_6$ was added, respectively. In the case of **2**, due to the short time scale, the mixed solution was pre-cooled down to the target temperature in the NMR spectrometer before guest injection. The ^1H NMR spectra were measured directly after injection at the target temperatures. In the case of **4**, after guest addition, and the recording of ^1H NMR spectra, the tubes were kept in an oil bath at the target temperatures and measured at time intervals.

4.2 NMR spectra of the anionic guest exchange experiments (TfO^- and ReO_4^-)

4.2.1 NMR spectra following guest exchange within **2** at 298 K, 288 K, 278 K and 268 K

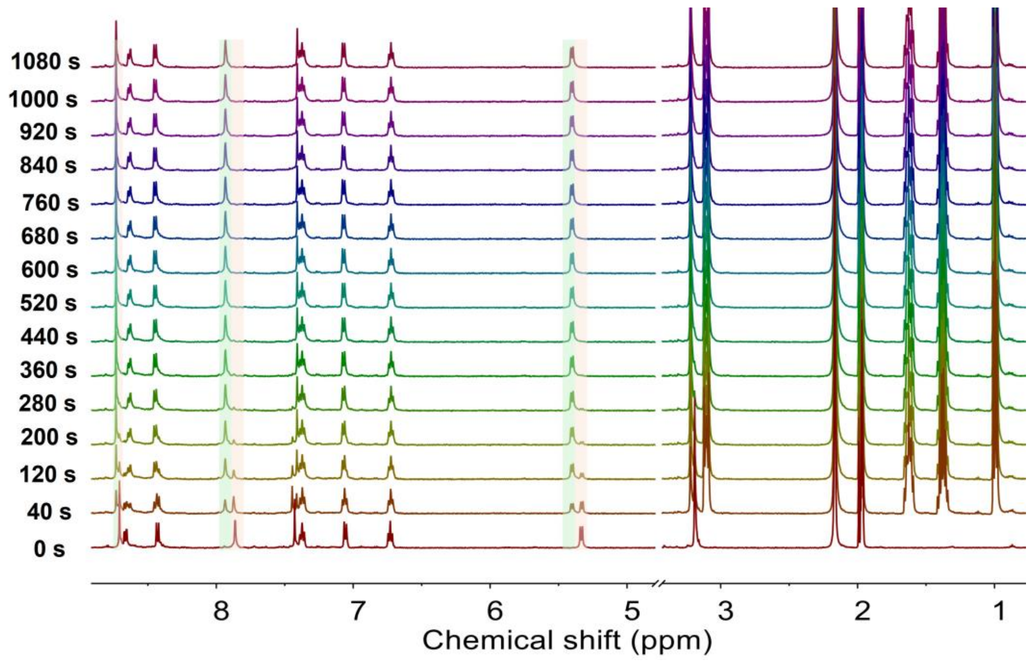

Supplementary Figure 43 | ^1H NMR spectra (400 MHz, CD_3CN) of a 0.5 mM solution of **2** containing 20 equiv KOTf, recorded following addition of 8 equiv $TBAReO_4$ at 298 K. Selected peaks for the TfO^- and ReO_4^- host-guest complexes are highlighted by light orange and light green shading respectively.

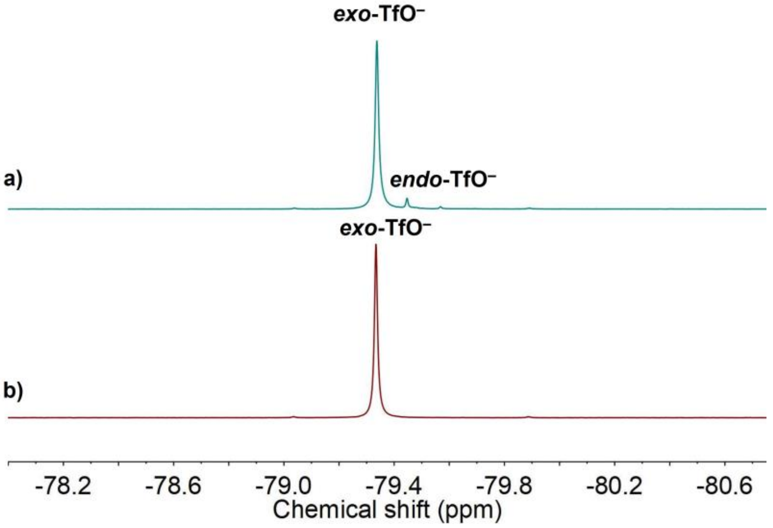

Supplementary Figure 44 | ^{19}F NMR spectra (376 MHz, CD_3CN) of **a)** a 0.5 mM solution of **2** in the presence of 20 equiv KOTf; **b)** the solution from **a)** after addition of 8 equiv $TBAReO_4$ following completion of the anionic guest exchange.

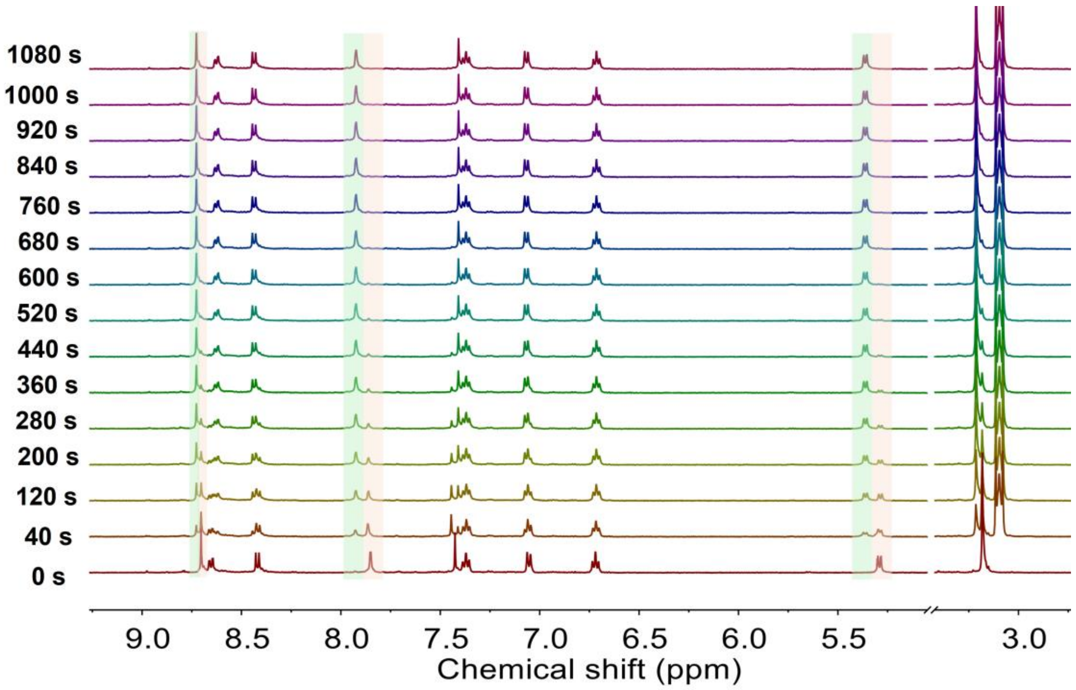

Supplementary Figure 45 | ^{1}H NMR spectra (400 MHz, CD_3CN) of a 0.5 mM solution of **2** containing 20 equiv KOTf, recorded following addition of 8 equiv $TBAReO_4$ at 288 K. Selected peaks for the TfO^- and ReO_4^- complexes are highlighted by light orange and light green shading respectively.

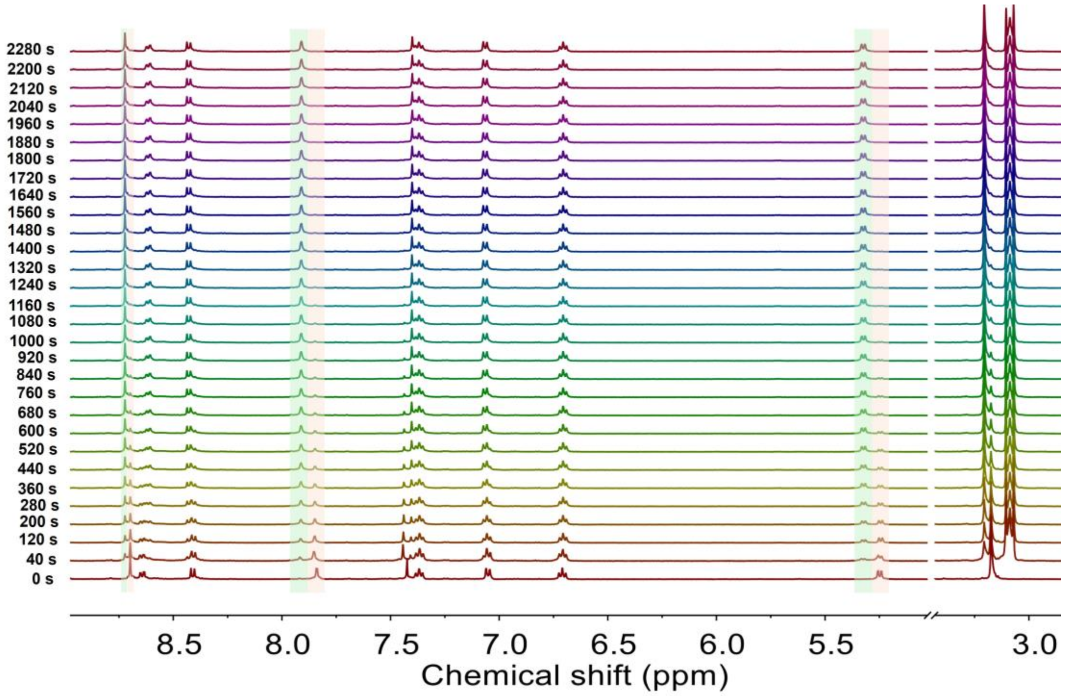

Supplementary Figure 46 | ^{1}H NMR spectra (400 MHz, CD_3CN) of a 0.5 mM solution of **2** containing 20 equiv KOTf, recorded following addition of 8 equiv $TBAReO_4$ at 278 K. Selected peaks for the TfO^- and ReO_4^- host-guest complexes are highlighted by light orange and light green shading respectively.

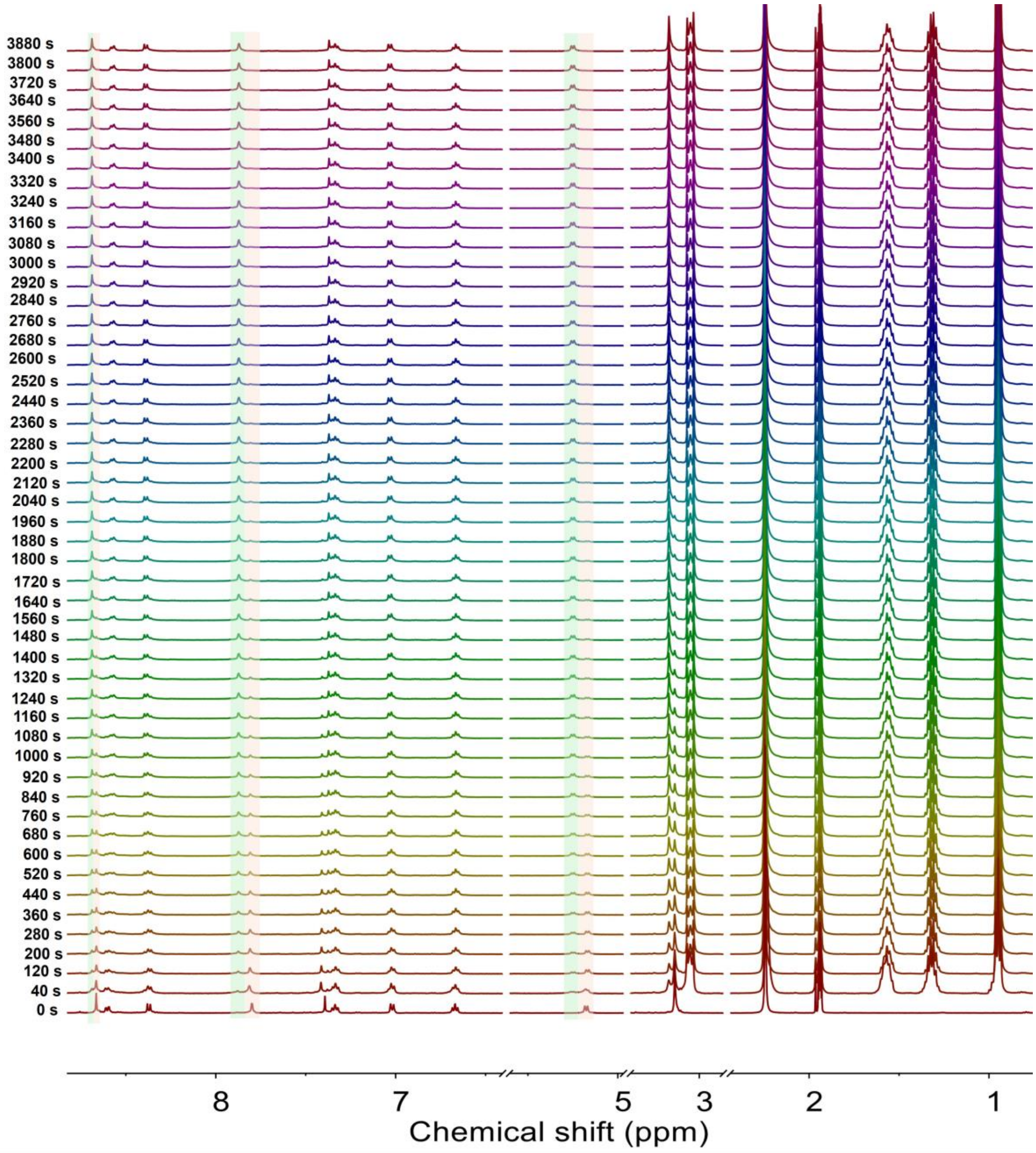

Supplementary Figure 47 | ^{1}H NMR spectra (400 MHz, CD_3CN) of a 0.5 mM solution of **2** containing 20 equiv KOTf, recorded following addition of 8 equiv $TBAReO_4$ at 268 K. Selected peaks for the TfO^- and ReO_4^- host-guest complexes are highlighted by light orange and light green shading respectively.

4.2.2 NMR spectra following guest exchange within **4** at 323 K, 313 K, 308 K and 298 K

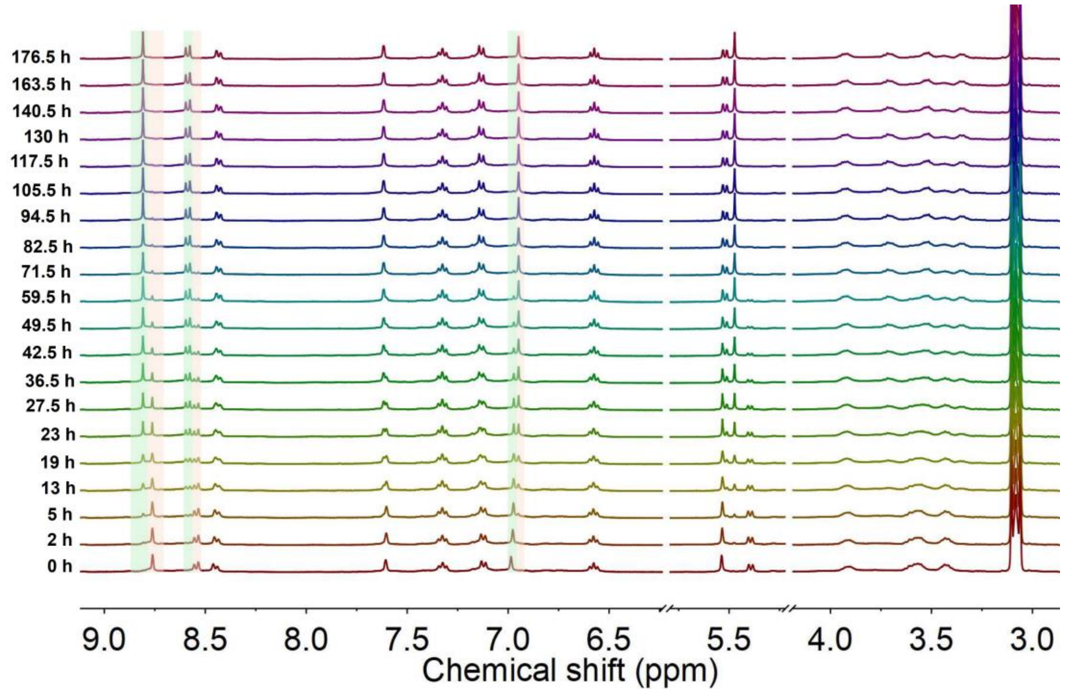

Supplementary Figure 48 | ^{1}H NMR spectra (400 MHz, CD_3CN) of a 0.5 mM solution of **4** containing 20 equiv KOTf, recorded following addition of 8 equiv $TBAReO_4$ at 323 K. Selected peaks for the TfO^{-} and ReO_4^{-} host-guest complexes are highlighted by light orange and light green shading respectively.

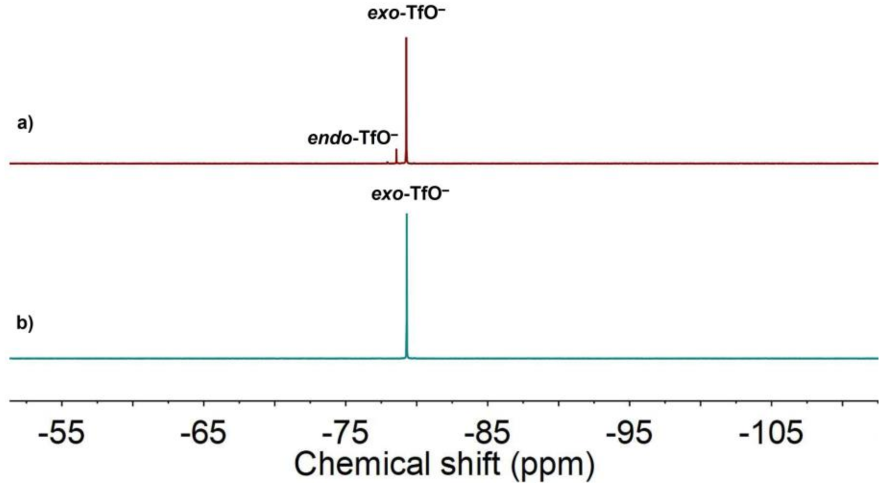

Supplementary Figure 49 | ^{19}F NMR spectra (298 K, 376 MHz, CD_3CN) of **a)** a 0.5 mM solution of **4** in the presence of 20 equiv KOTf; **b)** the solution from **a)** with addition of 8 equiv $TBAReO_4$, recorded after the completion of anionic guest exchange.

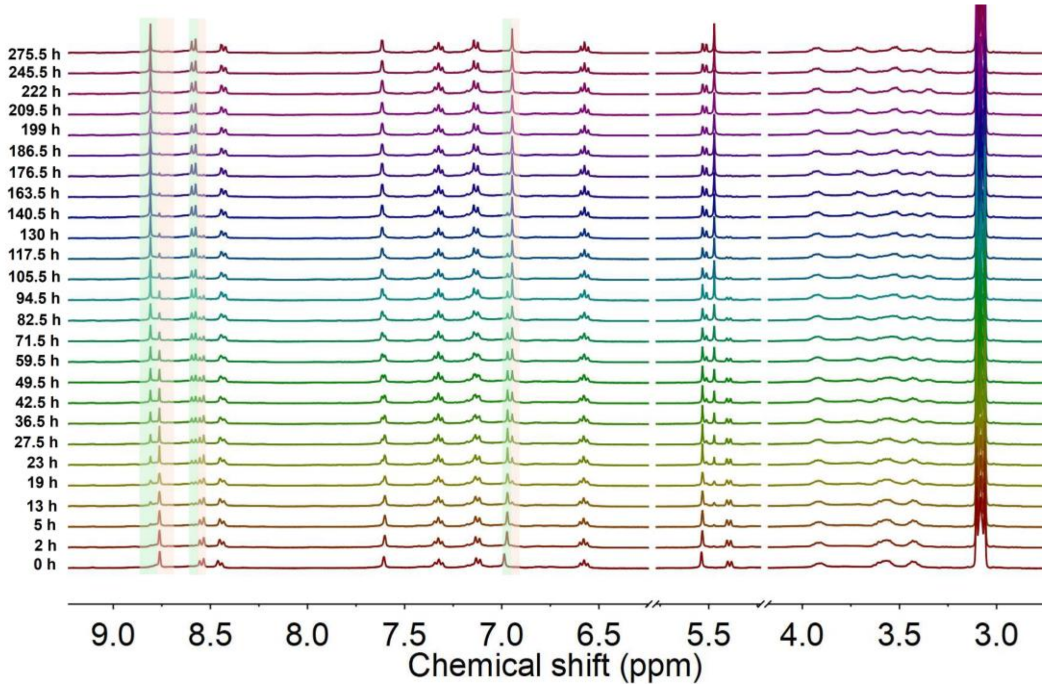

Supplementary Figure 50 | ^{1}H NMR spectra (400 MHz, CD_3CN) of a 0.5 mM solution of **4** containing 20 equiv KOTf, recorded following addition of 8 equiv $TBAReO_4$ at 313 K. Selected peaks for the TfO^- and ReO_4^- host-guest complexes are highlighted by light orange and light green shading respectively.

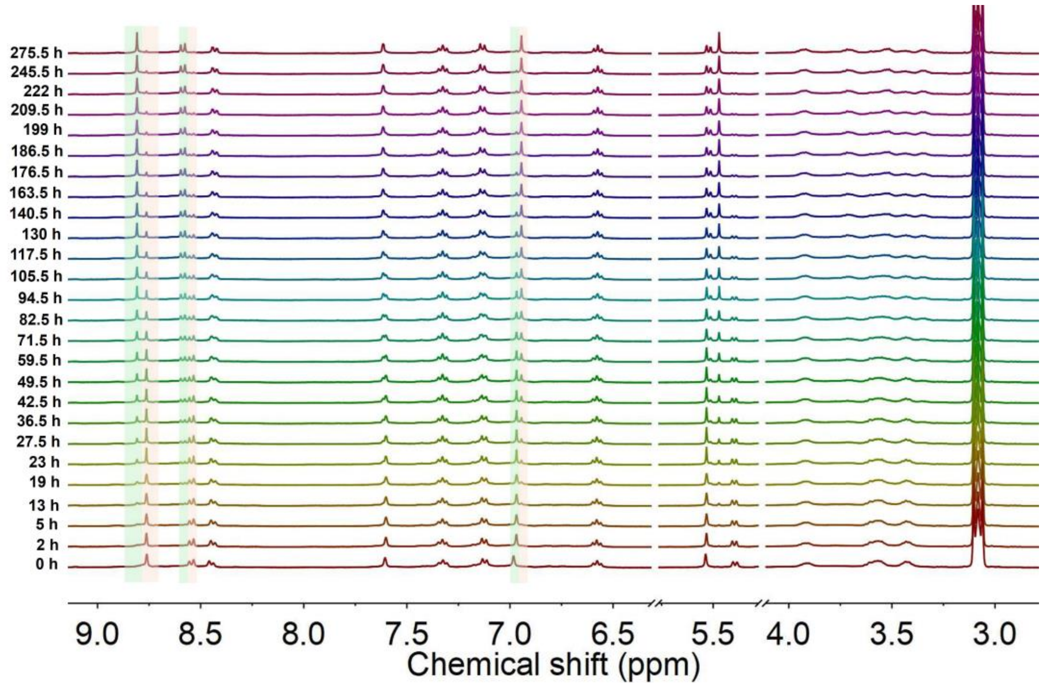

Supplementary Figure 51 | ^{1}H NMR spectra (400 MHz, CD_3CN) of a 0.5 mM solution of **4** containing 20 equiv KOTf, recorded following addition of 8 equiv $TBAReO_4$ at

308 K. Selected peaks for the TfO^- and ReO_4^- host-guest complexes are highlighted by light orange and light green shading respectively.

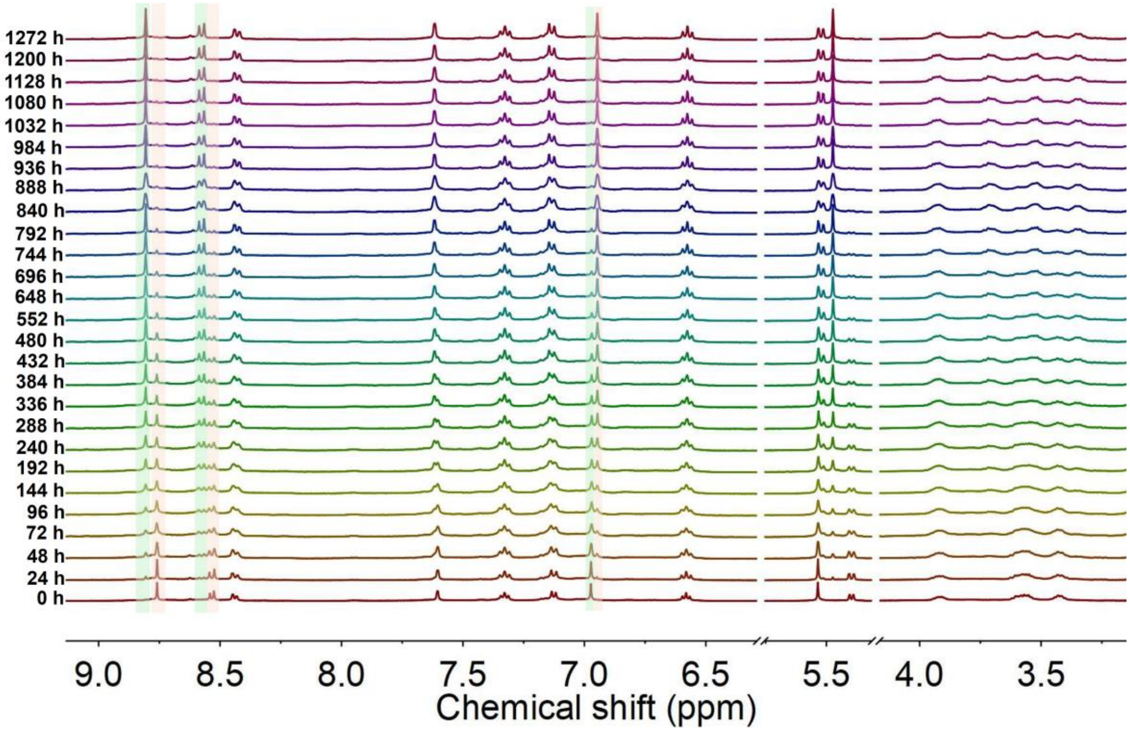

Supplementary Figure 52 | ^{1}H NMR spectra (400 MHz, CD_3CN) of a 0.5 mM solution of **4** containing 20 equiv KOTf, recorded following addition of 8 equiv $TBAReO_4$ at 298 K. Selected peaks for the TfO^- and ReO_4^- host-guest complexes are highlighted by light orange and light green shading respectively.

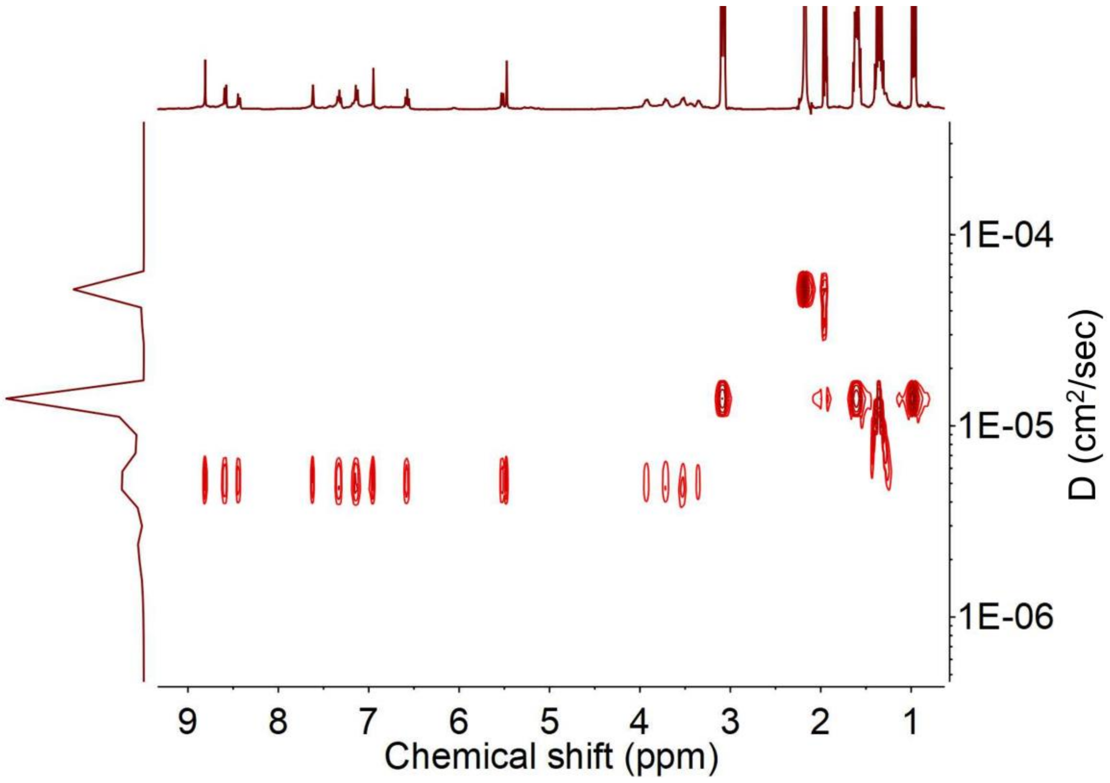

Supplementary Figure 53 | ^{1}H DOSY spectrum (400 MHz, 298K, CD_3CN) of $ReO_4^{-}\subset$**4**. Diffusion coefficient: D = 4.91×10^{-10} m^2 s^{-1}, corresponding to a hydrodynamic radius of 13.3 Å, consistent with the radius of **4** in Supplementary Figure 39.

4.3 Calculations of $\Delta H^{\ddagger}$ and $\Delta S^{\ddagger}$ of guest exchange

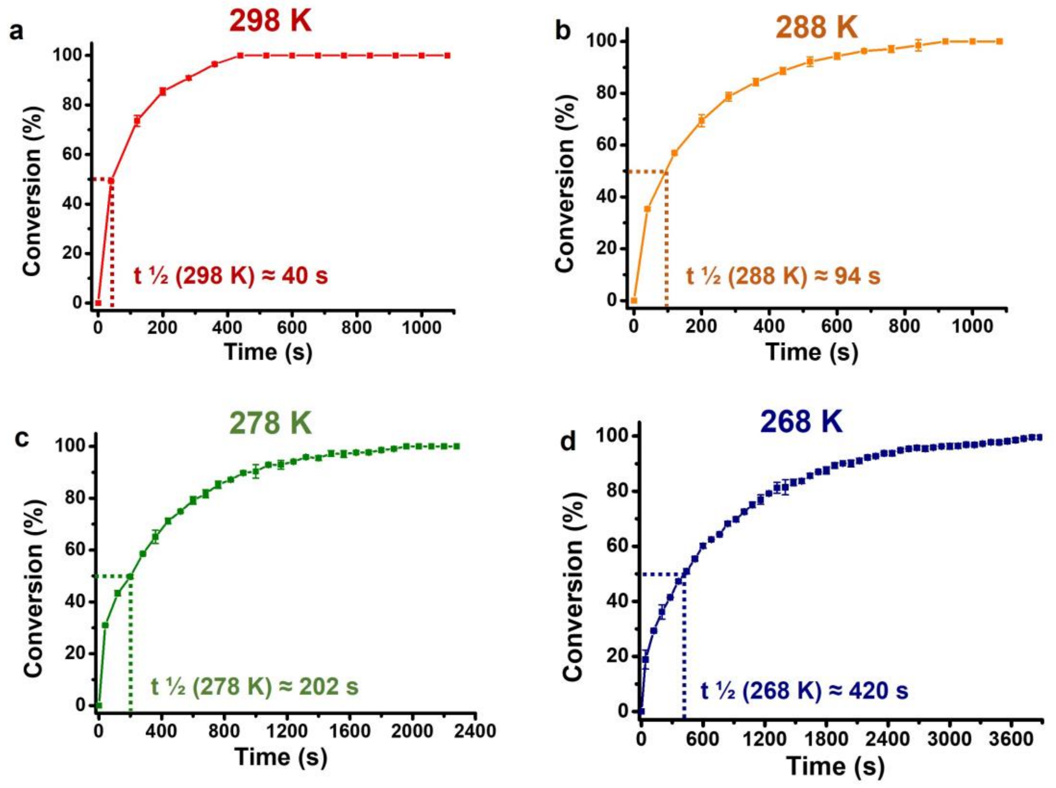

Supplementary Figure 54 | Time-conversion curves and half-life time ($t_{1/2}$) of anionic guest exchange within **2** at different temperatures **a)** 298 K; **b)** 288 K; **c)** 278 K and **d)** 268 K. The conversion data are presented as mean ± standard deviation, with each point calculated from six individual NMR signals (three pairs) corresponding to the chemical shifts of pair 1 (8.73 ppm, 8.70 ppm), pair 2 (7.92 ppm, 7.87 ppm), and pair 3 (5.36 ppm, 5.29 ppm).

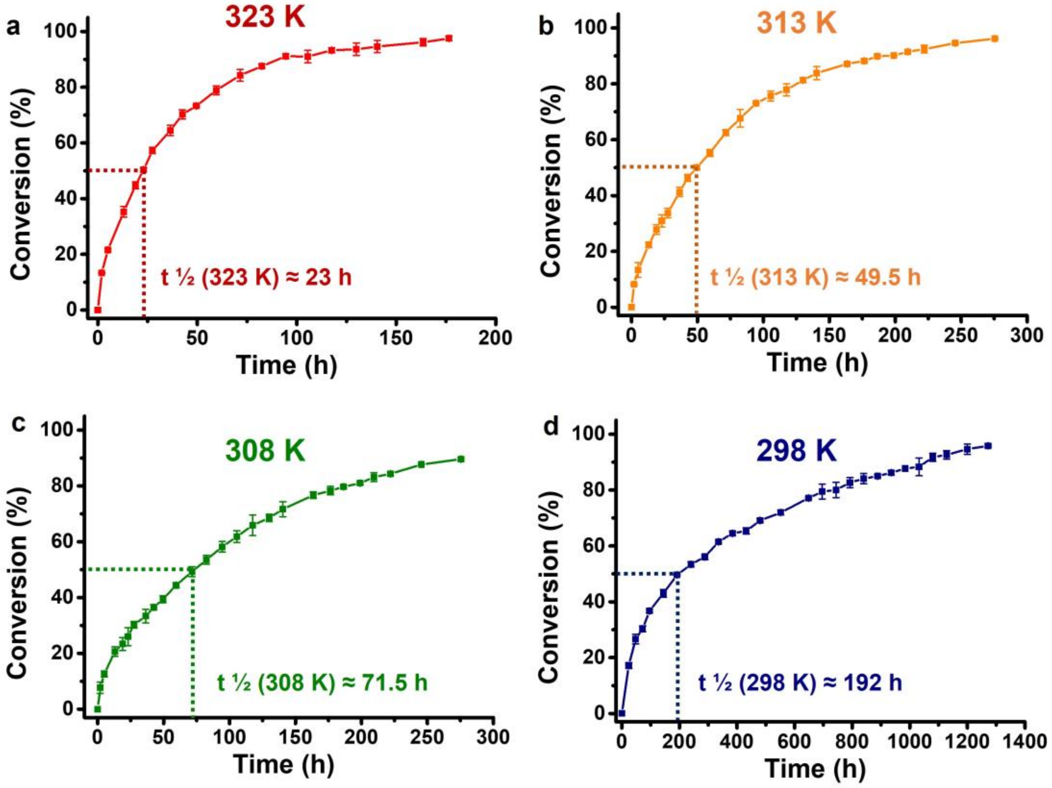

Supplementary Figure 55 | Time-conversion curves and half-life time ($t_{1/2}$) of anionic guest exchange within **4** at different temperatures. **a)** 323 K; **b)** 313 K; **c)** 308 K and **d)** 298 K. The conversion data are presented as mean ± standard deviation, with each point calculated from six individual NMR signals (three pairs) corresponding to the chemical shifts of pair 1 (8.81 ppm, 8.76 ppm), pair 2 (8.59 ppm, 8.54 ppm), and pair 3 (6.97 ppm, 6.95 ppm).

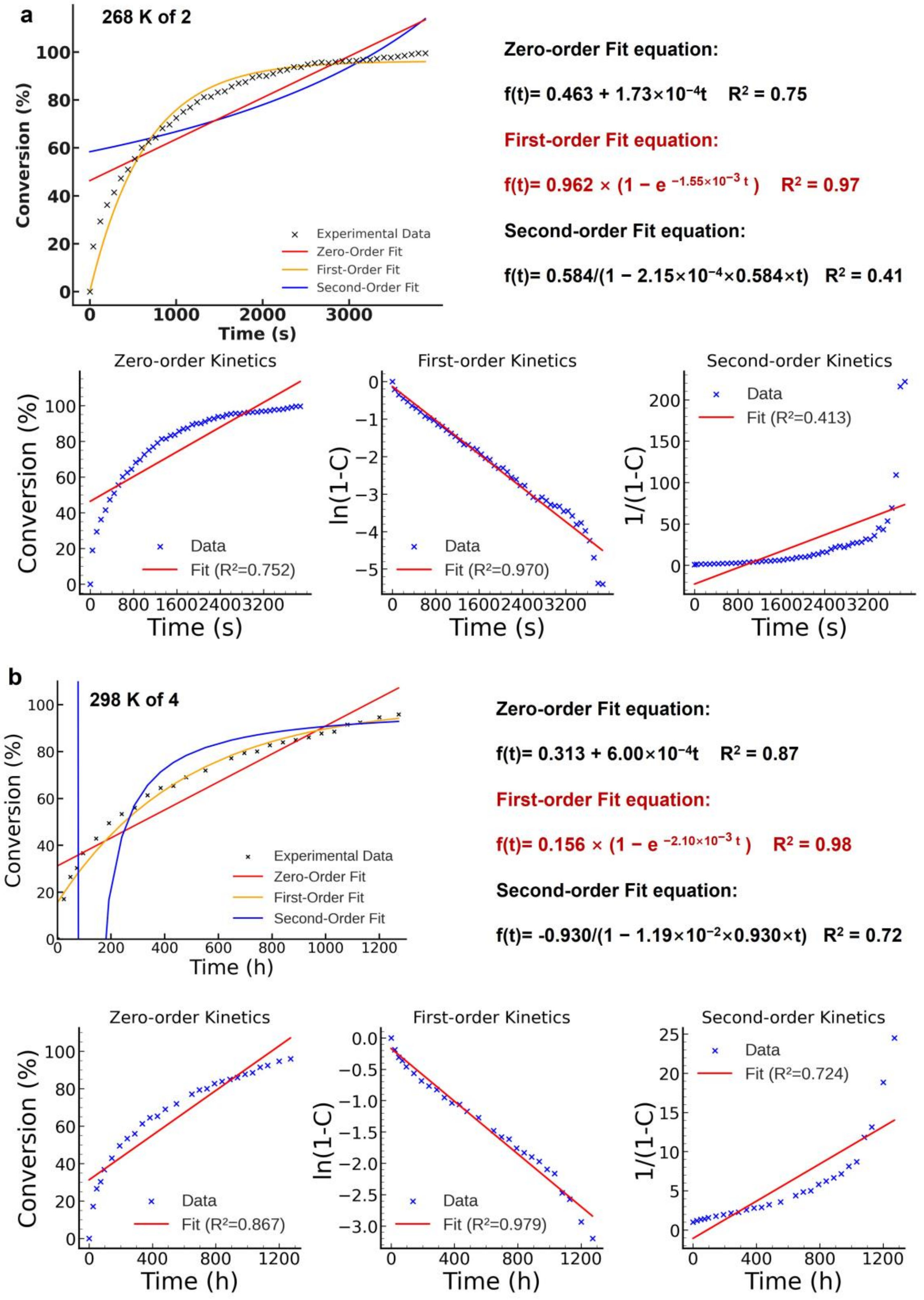

Supplementary Figure 56 | Data fitting with different rate order equations. **a)** Fitting with the data from anionic guest exchange within **2** at 268 K; **b)** Fitting with the data from anionic guest exchange within **4** at 298 K.

The half-life data are provided at four temperatures: 298 K, 288 K, 278 K, and 268 K for anionic guest exchange within **2** (Supplementary Figure 54 and Table 1); 323 K, 313 K, 308 K, and 298 K for anionic guest exchange within **4** (Supplementary Figure 55 and Table 2). During the course of the anion exchange reaction, the binding ratio between ReO_4^- and the cage structures is inferred to be 1:1,[1] with no detectable signals corresponding to any intermediate compounds. During the anionic exchange process, an excess of ReO_4^- (8 equiv) was employed relative to TfO^-⊂**2** or TfO^-⊂**4**. Kinetic data were subsequently fitted to reaction models of varying orders, among which only the First-order model provided an acceptable fit, as shown in Supplementary Figure 55. Based on these observations, we postulate that the anion exchange reaction can be considered a *pseudo*-first-order reaction.

Then, the rate constant (k) is calculated from the half-life ($t_{1/2}$) using the formula:

$$k = \ln(2)/t_{1/2}$$

The calculated rate constants are given in Supplementary Tables 1 and 2 below:

Supplementary Table 1 | Rate constants of guest exchange within **2** at different temperatures

Temperature (K)	Half-life (s)	Rate constant (s^{-1})	ln(k/T)
298	40	0.01733	-9.75
288	94	0.00737	-10.57
278	202	0.00343	-11.30
268	420	0.00165	-11.99

Supplementary Table 2 | Rate constants of guest exchange within **4** at different temperatures

Temperature (K)	Half-life (s)	Rate constant ($\times10^{-6}$ s^{-1})	ln(k/T)
323	82800	8.37134	-17.47
313	178200	3.88971	-18.20
308	257400	2.69288	-18.55
298	691200	1.00282	-19.51

From the $\ln(k/T)$ at different temperatures (T), we can obtain the $\Delta H^{\ddagger}$ and $\Delta S^{\ddagger}$of anionic guest exchange with the linear fitting method, as shown in Supplementary Figure 57

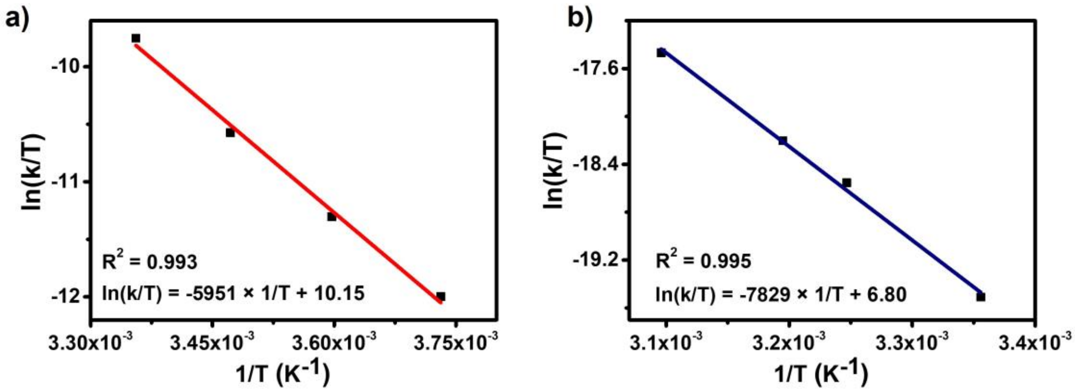

Supplementary Figure 57 | Linear fitting of $\ln(k/T)$ to reaction temperature (T) to give the changes of enthalpy ($\Delta H^{\ddagger}$) and entropy ($\Delta S^{\ddagger}$) of the anionic guest exchange within **a) 2**; **b) 4**.

Using the Eyring equation:

$$\ln(k/T) = -(\Delta H^{\ddagger}/R) \times (1/T) + \Delta S^{\ddagger}/R + \ln\,(k_B/h)$$

Calculated results of enthalpy ($\Delta H^{\ddagger}$) and entropy ($\Delta S^{\ddagger}$) of activation for anionic guest exchange within **2**

$\Delta H^{\ddagger}$ = 49.5 ± 2.4 kJ·mol^{-1}
$\Delta S^{\ddagger}$ = -（113.1 ± 11.5) J·mol^{-1}K^{-1}

Calculated results of enthalpy ($\Delta H^{\ddagger}$) and entropy ($\Delta S^{\ddagger}$) of activation for anionic guest exchange within **4**

$\Delta H^{\ddagger}$ = 65. 1 ± 2.4 kJ·mol^{-1}
$\Delta S^{\ddagger}$ = - (141.0 ± 9.9) J·mol^{-1}K^{-1}

4.4 Calculations of apparent activation energy and pre-exponential factor

According to the basic principles of chemical kinetics, when the initial concentrations of the reactants are kept constant, the relationship between reaction rate (k) and half-life ($t_{1/2}$) follows:

$$t_{1/2} \propto \frac{1}{k} \text{ or } t_{1/2} = \frac{C}{k} \qquad (C = const)$$

where $t_{1/2}$ can be obtained directly from the time-conversion curves. From our experiments, $t_{1/2}$ are shown in Supplementary Figures 54 and 55.

According to the Arrhenius formula:

$$k = Ae^{-\frac{Ea}{RT}}$$

where E_a is the apparent activation energy, A is the pre-exponential factor, R is the universal gas constant and T is temperature. Thus, we can obtain:

$$\frac{C}{t_{1/2}} = Ae^{-\frac{Ea}{RT}}$$

And then the equation can be further transformed to be:

$$\ln(t_{1/2}) = \frac{Ea}{R} \times \frac{1}{T} + lnC - lnA$$

From the half-life ($t_{1/2}$) at different temperatures (T), we can obtain the apparent activation energy (E_a) of anionic guest exchange with the linear fitting method, as shown in Supplementary Figure 58.

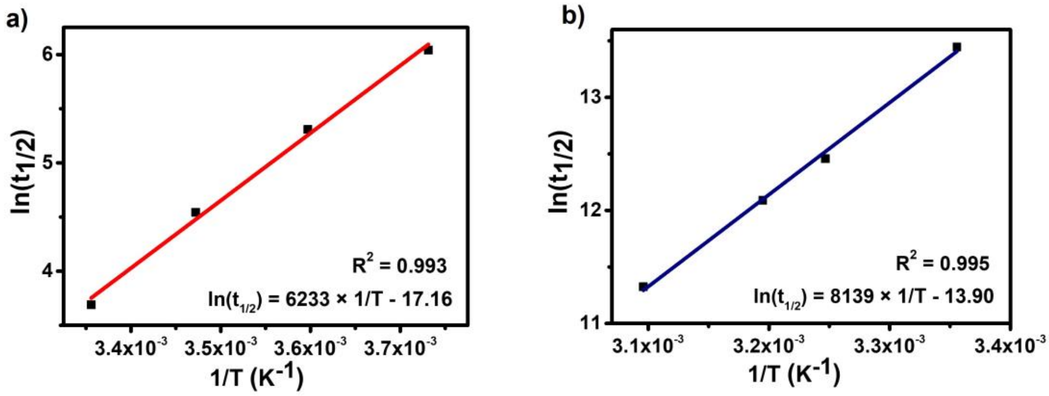

Supplementary Figure 58 | Linear fitting of half-life ($t_{1/2}$) to reaction temperature (T) to give the apparent activation energy (E_a) and pre-exponential factor (A) of the anionic guest exchange within **a) 2**; **b) 4**.

E_a calculation for 2

From the given equation $\ln(t_{1/2}) = 6233\times\frac{1}{T}$ - 17.16, the activation energy E_a is calculated using the relationship:

$\frac{Ea}{R} \approx$ 6233, where R is the universal gas constant (8.314 J/(mol·K)). We thus have:

$E_a \approx$ 6233 × 8.314 = 51820 J/mol = 51.82 kJ/mol.

E_a calculation for 4

From the given equation $\ln(t_{1/2}) = 8139\times\frac{1}{T}$ - 13.90, the activation energy E_a is calculated similarly:

$E_a \approx$ 8139 × 8.314 = 67620 J/mol = 67.62 kJ/mol.

Calculations for pre-exponential factor (A)

The pre-exponential factor (A) was calculated under the assumption of a *pseudo*-first-order reaction model.

Pre-exponential factor (A) calculation for 2

The constant term (-17.1643) in the equation is related to ln(A) through the relationship:

$\ln(t_{1/2}) = \ln(\ln(2)) - \ln(A)$.

Using $\ln(\ln(2)) \approx -0.3665$, we have:

$\ln(A) = 17.1643 - 0.3665 = 16.7978$.

$A = \exp(16.7978) \approx 2 \times 10^7\ s^{-1}$.

Pre-exponential factor (A) calculation for 4

For the pre-exponential factor (A), the constant term (-13.9036) is used as follows:

$\ln(A) = (13.9036) - 0.3665 = 13.5371$.

$A = \exp(13.5371) \approx 8 \times 10^5\ s^{-1}$.

4.5 Proposed mechanism

The measured activation parameters indicate that knotted cage **4** imposes significant mechanical restrictions on guest exchange. The observed increase in activation enthalpy ($\Delta H^{\ddagger}$) and the more unfavorable activation entropy ($\Delta S^{\ddagger}$) for guest exchange in **4** suggest that conformational constraints present a barrier to guest escape from **4**. Molecular dynamics (MD) simulations further support this conclusion, revealing that the apertures within the tetrahedral core of **4** exhibit a reduction in size between adjacent faces, leading to a ~0.3 Å decrease in the largest aperture width (Supplementary Fig. 96). This contraction, induced by the steric hindrance of the knotted cage framework, mechanically suppresses the dynamic motion of the tetrahedral core, effectively suppressing guest release.

Furthermore, guest release in the knotted cage may also require pre-organization of the

outer layer before guest ejection can occur. While this hypothesis remains to be validated fully, we posit that the release pathway involves local distortions or cooperative rearrangements of the ligand framework, transiently increasing aperture size to facilitate guest exchange. In contrast, **2** follows a lower-activation-energy pathway and increased frequency of effective collisions. These findings underscore the role of knotting in modulating guest exchange dynamics, where steric constraints and restricted aperture sizes contribute to the observed kinetic barriers to guest exchange.

4.5 NMR spectra of the anionic guest exchange experiments (TfO^- and SbF_6^-)

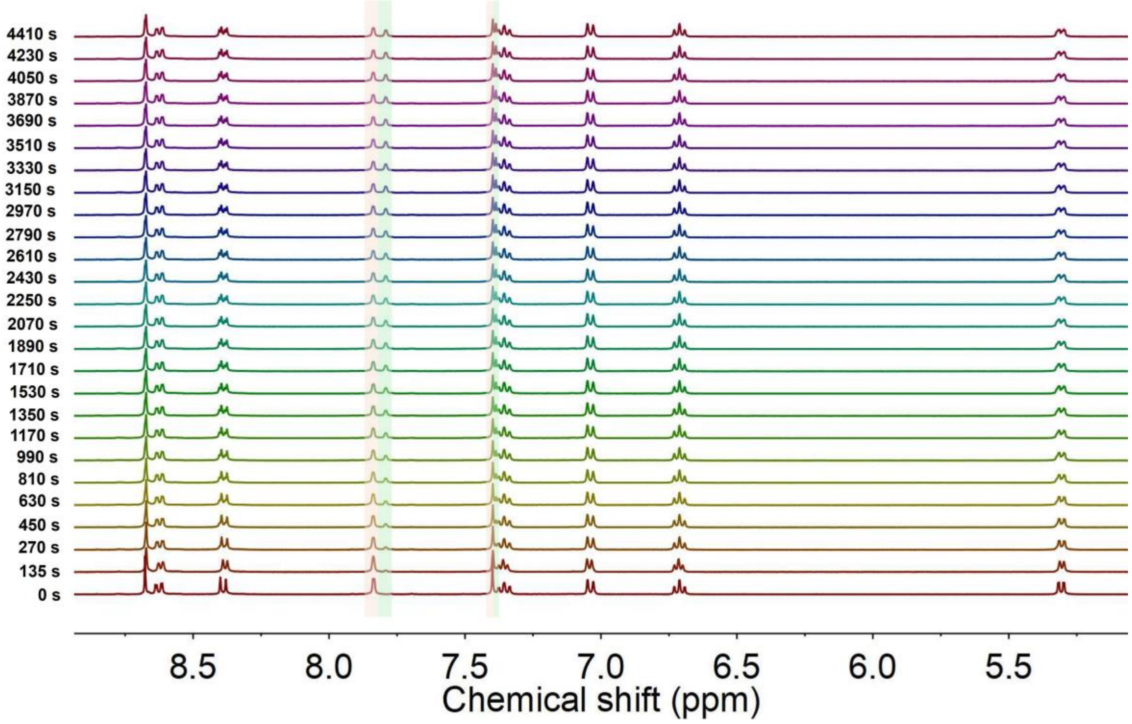

Supplementary Figure 59 | ^1H NMR spectra (400 MHz, CD_3CN) of a 0.5 mM solution of **2** containing 20 equiv KOTf, recorded following addition of 8 equiv $KSbF_6$ at 298 K. Selected partial peaks for the TfO^- and SbF_6^- host-guest complexes are highlighted by orange and green shading respectively.

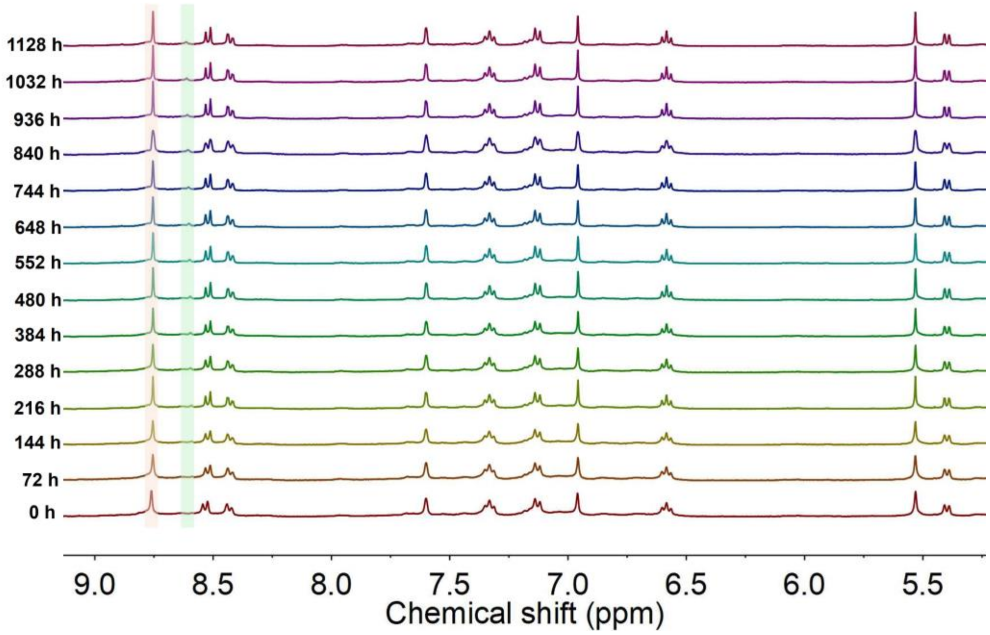

Supplementary Figure 60 | ^1H NMR spectra (400 MHz, CD_3CN) of a 0.5 mM solution of **4** containing 20 equiv KOTf, recorded following addition of 8 equiv $KSbF_6$ at 298 K. Selected partial peaks for the TfO^- and SbF_6^- host-guest complexes are highlighted by orange and green shading respectively.

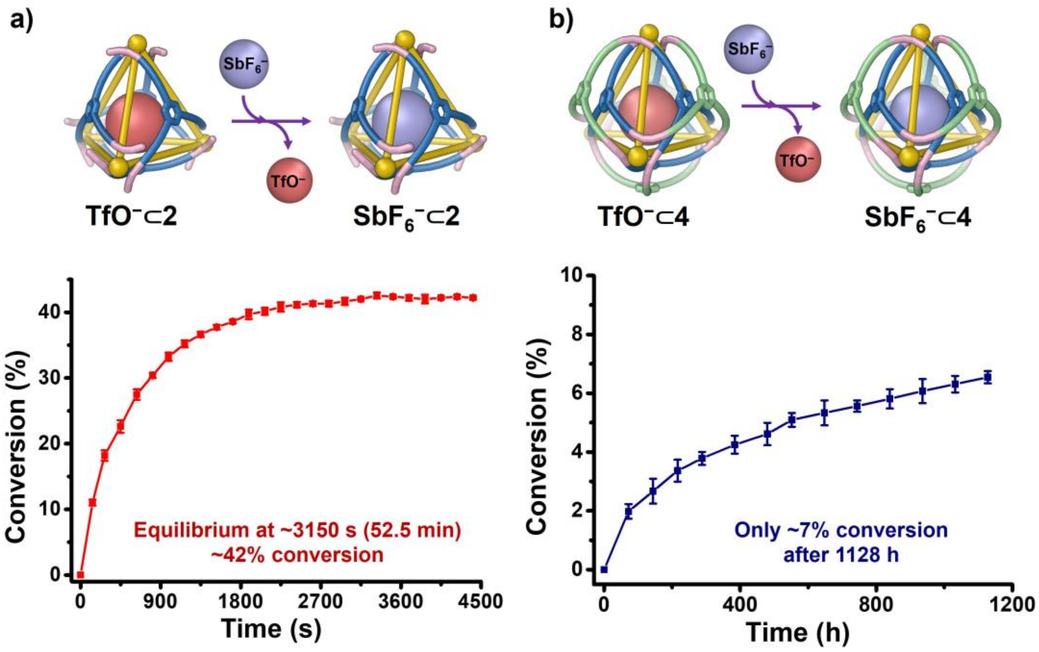

Supplementary Figure 61 | Time-conversion curves of the displacement of TfO^- by SbF_6^- at 298 K within **a)** cage **2**; and **b)** cage **4**. The conversion data are presented as mean ± standard deviation, with each point calculated from six individual NMR signals (three pairs) corresponding to the chemical shifts of **a)** pair 1 (8.39 ppm, 8.38 ppm), pair 2 (7.84 ppm, 7.79 ppm), and pair 3 (7.40 ppm, 7.39 ppm); **b)** pair 1 (8.76 ppm, 8.60 ppm), pair 2 (7.17 ppm, 7.13 ppm), and pair 3 (5.53 ppm, 5.45 ppm).

5. Robustness investigations

5.1 Experimental details

Robustness investigations were performed in NMR tubes using a 0.25 mM (0.5 mL) CD_3CN solution of the respective cage. The stimulus noted below (d_6-DMSO, D_2O, HCl (125 mM acetonitrile solution) or 30% H_2O_2 (aq)) was then added, and after heating the solution for 20 h at 50°C or shaking the sample for 10 seconds at room temperature, a ^1H NMR spectrum was taken. The number of equivalents listed below corresponds to the cumulative total quantity added to the solution.

5.2 Addition of d_6-DMSO

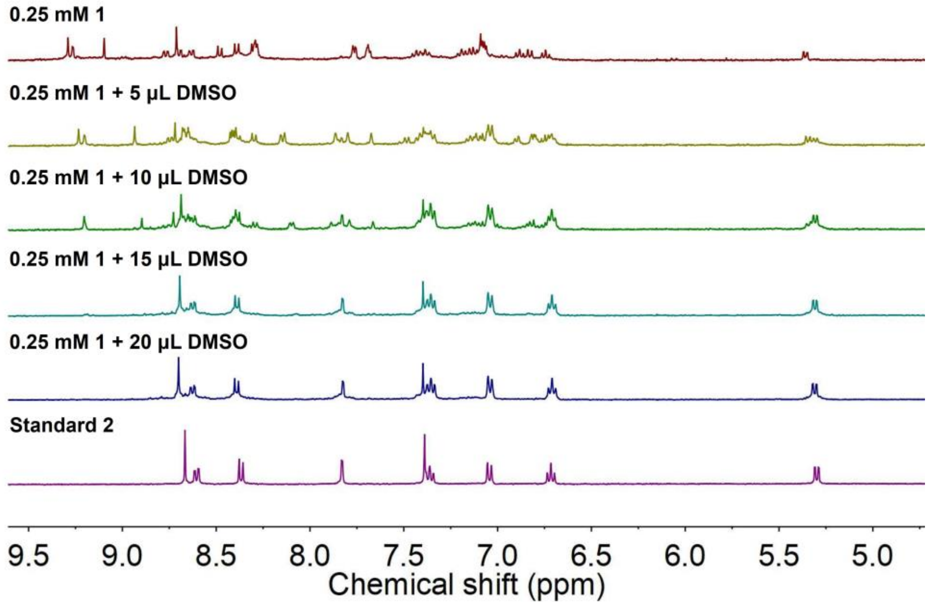

Supplementary Figure 62 | ^{1}H NMR spectra of the titration of 0.25 mM **1** with different amounts of d_6-DMSO (400 MHz, CD_3CN, 298 K). The volume noted corresponds to the cumulative DMSO volume added. Full transformation from **1** to **2** was observed following addition of 20 µL d_6- DMSO.

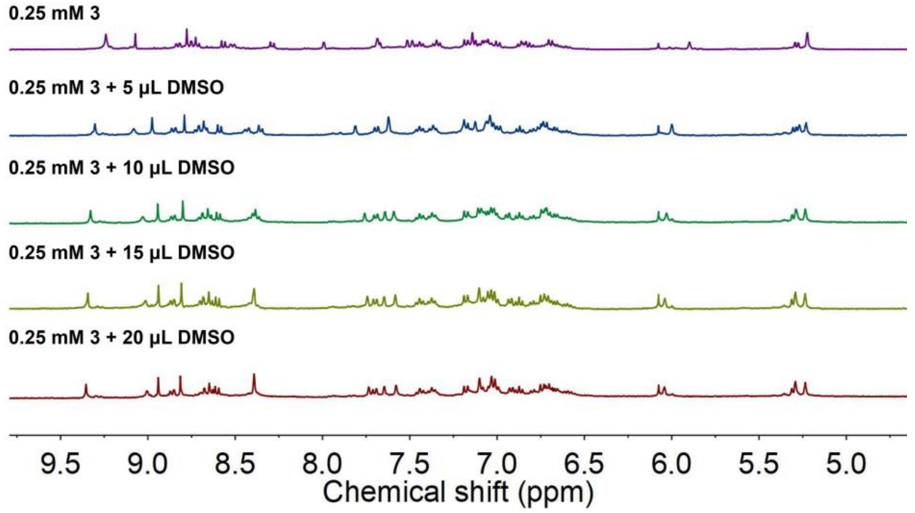

Supplementary Figure 63 | ^{1}H NMR spectra of the titration of 0.25 mM **3** with different amounts of d_6-DMSO (400 MHz, CD_3CN, 298 K). The volume noted corresponds to the cumulative DMSO volume added to the solution. No obvious decomposition was observed following addition of 20 µL d_6- DMSO.

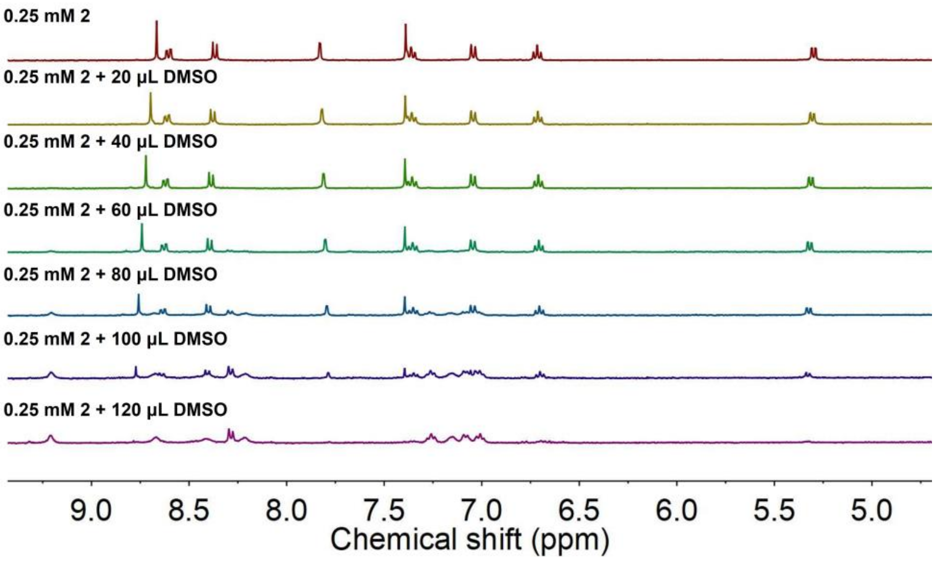

Supplementary Figure 64 | ^{1}H NMR spectra of the titration of 0.25 mM **2** with different amounts of *d₆*-DMSO (400 MHz, CD_3CN, 298 K). The volume noted corresponds to the cumulative DMSO volume added to the solution. Full decomposition was observed following addition of 120 µL d_6-DMSO.

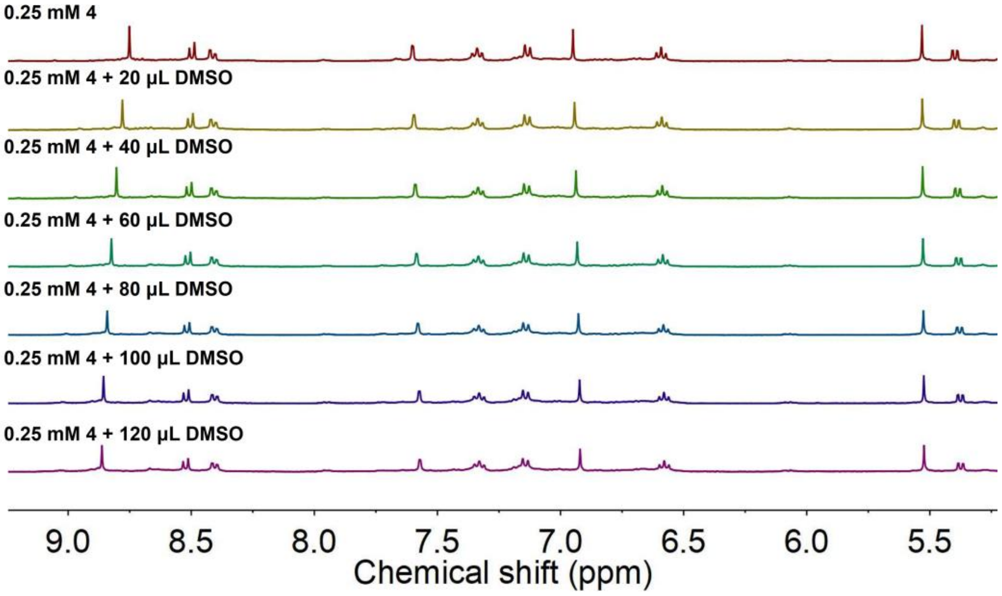

Supplementary Figure 65 | ^{1}H NMR spectra of the titration experiment of 0.25 mM **4** with different amounts of d_6-DMSO (400 MHz, CD_3CN, 298 K). The volume noted corresponds to the cumulative DMSO volume added to the solution. No obvious decomposition was observed following addition of 120 µL d_6- DMSO.

5.3 Addition of D_2O

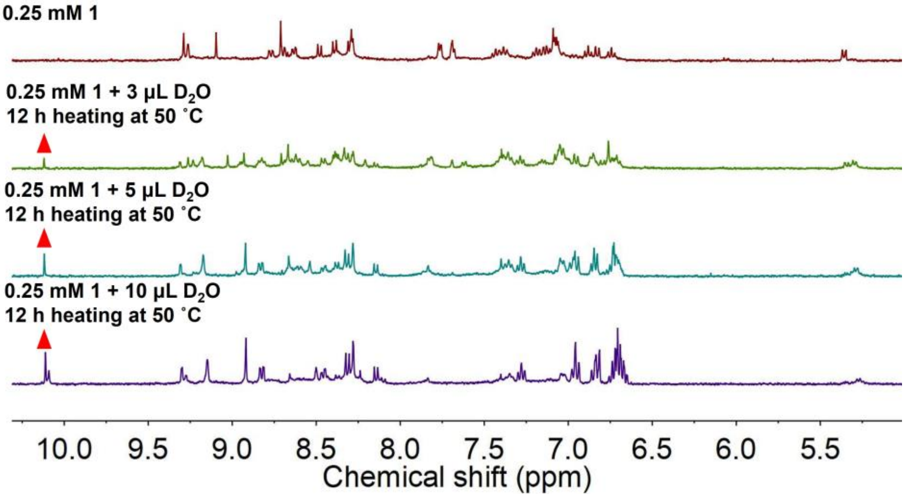

Supplementary Figure 66 | ^1H NMR spectra of the titration experiment of 0.25 mM **1** with different amounts of D_2O (400 MHz, CD_3CN, 298 K). The volume noted corresponds to the cumulative D_2O volume added to the solution. The aldehyde signal is marked with a red triangle. Full decomposition of **1** was observed following addition of 10 μL D_2O.

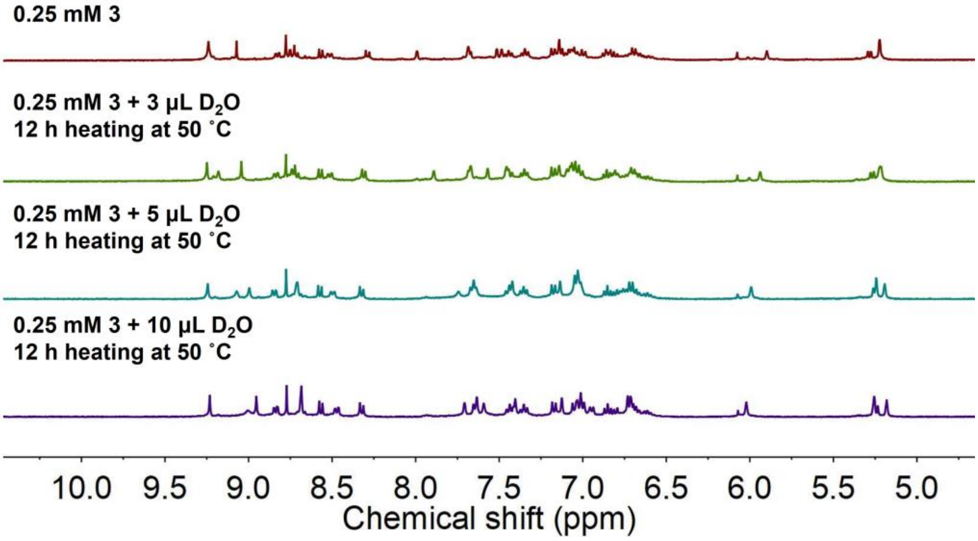

Supplementary Figure 67 | ^1H NMR spectra of the titration experiment of 0.25 mM **3** with different amounts of D_2O (400 MHz, CD_3CN, 298 K). The volume noted corresponds to the cumulative D_2O volume added to the solution. No obvious decomposition of **3** was observed following addition of 10 μL D_2O.

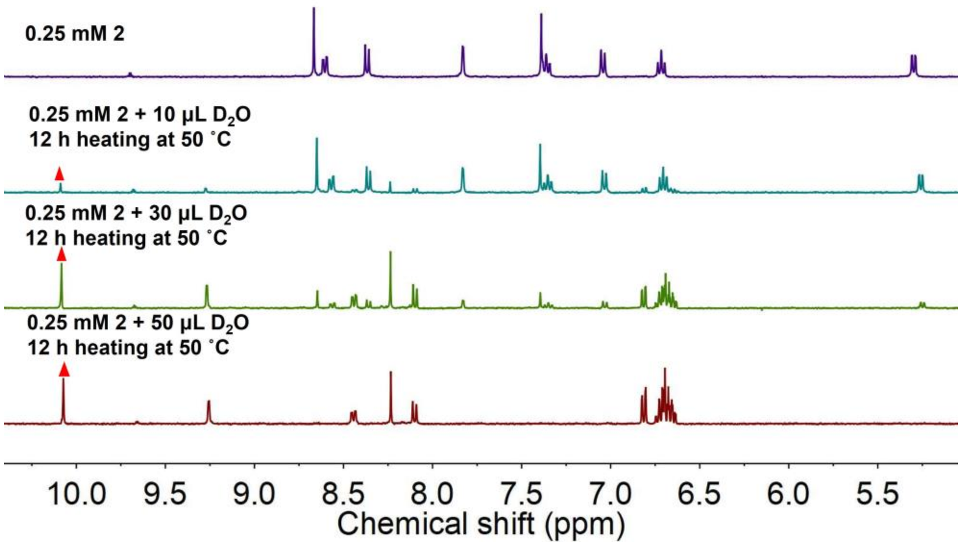

Supplementary Figure 68 | ^{1}H NMR spectra of the titration experiment of 0.25 mM **2** with different amounts of D_2O (400 MHz, CD_3CN, 298 K). The volume noted corresponds to the cumulative D_2O volume added to the solution. The aldehyde signal is marked with a red triangle. Full decomposition of **2** was observed following addition of 50 μL D_2O.

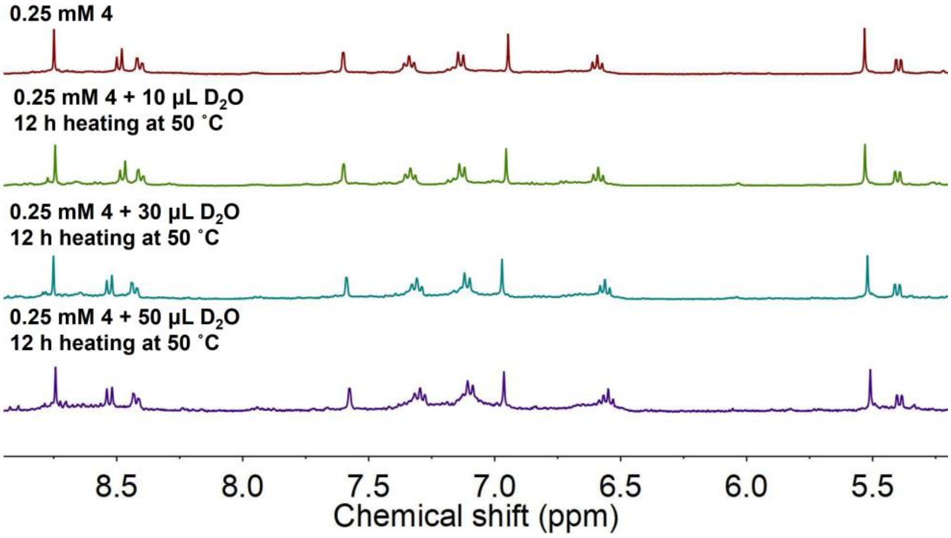

Supplementary Figure 69 | ^{1}H NMR spectra of the titration experiment of 0.25 mM **4** with different amounts of D_2O (400 MHz, CD_3CN, 298 K). The volume noted corresponds to the cumulative D_2O volume added to the solution. Most **4** remained intact following the addition of 50 μL D_2O.

5.4 Addition of HCl

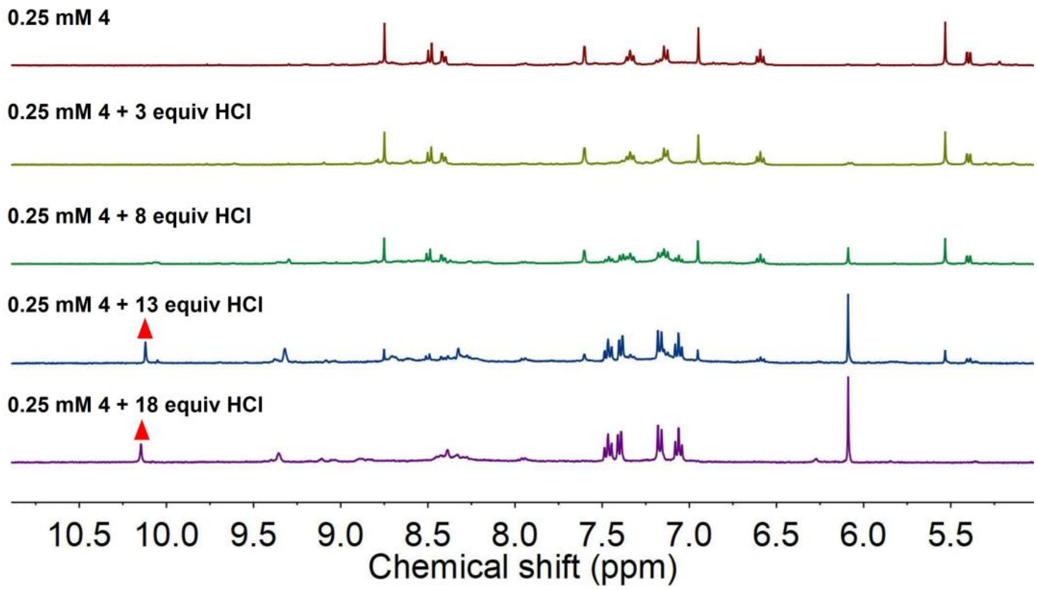

Supplementary Figure 70 | ^{1}H NMR spectra (400 MHz, CD_3CN, 298 K) of the titration experiment of 0.25 mM **4** with varying amounts of HCl (acetonitrile, 125 mM). The aldehyde signal is marked with a red triangle, and complete decomposition of **4** was observed following addition of 18 equiv HCl.

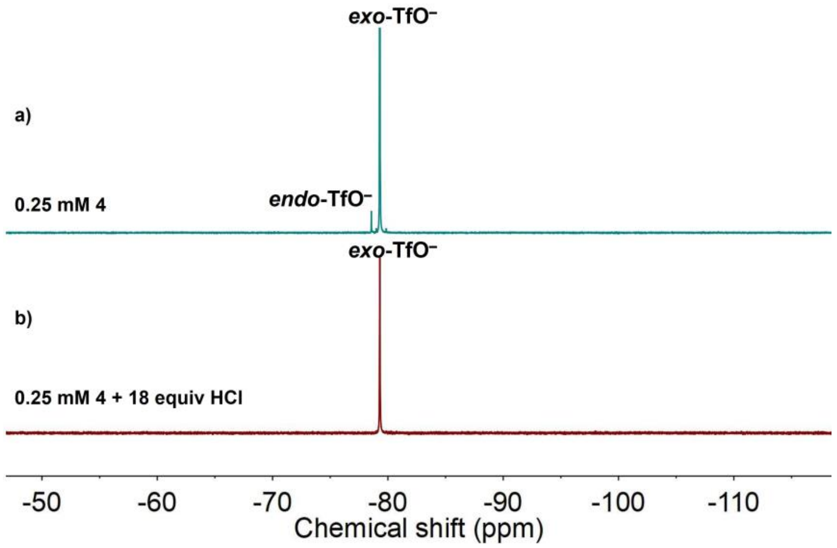

Supplementary Figure 71 | ^{19}F NMR spectra (298 K, 376 MHz, CD_3CN) of **a)** a 0.25 mM solution of **4**; **b)** the solution from **a)** with addition of 18 equiv HCl acetonitrile solution.

5.5 Addition of H_2O_2

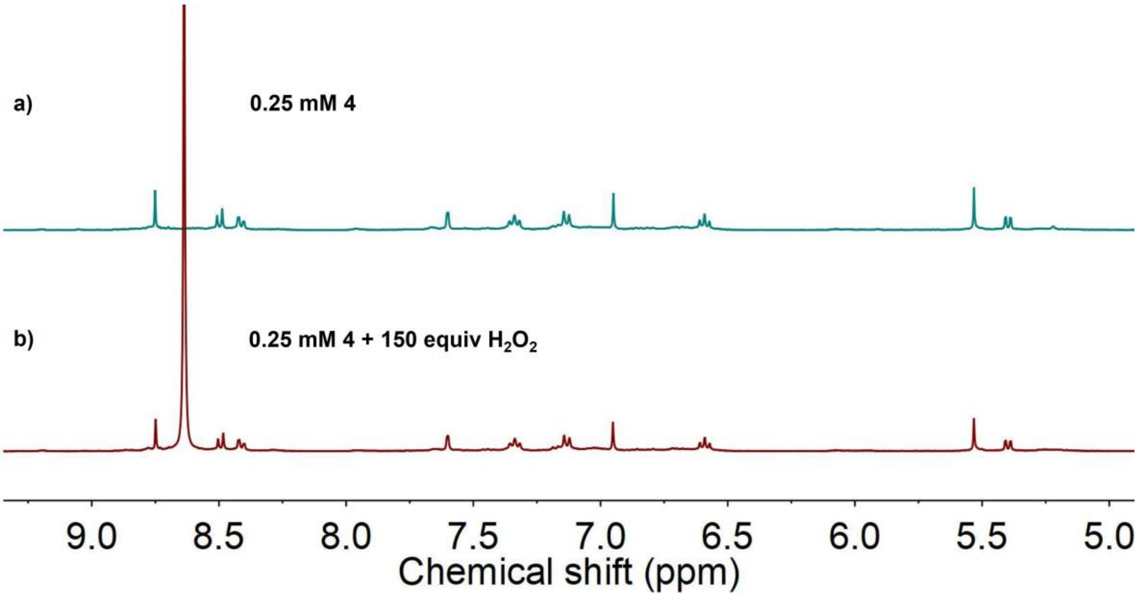

Supplementary Figure 72 | ^{1}H NMR spectra (400 MHz, CD_3CN, 298 K) of a) 0.25 mM **4**; b) 0.25 mM **4** with addition of 2 μL 30% H_2O_2 (aq) (~150 equiv), and no obvious decomposition of **4** was observed.

6. Reduction and demetallation of 3 and 4

6.1 Reduction and demetallation of **3**

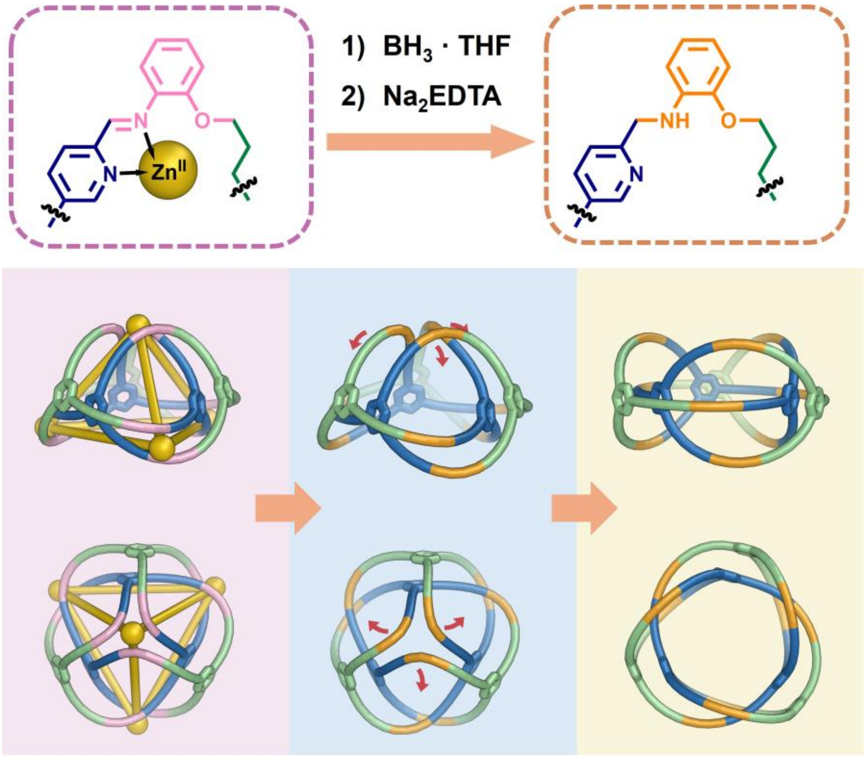

Supplementary Scheme 6 | Preparation of **5**

To a stirred solution of **3** (15 mg, 3.5 µmol, 1 equiv) in MeCN/MeOH (5/1, v/v, 2 mL total volume) at room temperature was added BH_3•THF (1 M, 400 µL, 400 µmol, 12 equiv./imine). The mixture was stirred for 2 hours during which the colour of the solution changed from orange to light yellow. CH_2Cl_2 (30 mL) and Na_2EDTA (10 mg) were then added and the mixture was stirred for 10 minutes. The resulting suspension was poured into 30 mL H_2O and the mixture was extracted with 10 × 5 mL CH_2Cl_2. The combined organic layers were filtered over cotton covered with sand then the solvent was removed under vacuum. The residue was washed with methanol three times (3 × 10 mL) and dried under vacuum affording the reduced and demetallated structure **5** (8.6 mg, 3.0 µmol, 87%).

^{1}H NMR (500 MHz, Methylene Chloride-*d*$_2$) δ 8.97 (d, J = 2.3 Hz, 3H), 8.11 (d, J = 2.4 Hz, 6H), 7.98 – 7.94 (m, 6H), 7.47 (d, J = 8.2 Hz, 3H), 7.32 (d, J = 1.6 Hz, 6H), 7.28 (dd, J = 8.1, 2.4 Hz, 6H), 7.14 (dd, J = 8.1, 2.4 Hz, 3H), 6.99 – 6.58 (m, 33H), 6.55 (d, J = 1.6 Hz, 3H), 5.57 (t, J = 5.3 Hz, 3H), 5.48 (t, J = 5.3 Hz, 6H), 5.35 (s, 3H), 5.12 (s, 6H), 4.52 (t, J = 5.0 Hz, 6H), 4.35 (t, J = 5.0 Hz, 12H), 4.09 – 3.93 (m, 18H), 3.45 – 3.42 (br, 6H), 3.32 – 3.19 (br, 12H), 2.10 – 1.56 (m, 36H).

^{13}C NMR (126 MHz, CD$_2$Cl$_2$) δ 160.1, 159.7, 157.7, 157.0, 146.9, 146.6, 146.5, 138.6, 138.3, 138.0, 134.9, 134.4, 134.0, 133.3, 130.1, 124.5, 122.8, 122.2, 121.9, 121.2, 119.6, 116.6, 116.6, 114.6, 111.4, 111.0, 110.3, 110.1, 110.0, 93.0, 92.5, 68.1, 67.9, 67.4, 67.1, 53.8, 53.6, 53.4, 53.2, 53.0, 48.6, 26.2, 26.0, 26.0, 25.8.

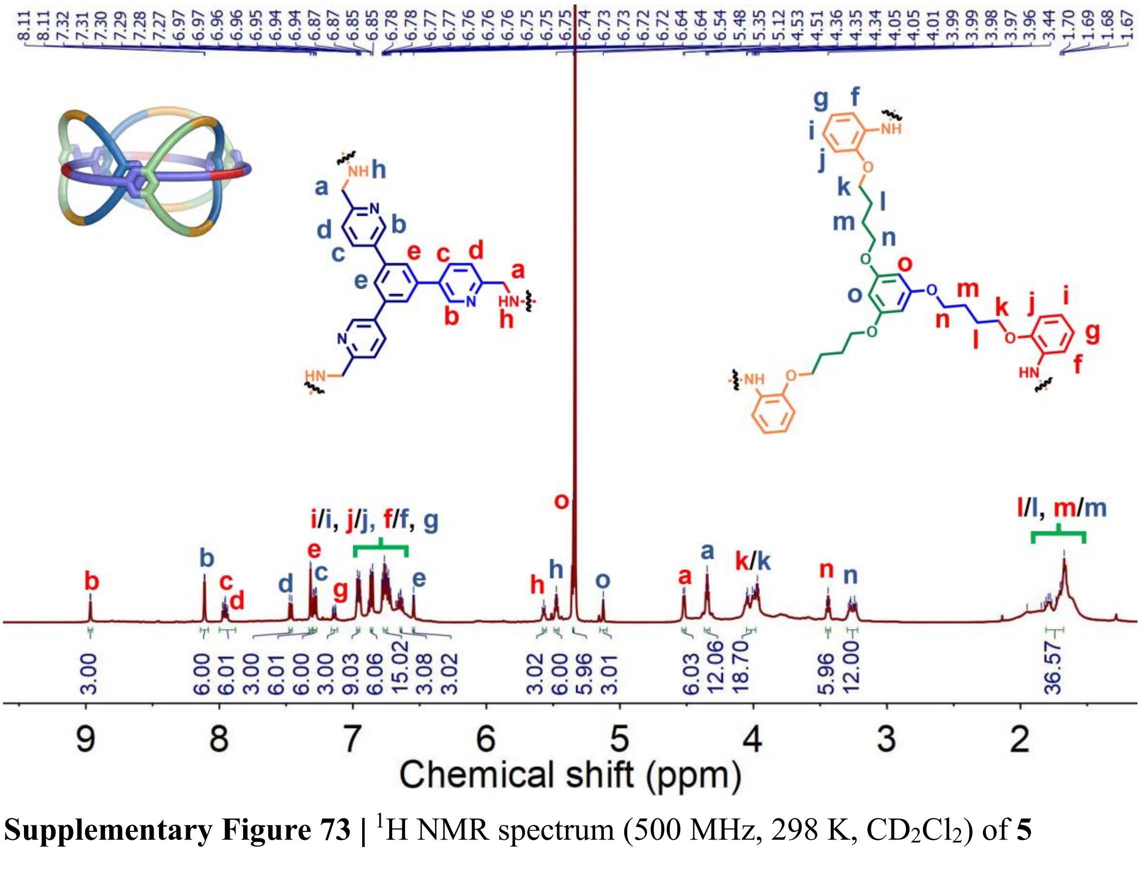

Supplementary Figure 73 | ^{1}H NMR spectrum (500 MHz, 298 K, CD$_2$Cl$_2$) of **5**

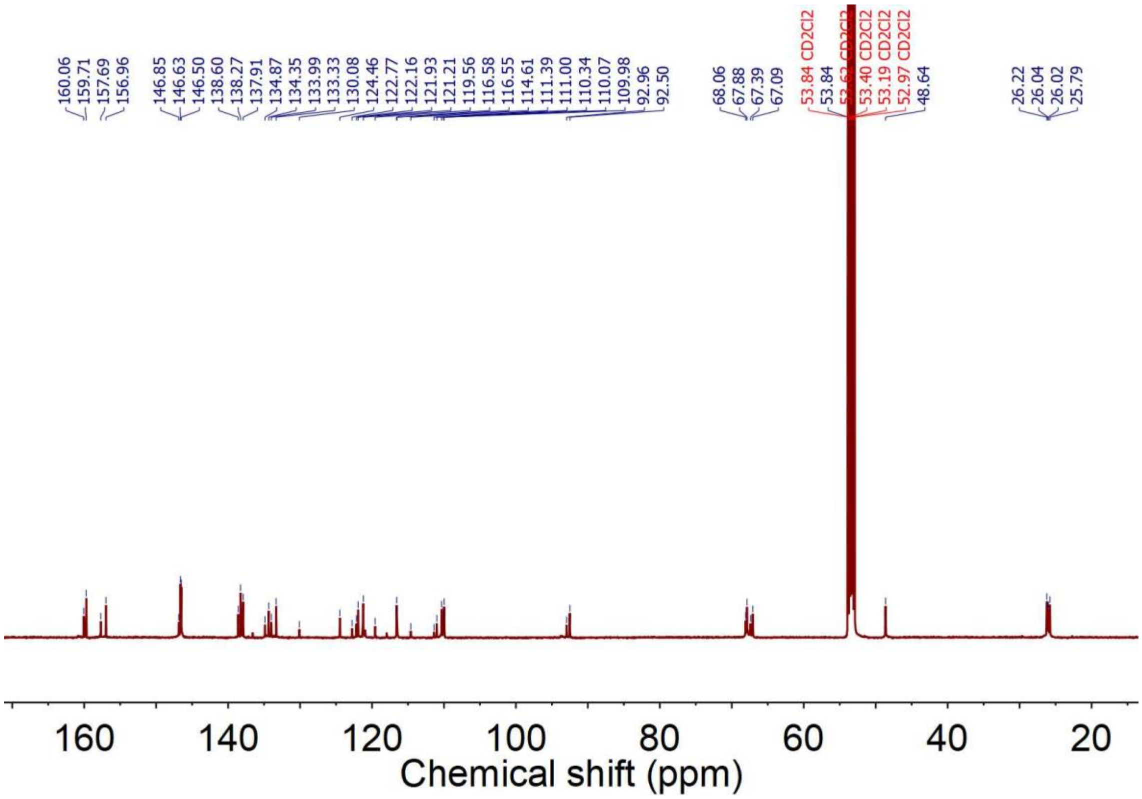

Supplementary Figure 74 | ^{13}C NMR spectrum (126 MHz, 298 K, CD_2Cl_2) of **5**

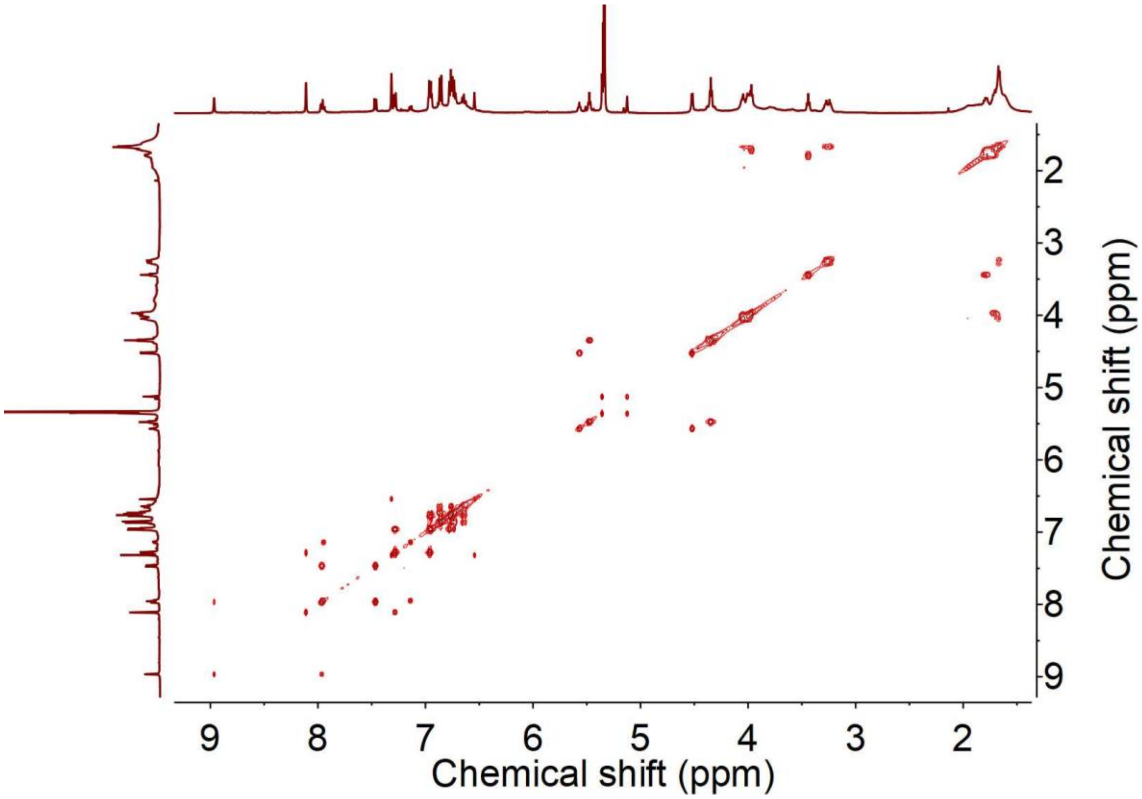

Supplementary Figure 75 | ^{1}H–^{1}H COSY spectrum (500 MHz, 298 K, CD_2Cl_2) of **5**.

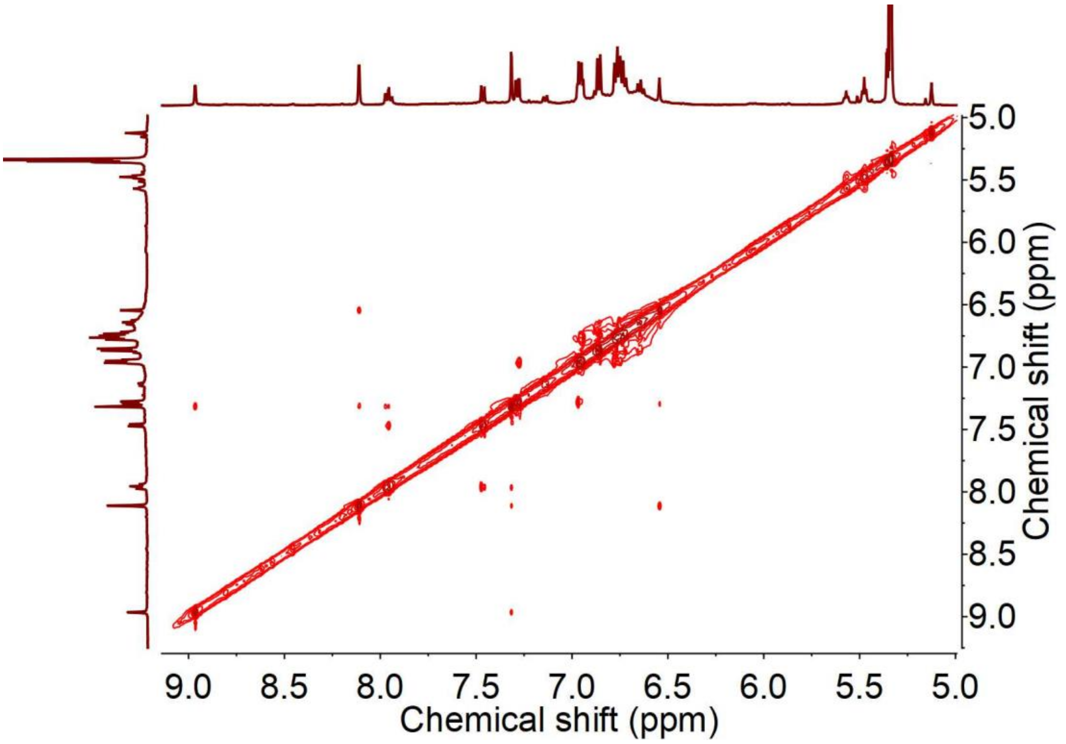

Supplementary Figure 76 | Partial ^{1}H–^{1}H NOESY spectrum (500 MHz, 298 K, CD_2Cl_2) of **5**.

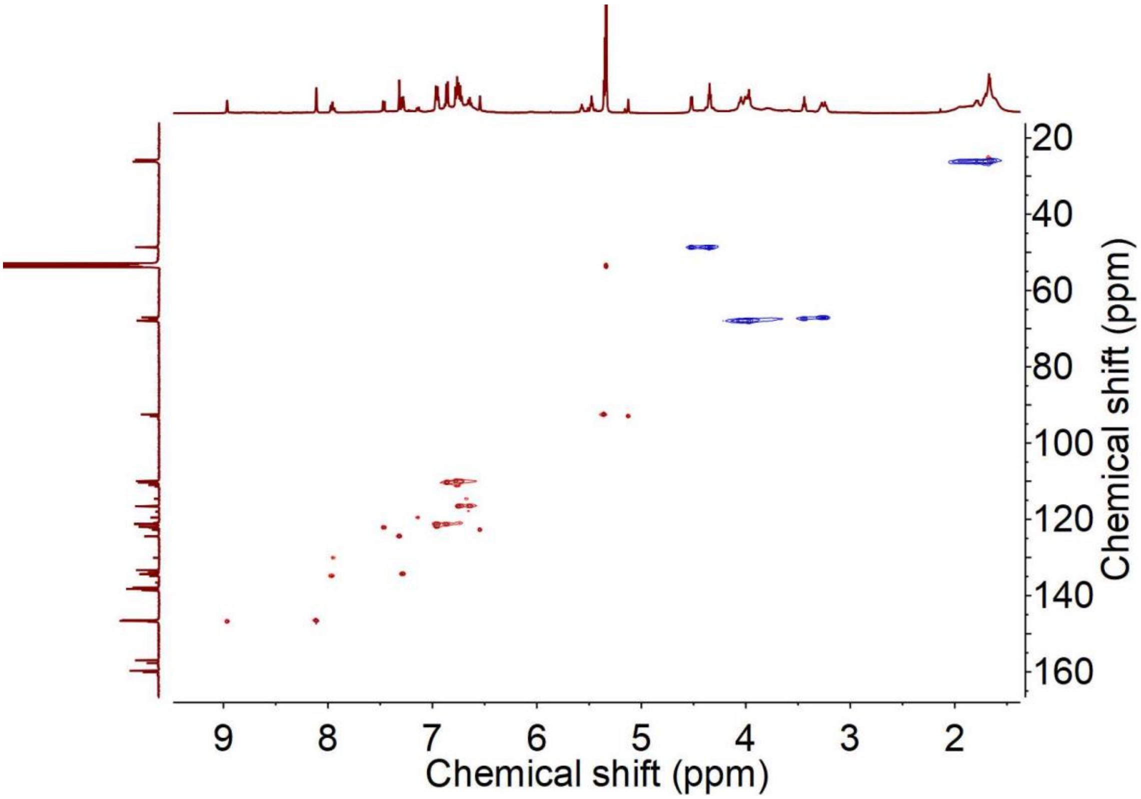

Supplementary Figure 77 | ^{1}H–^{13}C HSQC spectrum (500 MHz, 298 K, CD_2Cl_2) of **5**.

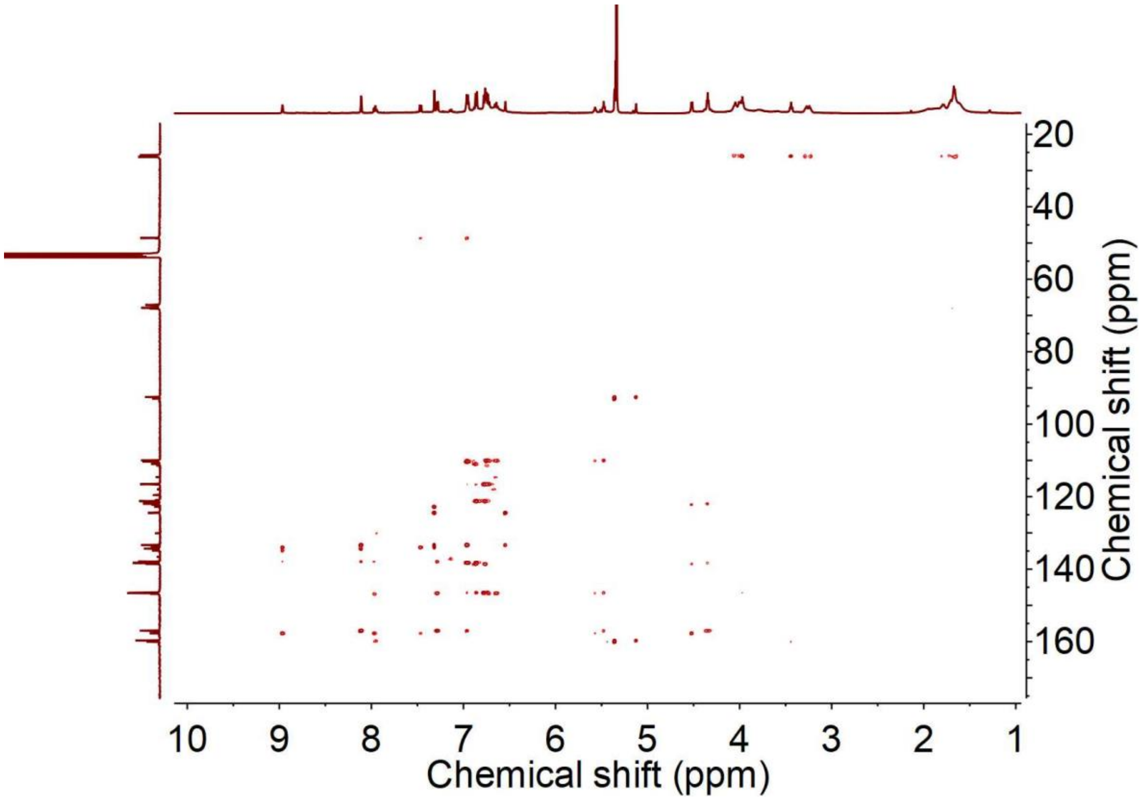

Supplementary Figure 78 | ^{1}H–^{13}C HMBC spectrum (500 MHz, 298 K, CD_2Cl_2) of **5**.

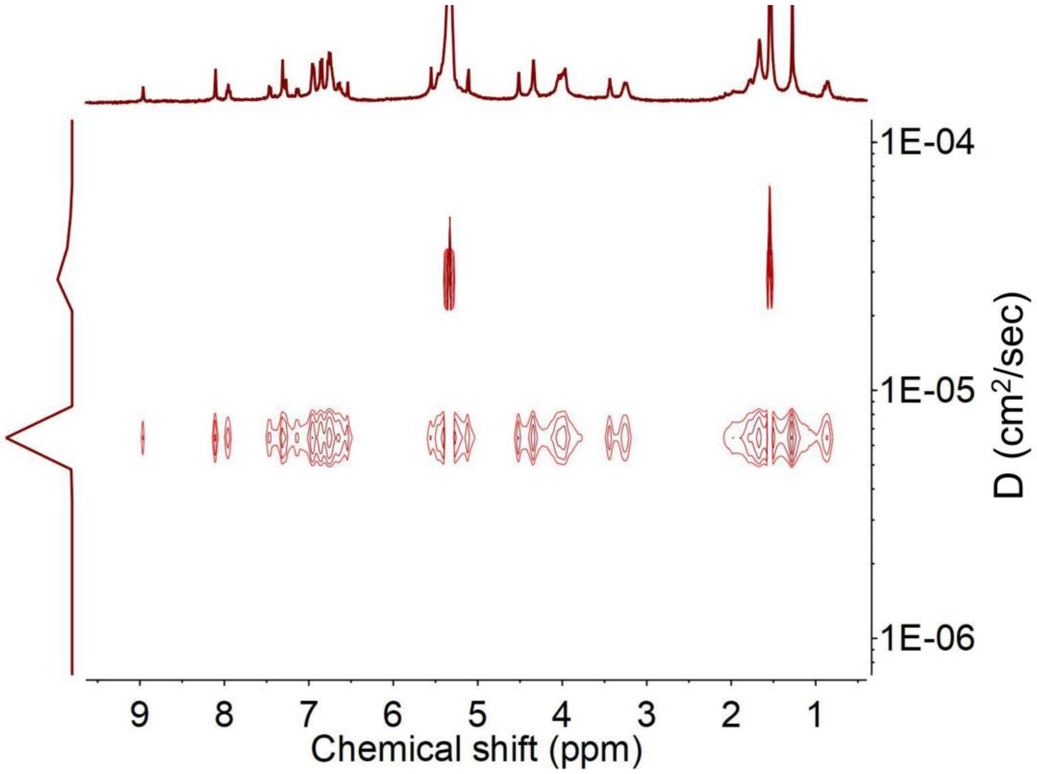

Supplementary Figure 79 | ^{1}H DOSY spectrum (400 MHz, 298K, CD_2Cl_2) of **5**. Diffusion coefficient: $D = 6.43 \times 10^{-10}$ m^2 s^{-1}, with radius of = 8.4 Å.

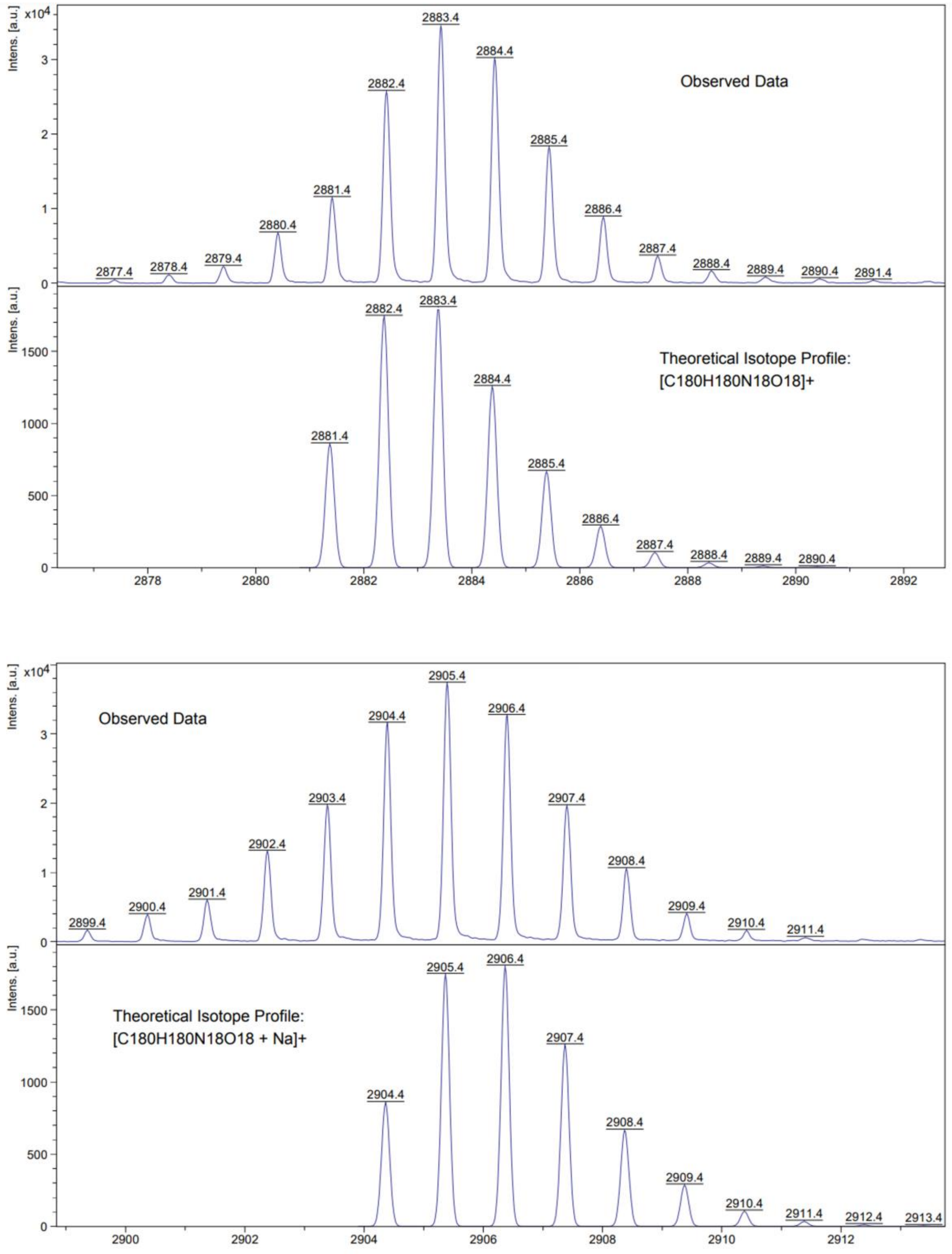

Supplementary Figure 80 | MALDI-TOF MS spectra of **5**.

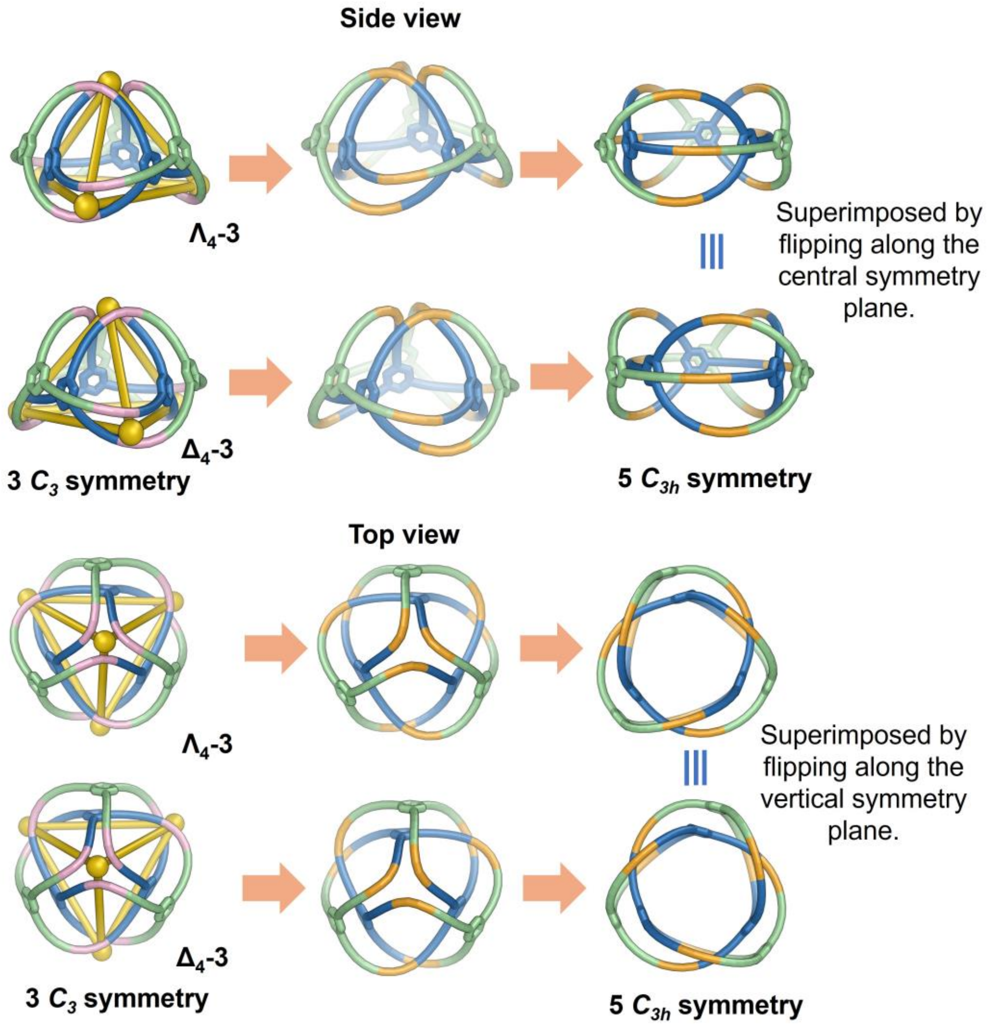

Supplementary Figure 81 | Cartoon illustration of the elimination of chirality during the transformation from **3** to **5**.

6.2 Reduction and demetallation of **4**

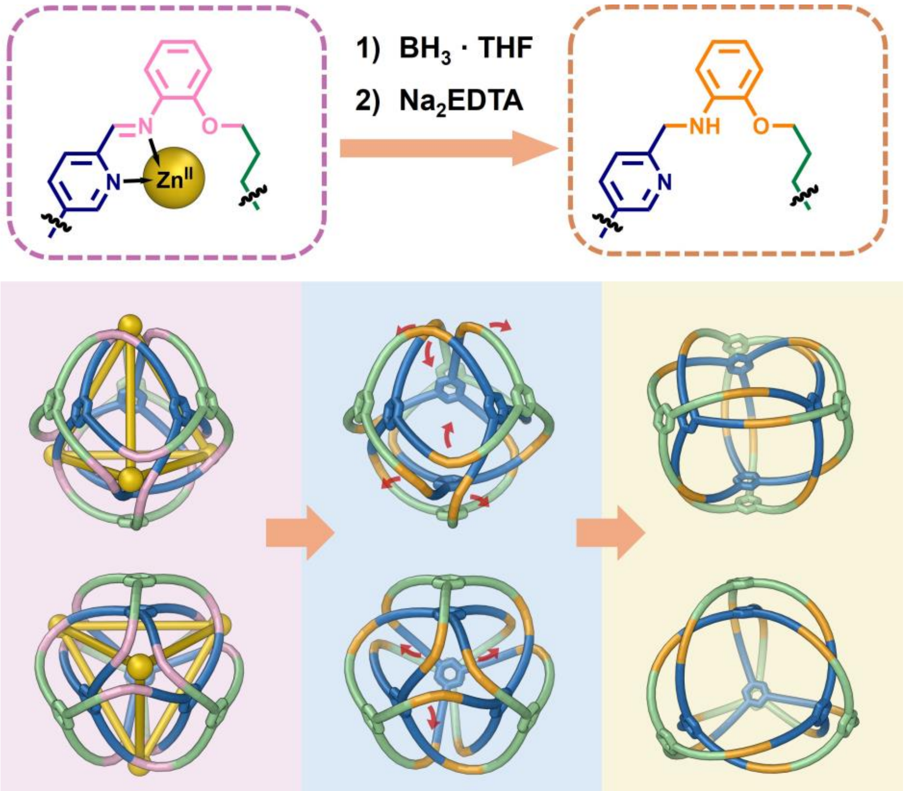

Supplementary Scheme 7 | Preparation of **6**

To a stirred solution of **4** (20 mg, 3.8 µmol, 1 equiv) in MeCN/MeOH (5/1, v/v, 2 mL total volume) at room temperature was added BH_3•THF (1 M, 500 µL, 500 µmol, 11 equiv/imine). The mixture was stirred for 2 hours during which the colour of the solution changed from yellow to light yellow. CH_2Cl_2 (30 mL) and Na_2EDTA (10 mg) were then added and the mixture was stirred for 10 minutes. The resulting suspension was poured into 30 mL H_2O and the mixture was extracted with 10 × 5 mL CH_2Cl_2. The combined organic layers were filtered over cotton covered with sand then the solvent was removed under vacuum. The residue was washed with methanol three times (3 × 10 mL) and dried under vacuum affording the reduced and demetallated structure **6** (8.0 mg, 2.1 µmol, 55%).

^{1}H NMR (500 MHz, Methylene Chloride-*d₂*) δ 8.45 (d, J = 2.2 Hz, 12H), 7.50 (dd, J = 8.1, 2.3 Hz, 12H), 7.22 (s, 12H), 7.19 (d, J = 8.1 Hz, 12H), 6.86 (td, J = 7.7, 1.3 Hz, 12H), 6.82 (dd, J = 8.1, 1.4 Hz, 12H), 6.69 (td, J = 7.7, 1.5 Hz, 12H), 6.50 (dd, J = 7.9, 1.5 Hz, 12H), 5.44 (s, 12H), 4.30 (tt, J = 15.3, 8.9 Hz, 24H), 3.98 (br, J = 8.8 Hz, 24H), 3.52 – 3.34 (br, 24H), 1.72-1.64 (m, J = 7.2, 5.1 Hz, 48H).

^{13}C NMR (126 MHz, CD_2Cl_2) δ160.0, 157.9, 146.8, 146.4, 138.5, 138.2, 134.5, 133.6, 124.1, 121.8, 121.3, 116.6, 110.5, 110.2, 92.9, 68.0, 67.4, 53.8, 53.6, 53.4, 53.2, 53.0, 48.7, 26.2, 26.0.

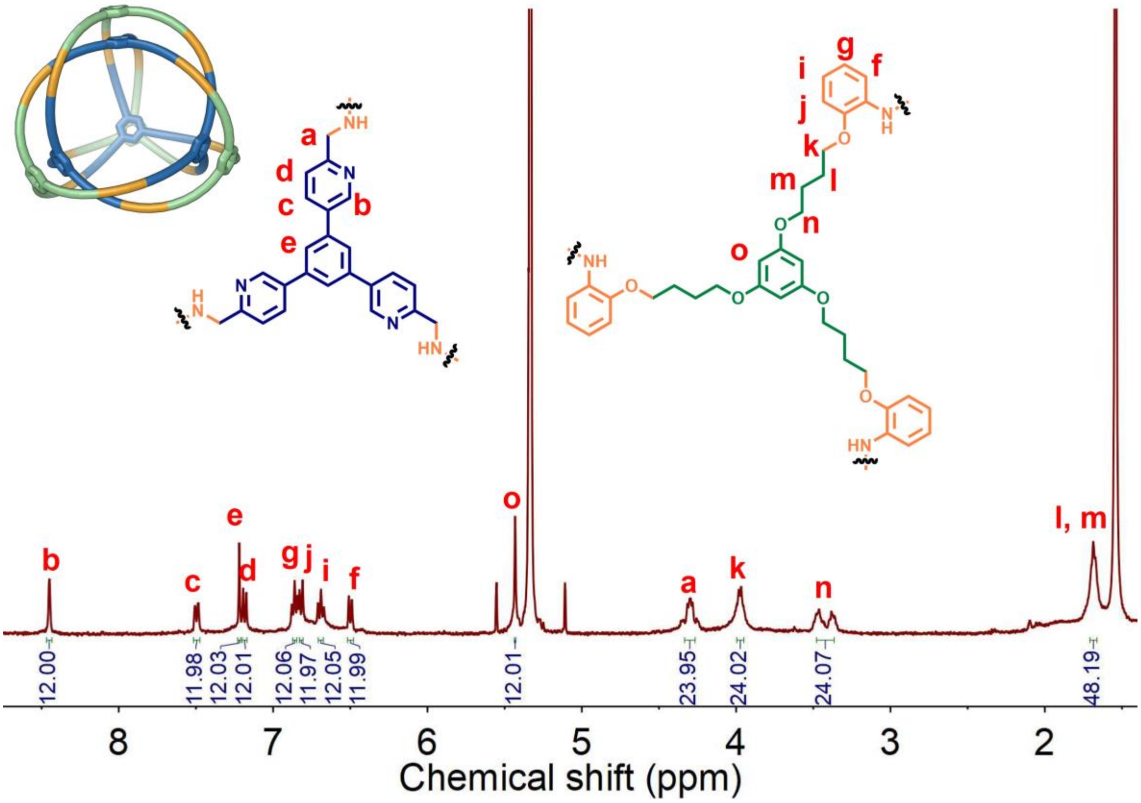

Supplementary Figure 82 | ^{1}H NMR spectrum (500 MHz, 298 K, CD_2Cl_2) of **6**

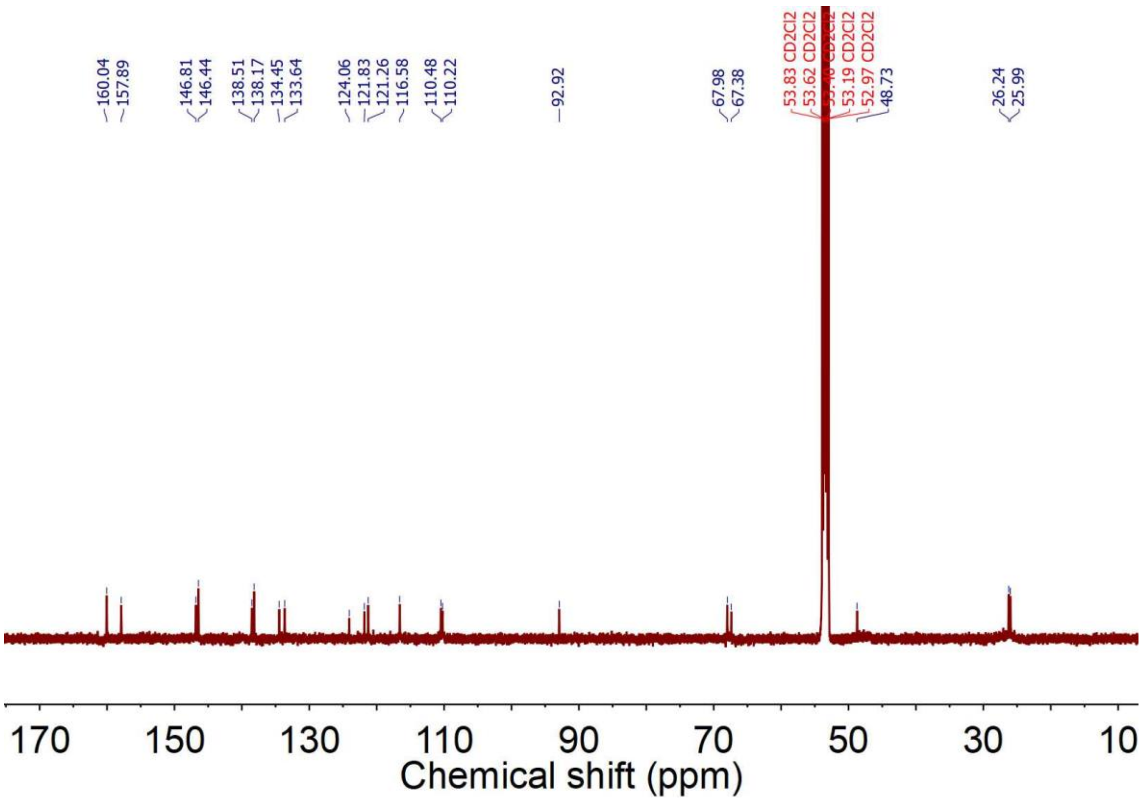

Supplementary Figure 83 | ^{13}C NMR spectrum (126 MHz, 298 K, CD_2Cl_2) of **6**

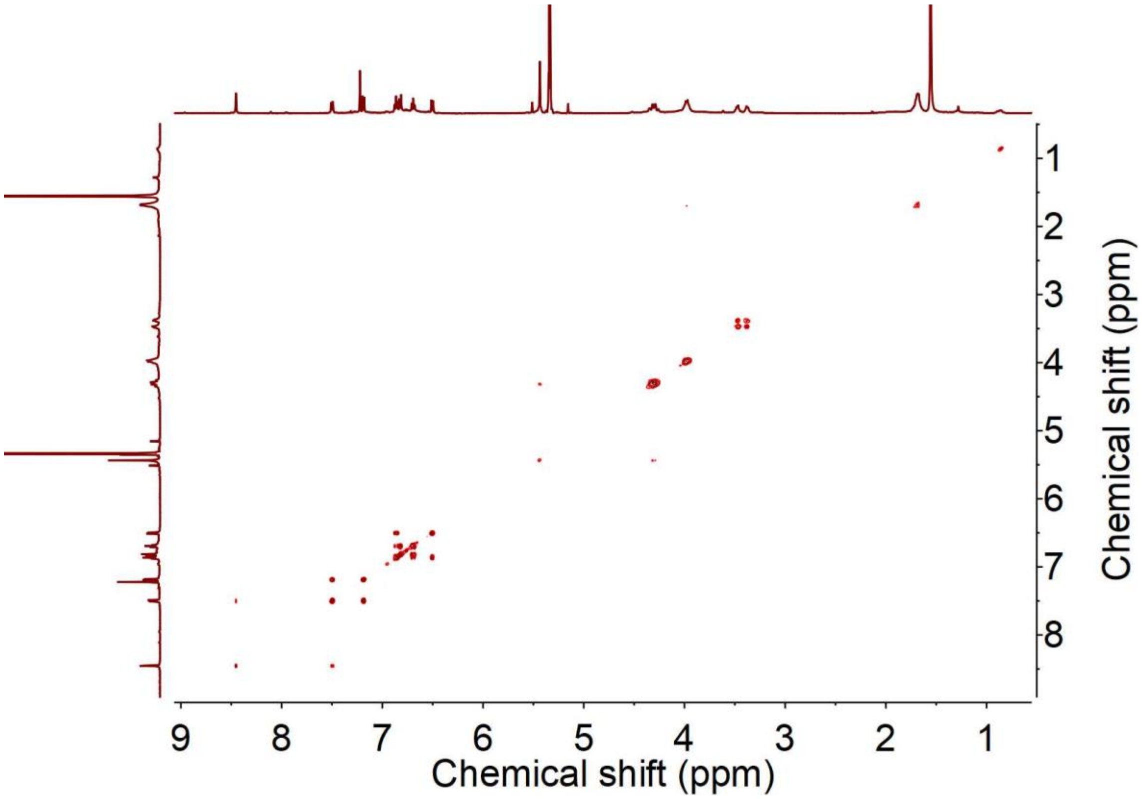

Supplementary Figure 84 | ^{1}H–^{1}H COSY spectrum (500 MHz, 298 K, CD_2Cl_2) of **6**.

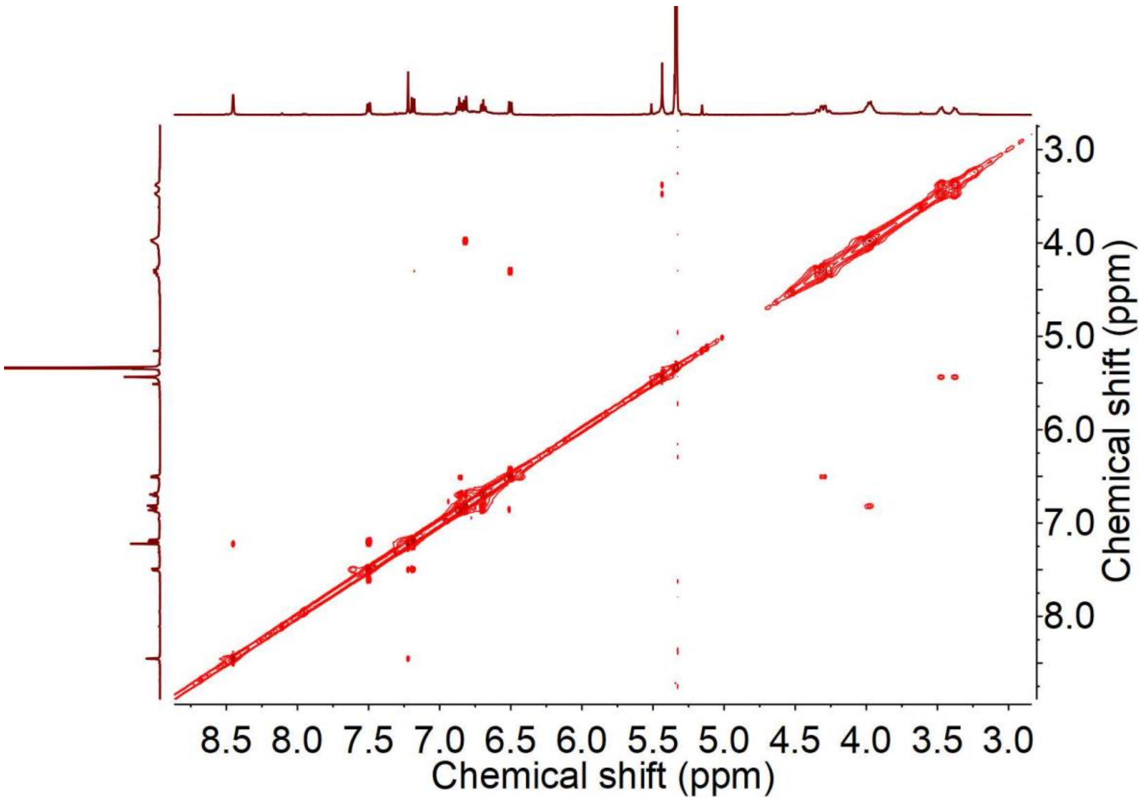

Supplementary Figure 85 | $^{1}H–^{1}H$ NOESY spectrum (500 MHz, 298 K, CD_2Cl_2) of **6**.

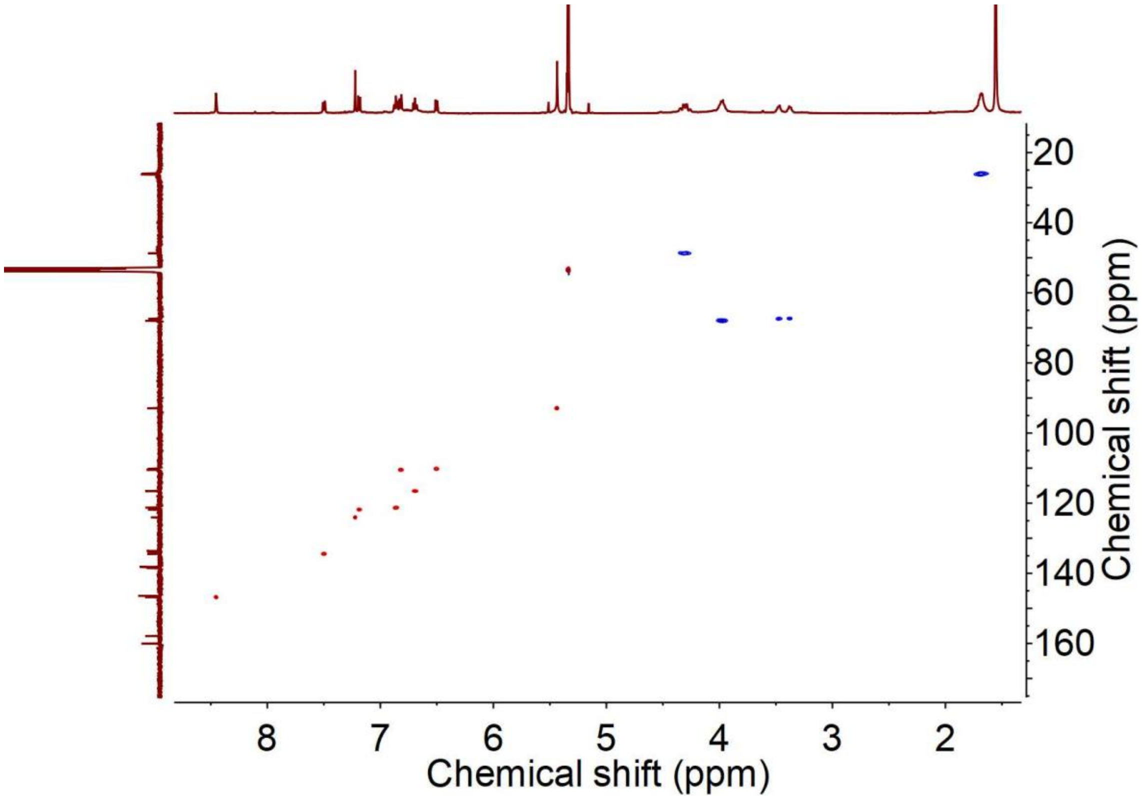

Supplementary Figure 86 | $^{1}H–^{13}C$ HSQC spectrum (500 MHz, 298 K, CD_2Cl_2) of **6**.

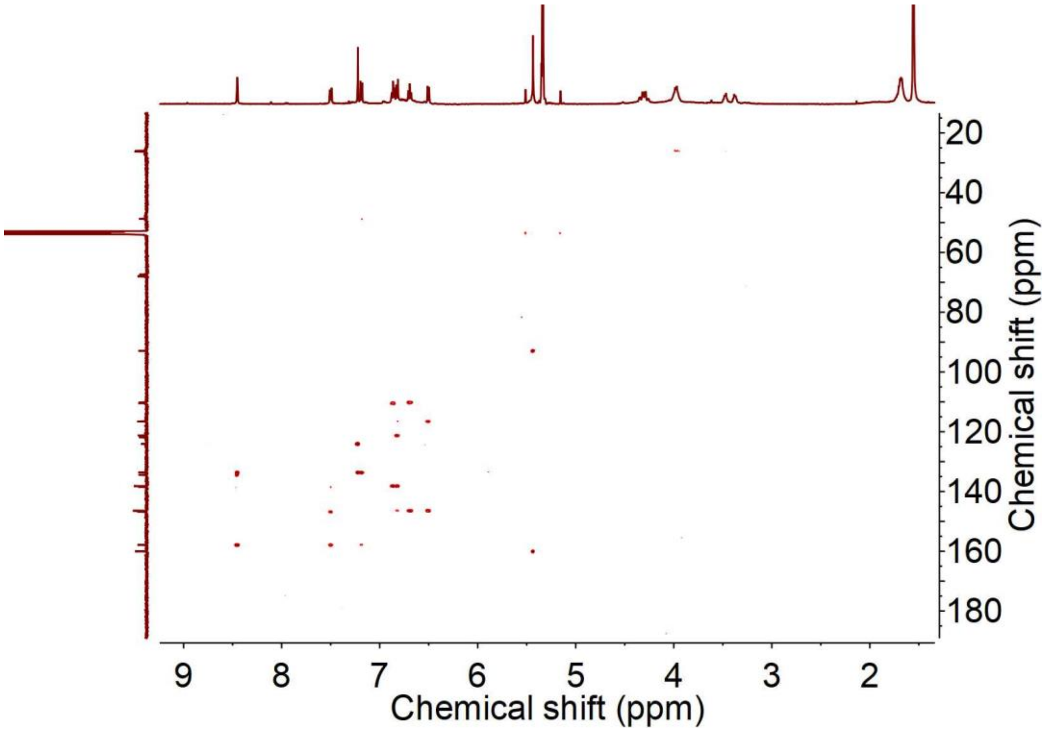

Supplementary Figure 87 | ^{1}H–^{13}C HMBC spectrum (500 MHz, 298 K, CD_2Cl_2) of **6**.

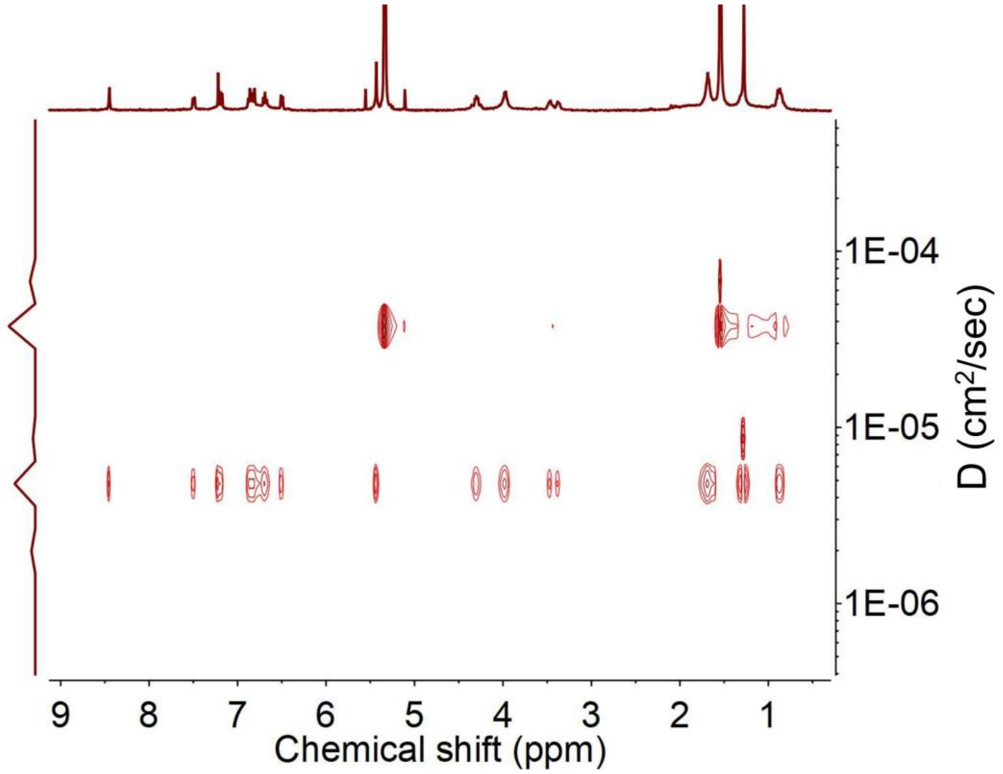

Supplementary Figure 88 | ^{1}H DOSY spectrum (400 MHz, 298 K, CD_2Cl_2) of **6**. Diffusion coefficient: D = 4.80×10^{-10} m^2 s^{-1}, with radius of = 11.2 Å.

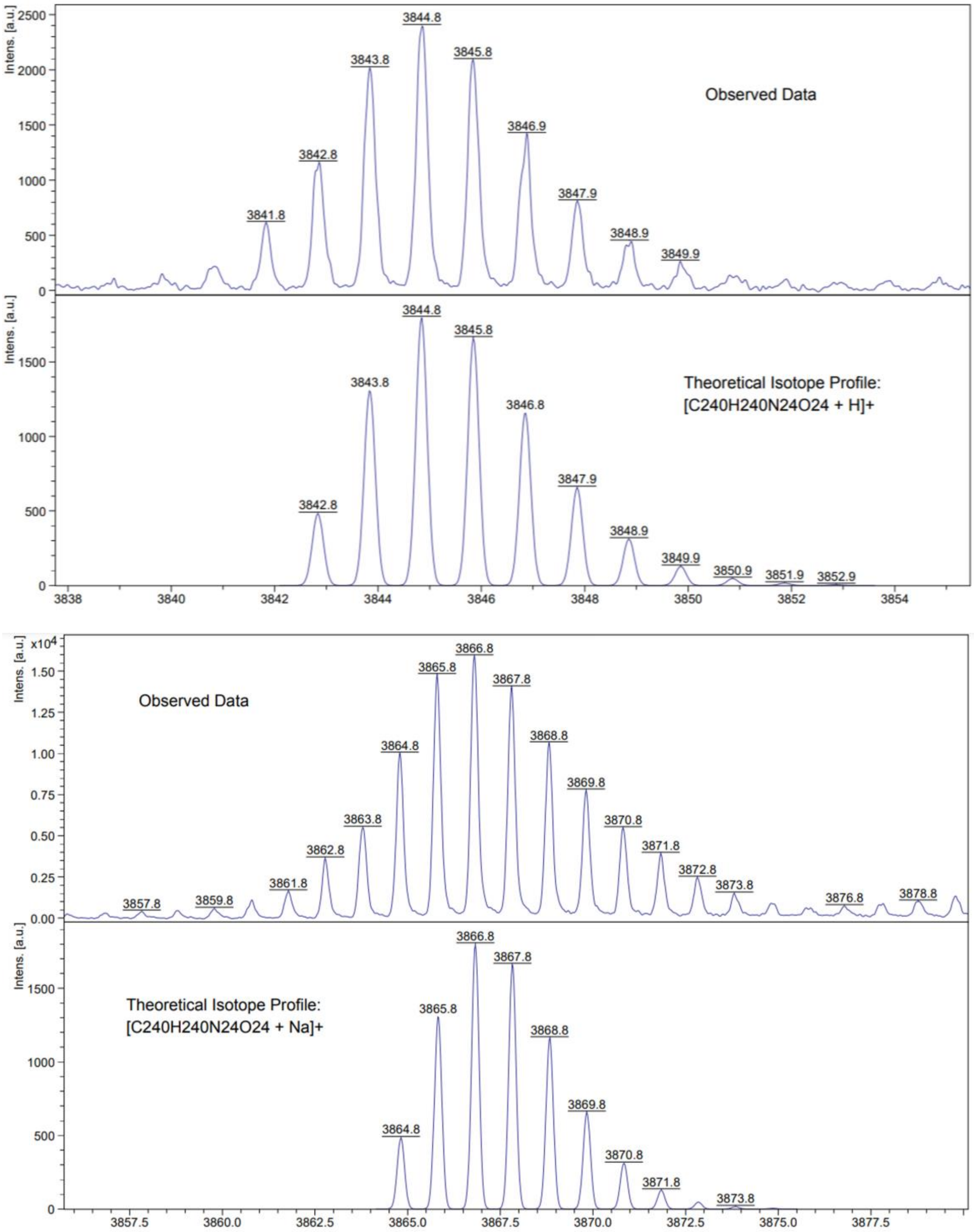

Supplementary Figure 89 | MALDI-TOF MS spectra of **6**.

7. X-ray Crystallography

Data were collected at Beamline I19 of Diamond Light Source employing silicon double crystal monochromated synchrotron radiation (0.6889 Å, Dectris PILATUS 2M detector) with ω and ψ scans at 100(2) K.[3] Data integration and reduction were undertaken with Xia2.[4–6] Subsequent computations were carried out using the WinGX-32[7] and ShelXle[8] graphical user interfaces. Empirical absorption corrections were applied to the data using DIALS.[6] The structures were solved by direct methods using SHELXT[9] then refined and extended with SHELXL.[10] In general, non-hydrogen atoms with occupancies greater than 0.5 were refined anisotropically. Carbon-bound hydrogen atoms were included in idealised positions and refined using a riding model. Disorder was modelled using standard crystallographic methods including constraints, restraints and rigid bodies where necessary. Crystallographic data along with specific details pertaining to the refinement follow. Crystallographic data have been deposited with the CCDC (deposition numbers 2415403 for **3** and 2415402 for **4**).

Complex 3

Formula $C_{186.75}H_{164.62}Cl_3F_{15}N_{18.88}O_{33}S_5Zn_4$, *M* 4014.37, Triclinic, space group P-1 (#2), *a* 22.5654(3), *b* 30.5603(6), *c* 37.5780(6) Å, α 107.488(2), β 92.9670(10), γ 92.877(2)°, *V* 24624.2(8) Å^3, D_c 1.083 g cm^{-3}, *Z* 4, crystal size 0.035 by 0.030 by 0.020 mm, colour yellow, habit block, temperature 100(2) Kelvin, λ(Synchrotron) 0.6889 Å, μ(Synchrotron) 0.485 mm^{-1}, T(Analytical)$_{min,max}$ 0.9323095873654196, 1.0, $2\theta_{max}$ 31.99, *hkl* range -18 18, -24 24, -30 30, *N* 44861, N_{ind} 25178(R_{merge} 0.0687), N_{obs} 10107(I > 2σ(I)), N_{var} 3724, residuals* $R1(F)$ 0.1269, $wR2(F^2)$ 0.3785, GoF(all) 0.951, $\Delta\rho_{min,max}$ -0.407, 0.902 e$^-$ Å^{-3}.

*$R1 = \Sigma||F_o| - |F_c||/\Sigma|F_o|$ for $F_o > 2\sigma(F_o)$; $wR2 = (\Sigma w(F_o^2 - F_c^2)^2/\Sigma(wF_c^2)^2)^{1/2}$ all reflections

$w=1/[\sigma^2(F_o^2)+(0.2497P)^2]$ where $P=(F_o^2+2F_c^2)/3$

Specific refinement details:

The crystals of **3** with composition $[Zn_4LCl_3]\cdot 5OTf\cdot 0.875CH_3CN$ [+ solvent] were grown by diffusion of diethyl ether into an acetonitrile solution of the **3**·8OTf complex containing a small amount of chloride ions. The trace amounts of chloride required to obtain X-ray quality crystals results in a chloride anion coordinated to each of the bis-(pyridylimine)-chelated Zn^{II} vertices. We note the possibility of coordination of triflate, acetonitrile or water to these sites in solution.

The crystals employed were the best obtained from many attempts. The crystals were very weakly diffracting, immediately losing solvent upon removal from the mother liquor. Despite rapid handling prior to flash cooling in liquid nitrogen and the use of synchrotron radiation, few reflections at greater than 1.25 Å resolution were observed, and the data were trimmed accordingly. The diffraction was broad with high mosaicity and the quality of the integration is less than ideal hence the values of the R1 and wR2 factors are larger than for typical small molecule structures. Furthermore the crystals suffered beam damage from the synchrotron radiation and the completeness is only 95.3%. Nevertheless, the quality of the data is sufficient to establish the connectivity of the structure. The asymmetric unit was found to contain two complete **3** assemblies and associated counterions and solvent molecules.

In order to compensate for the limited resolution of the data a substantial number of restraints were applied during refinement. To obtain a reasonable model for the organic parts of the structure the GRADE program[11] was employed using the GRADE Web Server[12] to generate a full set of bond distance and angle restraints (DFIX, DANG, FLAT) for the flexible outer ligands. For the more rigid inner ligands, bond lengths and angles within pairs of chemically identical organic ligands were restrained to be similar to each other. The thermal parameters are high throughout the structure due to high thermal motion, the less than ideal resolution and generally poor diffraction properties of the crystals. Thermal parameter restraints (SIMU, RIGU for anisotropic atoms) were applied to all atoms except for zinc. The flexible outer ligands were refined isotropically.

The anions within the structure show evidence of substantial disorder. Three of the located triflate anions were modelled as disordered over two locations. Substantial bond length and thermal parameter restraints were applied to facilitate stable refinement of the anions and most low occupancy anions were modelled with isotropic thermal parameters. The occupancies of all located anions were allowed to freely refine which resulted in a

discrepancy of ca. 2.8 anions per Zn_4LCl_3 assembly (5.6 per asymmetric unit). Most of the remaining electron density peaks (following the use of SQUEEZE, see below) are close to the triflate anions suggesting further disorder which could not be resolved due to the limited resolution of the data. The hydrogen atoms of the acetonitrile molecules could not be located in the electron density map and were therefore not included in the model.

Further reflecting the solvent loss and poor diffraction properties there is a significant amount of void volume in the lattice containing smeared electron density from disordered solvent and the remaining anions. Consequently, the SQUEEZE[13] function of PLATON[14] was employed to remove the contribution of the electron density associated with these remaining anions and further highly disordered solvent, which gave a potential solvent accessible void of 8867 $Å^3$ per unit cell (a total of approximately 2839 electrons). Diffuse solvent molecules could not be assigned to acetonitrile or diethyl ether and were therefore not included in the formula. Consequently, the molecular weight and density given above are underestimated.

CheckCIF gives four A and six B level alerts. These alerts mostly result from the limited resolution of the data and poor diffraction properties of the crystals (low sine(theta_max)/wavelength, poor data / parameter ratio, low completeness, low bond precision, high wR2 value, isotropic modelling) as described above. Four alerts result from short contacts between hydrogens of the flexible alkyl chains and a neighbouring phenyl ring proton. These contacts may be a feature of the structure or arise from the very high level of thermal motion in the alkyl chains resulting in the hydrogen positions not being very precisely determined.

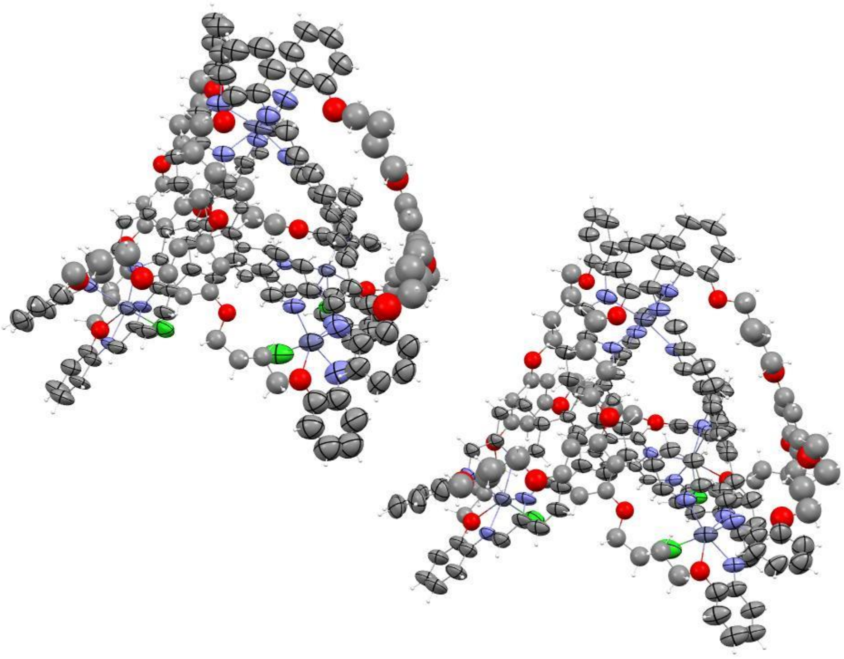

Supplementary Figure 90 | ORTEP plot of the cationic part of the crystal structure of **3** with composition $[Zn_4LCl_3]\cdot 5OTf\cdot 0.875CH_3CN$, showing thermal ellipsoids at 30%. Disorder and solvent molecules are omitted for clarity.

Complex 4

Formula $C_{257}H_{232.50}F_{24}N_{27}O_{48.75}S_8Zn_4$, *M* 5453.15, Orthorhombic, space group P 2_1 2_1 2_1 (#19), *a* 22.62310(10), *b* 28.63830(10), *c* 42.5374(2) Å, *V* 27559.4(2) Å^3, D_c 1.314 g cm^{-3}, *Z* 4, crystal size 0.040 by 0.030 by 0.030 mm, colour yellow, habit block, temperature 100(2) Kelvin, λ(Synchrotron) 0.6889 Å, μ(Synchrotron) 0.454 mm^{-1}, T(Analytical)$_{min,max}$ 0.8451548134040332, 1.0, $2\theta_{max}$ 47.81, *hkl* range -26 26, -31 32, -50 50, *N* 196072, N_{ind} 46126(R_{merge} 0.0464), N_{obs} 20720(I > 2σ(I)), N_{var} 3257, residuals* $R1(F)$ 0.0877, $wR2(F^2)$ 0.2302, GoF(all) 1.006, $\Delta\rho_{min,max}$ -0.422, 0.662 e$^-$ Å^{-3}.

*$R1 = \Sigma||F_o| - |F_c||/\Sigma|F_o|$ for $F_o > 2\sigma(F_o)$; $wR2 = (\Sigma w(F_o^2 - F_c^2)^2/\Sigma(wF_c^2)^2)^{1/2}$ all reflections,

$w=1/[\sigma^2(F_o^2)+(0.121P)^2]$ where $P=(F_o^2+2F_c^2)/3$

Specific refinement details:

The crystals of **4** with composition $[Zn_4L]\cdot 8OTf\cdot 3CH_3CN\cdot 0.75C_4H_{10}O$ [+ solvent] were grown by diffusion of diethyl ether into an acetonitrile solution of the complex. The crystals employed immediately lost solvent after removal from the mother liquor. However rapid handling prior to flash cooling in liquid nitrogen and the use of synchrotron radiation enabled the collection of data to around 0.85 Å resolution. The asymmetric unit was found to contain one complete Zn_4L assembly and associated counterions and solvent molecules. The structure was refined as a racemic twin with the Flack parameter refining to 0.149(16).

Due to the high degree of thermal motion within the structure bond lengths and angles within pairs of chemically identical organic ligands were restrained to be similar to each other. Additional DFIX and restraints were applied to some of the more flexible regions of the structure. Thermal parameter restraints (SIMU, RIGU) were applied to all atoms except for zinc. However, even with these restraints some thermal parameters remain higher than ideal, especially for the flexible outer ligands which display evidence of dynamic disorder. Attempts to model disorder of these ligands over multiple discrete positions were not successful.

The anions within the structure show evidence of substantial disorder. Six of the located triflate anions were modelled as disordered over two locations. Substantial bond length and thermal parameter restraints were applied to facilitate stable refinement of the anions and most low occupancy anions were modelled with isotropic thermal parameters. The occupancies of all located anions were allowed to freely refine which resulted in a discrepancy of two anions per Zn_4L assembly. The hydrogen atoms of some low occupancy acetonitrile molecules could not be located in the electron density map and were therefore not included in the model.

Further reflecting the solvent loss and poor diffraction properties, there is a significant amount of void volume in the lattice containing smeared electron density from disordered solvent and the remaining anions. Consequently, the SQUEEZE[13] function of PLATON[14] was employed to remove the contribution of the electron density associated with these remaining anions and further highly disordered solvent, which gave a potential solvent accessible void of 2878 Å^3 per unit cell (a total of approximately 1001 electrons). Diffuse solvent molecules could not be assigned to acetonitrile or diethyl ether and were therefore not included in the formula. Consequently, the molecular weight and density given above are underestimated.

CheckCIF gives three B level alerts. One B level alert for low bond precision of the C-C bonds results from the significant amount of thermal motion present within the structure, especially around the flexible outer ligands. The other alerts all result from short contacts involving hydrogens of the flexible alkyl chains. They may be genuine features of the structure or due the large amount of thermal motion (or dynamic disorder) in these sections of the structure resulting in hydrogen positions not being very precisely determined.

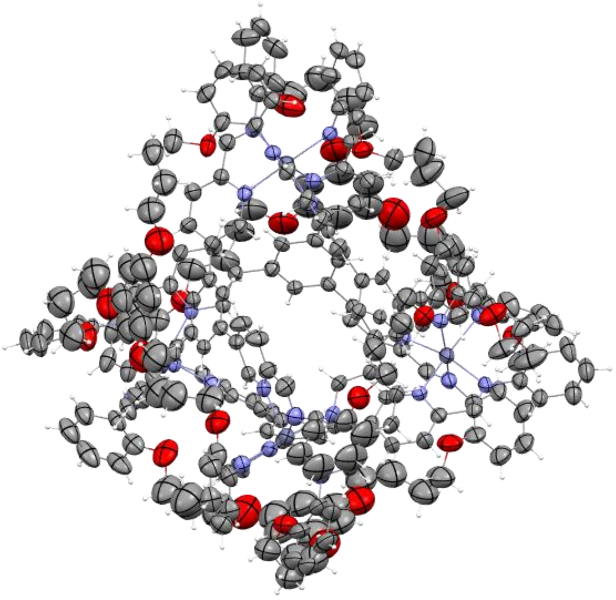

Supplementary Figure 91 | ORTEP plot of the cationic part of the crystal structure of **4** with composition $[Zn_4L]\cdot 8OTf\cdot 3CH_3CN\cdot 0.75C_4H_{10}O$, showing thermal ellipsoids at 30%. Disorder and solvent molecules are omitted for clarity.

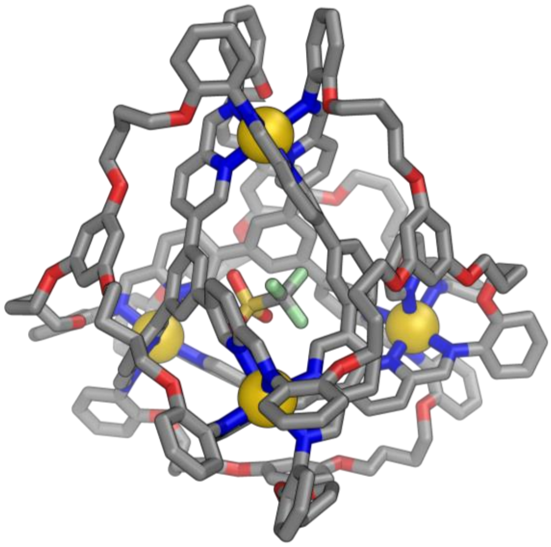

Supplementary Figure 92 | Crystal structure of $TfO^{-}\subset$**4**. (Zn: yellow, N: blue, O: red, C: gray, F: green). Disorder, hydrogen atoms, non-encapsulated counterions and solvents are omitted for clarity.

8. Calculations

8.1 General procedure and discussions

Quantum mechanical calculations were carried out on structures **1, 2, 5** and **6** using a combination of different methods to find their global minima. Local geometry optimizations were performed using the OPTIM program[15,16], which contains a wide variety of geometry optimization tools for locating stationary points on potential energy surfaces and calculating reaction pathways. Basin-hopping[17,18] (BH) global optimizations were performed using the GMIN program[19], which contains tools for finding global minima and calculating thermodynamic properties from basin-sampling. We note that basin-hopping has previously produced knotted global minima for clusters of dipolar particles[20]. For both programs, an interface to the *xtb* program was used to calculate the energy and gradient at the GFN-FF[21] and GFN2-xTB[22,23] levels of theory. Input structures for these calculations were produced using the MM3 [24–26] force field in SCIGRESS software[27].

A search was performed to find the global minima for the metal-containing cages **1** and **2**. First, the geometry of structure **2** was optimized at the GFN-FF level with OPTIM, followed by a search of confirmation space at the GFN-FF level with GMIN. A charge of +7 was applied to the structure and the calculations were performed in the gas-phase. The GMIN calculation was allowed to run for 5000 BH steps with a maximum perturbation step size of 0.5 Å and a maximum of 20 lowest energy minima (with an energy difference of at least 0.003 Eh) were saved. A similar method was applied to structure **1**, however a general atom-atom harmonic distance constraint potential was added for three Zn-MeCN bonds during the GMIN calculation to prevent the solvent molecules from dissociating. Additionally, a charge of +8 was applied to the structure. A constrained geometry optimization was performed at the GFN-FF level of the five lowest energy minima found for structure **1**. The GMIN algorithm did not locate any new lower energy structures for either structure compared to those found with OPTIM at the GFN-FF level (Supplementary Figure 93 a and b). The energy that was calculated using OPTIM as well as the lowest energy minima that were located using the GMIN algorithm (from lowest to highest) are shown in table Supplementary Table 3.

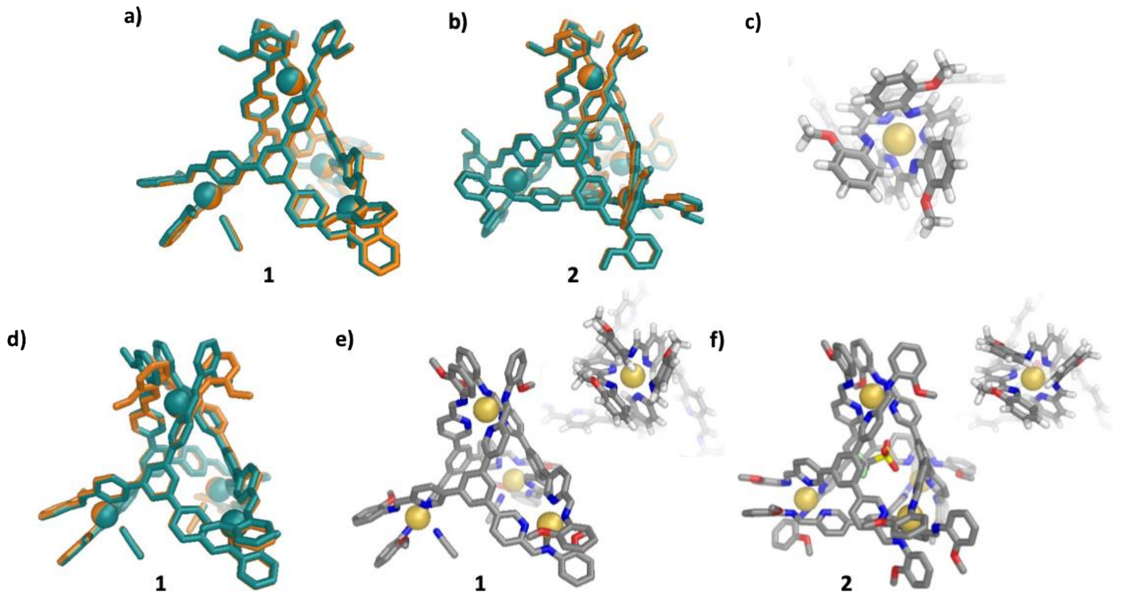

Supplementary Figure 93 | Comparison of the OPTIM (orange) and GMIN (teal) result for **a) 1** and **b) 2** at the GFN-FF level with identical geometries; **c)** C_3-symmetric octahedral complex present at the vertices of the structures shown in **a)** and **b)**; **d)** Comparison of the OPTIM (orange) and GMIN (teal) result for **1** at the GFN2-xTB level showing rearrangement of the imine moieties at one vertex; Final lowest energy geometry of **a) e** and **b) f** found at the GFN2-xTB level (+ implicit MeCN) and a top-view of the complex found at one or all of the vertices, respectively.

Supplementary Table 3. Lowest Energy Minima of 1 and 2 found with OPTIM and GMIN at both the GFN-FF and GFN2-xTB (+implicit MeCN) levels of theory. Computed energy values (in Hartree) for the investigated structures.

Minima 11-20 are omitted for structure **2** as they did not lead to any lower energy minima. The lowest energy minima found for each method are highlighted in teal.

		1				2	
		GFN-FF	GFN2-xTB (MeCN)			GFN-FF	GFN2-xTB (MeCN)
OPTIM:		-48.80130	-459.62692			-67.06593	*-610.18227*
GMIN:	1	-48.79914[a] *-48.80130*[b]	-459.62778		1	*-67.06594*	-610.16934
	2	-48.79914[a]	-459.62800		2	-67.06107	-610.16671
	3	-48.60354[a]	-459.62802		3	-67.06107	-610.16670
	4	-48.53779[a]	-459.62753		4	-67.05742	-610.16961

	5	-47.46356[a]	*-459.63026*	5	-67.05238	-610.17376
				6	-67.05238	-610.17530
				7	-66.99423	-610.13437
				8	-66.96258	-610.17076
				9	-66.96256	-610.16837
				10	-66.94902	-610.17527

[a] Energies resulting from the constrained GMIN run. [b] Energy resulting after unconstrained GFN-FF relaxation from the constrained GMIN run.

Following these results, further (unconstrained) geometry optimizations were performed on structure **1** and **2** at the GFN2-xTB level using the OPTIM program. Charges of +7 and +8 were applied, respectively, and implicit MeCN solvation was included. This local geometry optimization resulted in a rearrangement of the imine-moieties in the four metal complexes of structure **2** and thus the loss of *T*-symmetry from the input structure (Supplementary Figure 93f). A similar rearrangement for the analogous complex in structure **1** was not found at this stage (Supplementary Figure 93d, orange). Consecutive relaxations at the GFN2-xTB level (+ implicit MeCN solvation) were performed on each minimum found with the GMIN step discussed in the previous paragraph. This procedure led to the discovery of a lower minimum for structure **1** compared to the one found initially with OPTIM (Supplementary Figure 93d and e). No lower minimum was found for structure **2**. The final lowest energy structures for **1** and **2** now both exhibit the rearranged imine moieties at one and four vertices, respectively. This rearrangement could be explained by pi-pi interactions and free rotation of phenyl rings, leading to a decrease in symmetry of the overall structure. However, from experimental NMR results it was observed that structures **1** and **2** should be C_3 and *T*-symmetric in solution at room temperature. Therefore, it can be concluded that the geometries obtained at the GFN-FF level are likely closer to the real geometries in the experiments. Supplementary Table 3 shows all the lowest energy minima found for each method, with the lowest energy minima as calculated at the GFN2-xTB level highlighted in italics and in teal.

A similar search of the energy landscape was performed on the woven structures **5** and **6** to find their global minima. Structure **5** and **6** were first optimized at both the GFN-FF level and GFN2-xTB level using OPTIM, followed by a search of configuration space at

the GFN-FF level with GMIN. Each of the 20 lowest energy minima were relaxed at the GFN2-xTB level using the OPTIM program. The GFN2-xTB calculations with both OPTIM and GMIN included implicit DCM solvation. The new lowest energy minima for both structures are shown in Supplementary Figure 94 c and f, and the energy values found are shown in Supplementary Table 4.

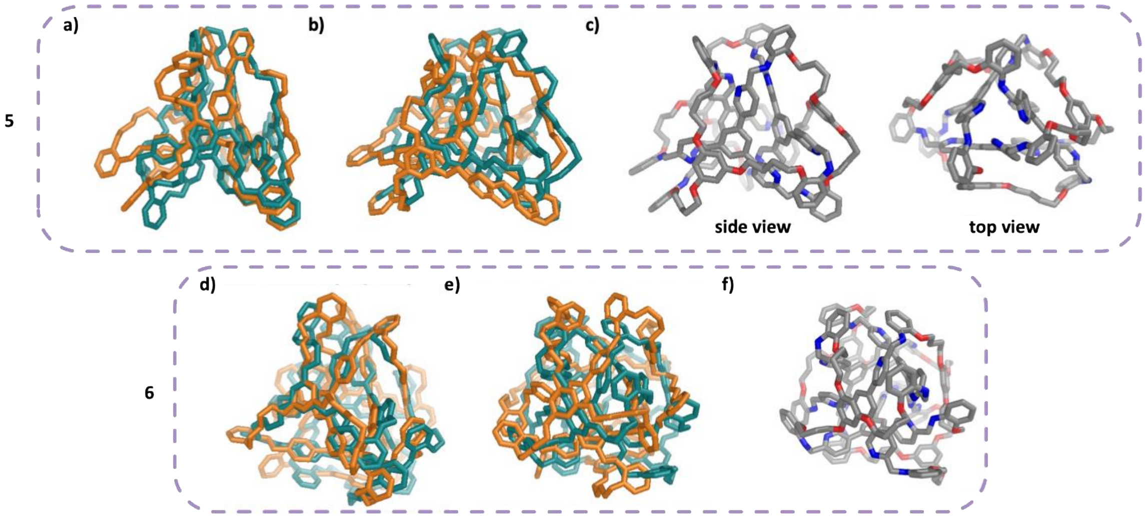

Supplementary Figure 94 | Comparison of the OPTIM (orange) and GMIN (teal) result for structure **5** at the **a)** GFN-FF level and the **b)** GFN2-xTB (+ implicit MeCN) level showing the new lowest energy minima; **c)** Lowest energy geometry of structure **5** found at the GFN2-xTB level (+ implicit MeCN), shown from two different angles; Comparison of the OPTIM (orange) and GMIN (teal) result for structure **6** at the **d)** GFN-FF level and the **e)** GFN2-xTB (+ implicit MeCN) level showing the new lowest energy minima; **f)** Lowest energy geometry of structure **6** found after the initial search of the energy landscape with OPTIM and GMIN. Hydrogens are omitted for clarity in each structure.

Supplementary Table 4. Lowest Energy Minima of 5 and 6 found with OPTIM and GMIN at both the GFN-FF and GFN2-xTB levels of theory. Computed energy values (in Hartree) for the investigated structures.

The GFN2-xTB calculations included implicit DCM solvation. Minima 15-20 are omitted for both structures as they did not lead to any lower energy structures. The lowest energy minima found for each method are highlighted in teal.

		5				6	
		GFN-FF	GFN2-xTB (DCM)			GFN-FF	GFN2-xTB (DCM)

**OPTIM:**		-76.78332	-603.51410			-102.41595	-804.66158
**GMIN:**	1	*-76.81213*	*-603.52282*		1	*-102.45037*	-804.68361
	2	-76.80950	-603.51959		2	-102.44684	-804.68491
	3	-76.80630	-603.51736		3	-102.44203	-804.70095
	4	-76.80346	-603.51637		4	-102.43862	-804.70111
	5	-76.80062	-603.52006		5	-102.43431	-804.69958
	6	-76.79631	-603.51308		6	-102.43156	-804.68416
	7	-76.79286	-603.50462		7	-102.42877	-804.69311
	8	-76.78988	-603.51205		8	-102.42742	-804.69818
	9	-76.78964	-603.50409		9	-102.42187	-804.69441
	10	-76.78380	-603.50925		10	-102.41869	-804.68900
	11	-76.78124	-603.50244		11	-102.41844	-804.69280
	12	-76.78063	-603.50580		12	-102.41601	-804.70188
	13	-76.77837	-603.50357		13	-102.41286	-804.69931
	14	-76.77801	-603.50515		14	-102.40989	*-804.70270*

Based on experimental NMR results, structure **5** was determined to exhibit C_{3h} symmetry in solution. However, the significant energy barrier associated with structural rearrangement renders direct computational predictions, starting from a tetrahedral input geometry, incapable of fully accounting for the experimental observations. Therefore, a more thorough search of configuration space was performed. Molecular dynamics (MD) calculations were run for structure **5** using the *xtb* program in order to create an ensemble of 1000 conformers (ID's 1-1000). The MD simulation was performed at the GFN-FF level in the NVT ensemble at 600 K starting from the GFN-FF optimized structure obtained with OPTIM. The following parameters were used: time steps 2.0 fs, total simulation time 50.0 ps and interval for trajectory printout 50.0 fs. BH global optimization calculations at the GFN-FF level were performed using GMIN on six evenly spaced-out conformations extracted from the ensemble, analogous to the previous GMIN calculations. A charge of zero was applied for each of these calculations. GFN2-xTB relaxations were performed on the 20 lowest energy minima that were found after each GMIN run using the OPTIM program. The results for the conformer with ID **1000** provided five new minima (Supplementary Table 5 and Supplementary Figure 95) that are each lower in energy than the one found previously for structure **5** (Supplementary Figure 94f). GMIN runs on the other conformers did not lead to any relevant minima

lower minima these five. The lowest energy minimum (Supplementary Figure 95f) does not resemble the starting tetrahedron. A similar approach was applied to two structures obtained from the MD conformer ensemble calculated for structure **6**, however this search did not lead to any new physically relevant lower energy minima.

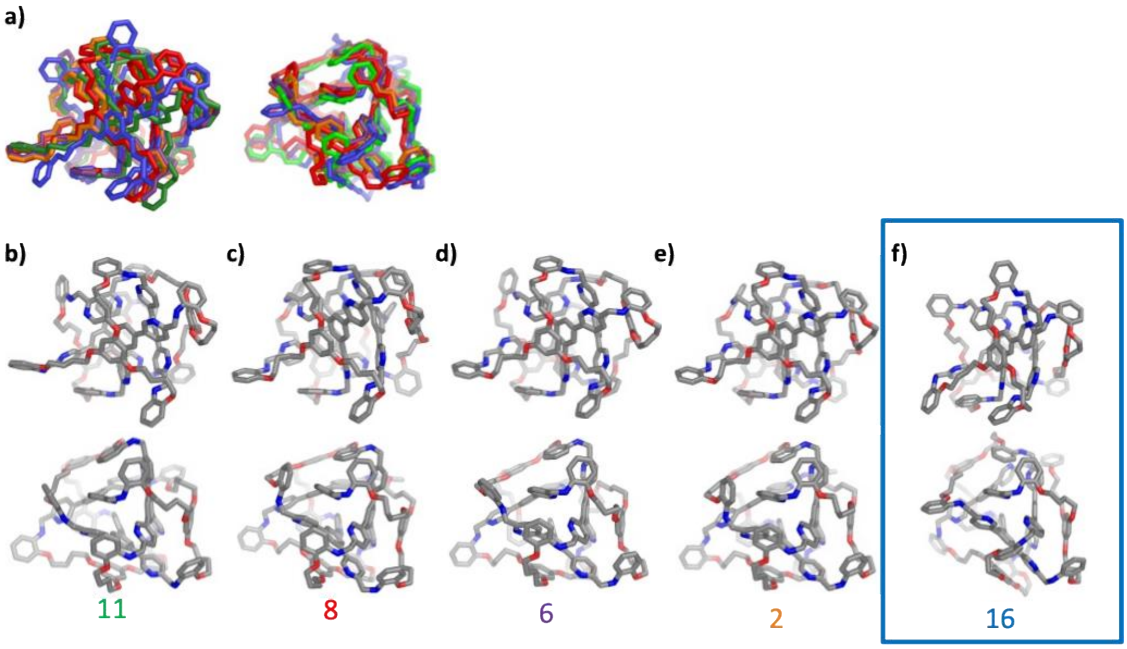

Supplementary Figure 95 | a) Comparison of five lowest energy minima found after GFN2-xTB relaxations of the GMIN results starting from the conformation of structure **5** with ID **1000**; **b)** Sixth-to-lowest (green); **c)** fourth-to-lowest (red); **d)** third-to-lowest (purple); **e)** second-to-lowest (orange) and **f)** lowest (blue) energy minima found for structure **5** at the GFN2-xTB (+ implicit DCM) level. Each structure is shown from two different angles. The ID number for the minima are shown below the structure. Hydrogens are omitted for clarity.

We note that basin-hopping is a stochastic approach, so it is possible that we have not yet located the true global minimum. However, we are confident that we have found good candidates in these searches.

Supplementary Table 5. Lowest Energy Minima of 5 found with GMIN at both the GFN-FF and GFN2-xTB levels of theory, starting from the conformer with ID 1000 found with MD. Computed energy values (in Hartree) for the investigated structures. The GFN2-xTB calculations were performed with implicit DCM solvation. The five lowest energy minima are highlighted in blue, orange, purple, red and green respectively.

	5						
		GFN-FF	GFN2-xTB (DCM)			GFN-FF	GFN2-xTB (DCM)
GMIN	1	-76.78318	-603.52234		11	-76.77345	-603.52566
	2	-76.78182	-603.52735		12	-76.76702	-603.51947
	3	-76.78140	-603.51976		13	-76.76699	-603.51913
	4	-76.78140	-603.51980		14	-76.76599	-603.51592
	5	-76.78140	-603.51980		15	-76.76419	-603.51684
	6	-76.78102	-603.52614		16	-76.76322	*-603.53271*
	7	-76.77851	-603.51568		17	-76.76271	-603.51539
	8	-76.77840	-603.52605		18	-76.76017	-603.51656
	9	-76.77578	-603.51480		19	-76.75181	-603.52040
	10	-76.77362	-603.51818		20	-76.74984	-603.52241

Further MD calculations were performed in order to assess the flexibility of the cages **2** and **4**, and gain insight in their ability to realize anions exchanges. The results can be found in Supplementary Figure 96. Here we see that cage **4** exhibits smaller H-H distances, consist with smaller aperture widths compared to cage **2**.

Procedure: The parametrization of both the atomistic models **2** and **4** was based on the CHARMM General Force Field (CGenFF)[28] framework. The partial charges of the organic ligands were estimated using the RESP approach[29] implemented on the R.E.D server,[30] based on quantum mechanical calculations performed using Gaussian16 software[31] with a (U)B3LYP functional[32] and a 6-31G(d) basis set. The bonded parameters of the organic components of the cages were taken from CGenFF, while the metal-coordination centres were parametrized using the nonbonded dummy atom model scheme.[33]

All the classical molecular dynamics (MD) simulations were carried out in explicit solvent (acetonitrile), incorporating 7 TfO^- anions to balance the +7 charge of the host-

guest complexes. We used the GROMACS-2021.7 software[34,35]. The simulations were performed in cubic boxes with 4.0 nm side. For each MD calculation, the system underwent a geometrical optimization, followed by equilibration phases. The equilibration consisted of 1 ns in the NVT ensemble at a temperature thermostat of 300 K and 1 ns in the NPT ensemble at a temperature thermostat of 300 K with a pressure barostat of 1 atm. Subsequently, a production run of 10 ns was carried out in the NPT ensemble at a temperature thermostat of 300 K and a pressure barostat of 1 atm. The sampling step chosen for the production run was 1 ps. As a thermostat we used v-rescale[36] for NVT simulations, while a Parrinello-Rahman barostat[37] was used for NPT simulations. The electrostatic interactions were treated using a Particle Mesh Ewald approach[38]. The cutoff of the real part of the electrostatic interaction was set to 1.2 nm. The cutoff of the van der Waals interaction was set to 1.2 nm. All bonds involving hydrogens were restrained by using the LINCS algorithm[39].

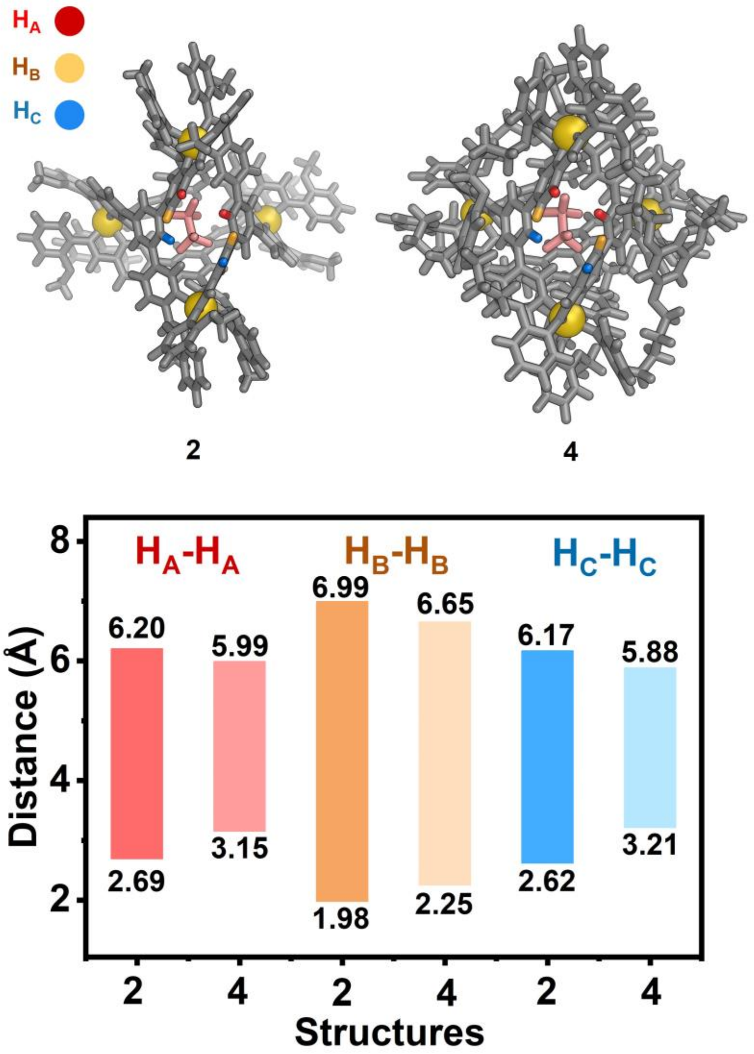

Supplementary Figure 96 | MD simulations were conducted, the apertures within the interior tetrahedral core of **2** and **4**. **4** exhibit a reduced proton-proton largest distance between adjacent faces compared to those in framework **2**, corresponding to a decrease in aperture width by about 0.3 Å.

8.2 Input files

Input files for OPTIM calculations

The odata files for the OPTIM calculations at the GFN-FF and GFN2-xTB level of structures A-D contained the following keywords:

```
BFGSMIN 1.0D-6
CONVERGE 0.1 1.0D-6
UPDATES 500 100 5 5
MAXERISE 1.0D-5 0.02D0
MAXBFGS 0.2 0.1
BFGSSTEPS 10000

RADIUS 2000.0
DUMPDATA
ENDNUMHESS

XTB structure.xyz runparams
```

structure.xyz contained the file with the input coordinates for each calculation, which in this case were the MM3-optimized geometries obtained through Scigress. *runparams* contains the option given to the *xtb* executable in the form of a string. The following strings were provided for the GFN-FF calculations:

1. ' --acc 0.01 --chrg 8 --gfnff '
2. ' --acc 0.01 --chrg 7 --gfnff '
5. ' --acc 0.01 --chrg 0 --gfnff '
6. ' --acc 0.01 --chrg 0 --gfnff '

The following strings were provided for the GFN2-xTB calculations:

1. ' --acc 0.01 --chrg 8 --alpb acetonitrile --gfn2 '
2. ' --acc 0.01 --chrg 7 --alpb acetonitrile --gfn2 '
5. ' --acc 0.01 --chrg 0 --alpb ch2cl2 --gfn2 '

6. ' --acc 0.01 --chrg 0 --alpb ch2cl2 --gfn2 '

Input files for GMIN calculations

The data files for the GMIN calculations at the GFN-FF level of structures A-D contained the following keywords:

```
MAXERISE 1.0D-5 0.02D0
MAXBFGS 0.2 0.1

DUMPINT 50
EDIFF 0.003
SAVE 20
MAXIT 1000 500000
STEPS 5000 1.0
FIXBOTH
ACCEPTRATIO 0.5D0

TEMPERATURE 0.02
STEP 0.5 0.0
SLOPPYCONV 0.0001
TIGHTCONV 1.0D-6
UPDATES 200
COLDFUSION -115.0D0

PERCOLATE 3.0
XTB structure.xyz runparams
```

“structure.xyz” contained the file with the input coordinates for each calculation, which in this case were the MM3-optimized geometries obtained through Scigress. “runparams” contains the options given to the *xtb* executable in the form of a string. The following strings were provided were:

1. ' --acc 0.01 --chrg 8 --gfnff '

2. ' --acc 0.01 --chrg 7 --gfnff '
5. ' --acc 0.01 --chrg 0 --gfnff '
6. ' --acc 0.01 --chrg 0 --gfnff '

A constrained GMIN calculation was applied for structure **1** to prevent dissociation of the MeCN ligands during the conformational search. This constraint was achieved by adding the following keywords to the data file:

```
DISTCON constraints
```

“constraints” refers to a file containing the following lines:

```
287 32   2.098 1.01D3
275 1    2.098 1.01D3
281 147 2.098 1.01D3
```

Input files for OPTIM calculations on the minima found by the GMIN calculation

The odata files for the OPTIM calculations at the GFN2-xTB level of the lowest energy minima found by GMIN for structures A-D contained the following keywords:

```
BFGSMIN 1.0D-6
CONVERGE 0.1 1.0D-6
UPDATES 500 100 5 5
MAXERISE 1.0D-5 0.02D0
MAXBFGS 0.2 0.1
BFGSSTEPS 10000

RADIUS 2000.0
DUMPDATA
ENDNUMHESS

XTB structure_i.xyz runparams
```

structure_i.xyz contained the file with the input coordinates for each calculation, which in this case were the 20 (or 5 for **1**) lowest local energy minima found during the GMIN calculation. runparams contains the options given to the *xtb* executable in the form of a string. The following strings were provided were:

1. ' --acc 0.01 --chrg 7 --alpb acetonitrile --gfn2 '
2. ' --acc 0.01 --chrg 8 --alpb acetonitrile --gfn2 '
5. ' --acc 0.01 --chrg 0 --alpb ch2cl2 --gfn2 '
6. ' --acc 0.01 --chrg 0 --alpb ch2cl2 --gfn2 '

9. References

1. Castilla, A. M., Ronson, T. K. & Nitschke, J. R. Sequence-Dependent Guest Release Triggered by Orthogonal Chemical Signals. *J. Am. Chem. Soc.* **138**, 2342–2351 (2016).
2. Chen, S.-G. *et al.* Highly Stable Chiral (A)$_6$ –B Supramolecular Copolymers: A Multivalency-Based Self-Assembly Process. *J. Am. Chem. Soc.* **133**, 11124–11127 (2011).
3. Allan, D. R. *et al.* A Novel Dual Air-Bearing Fixed-χ Diffractometer for Small-Molecule Single-Crystal X-ray Diffraction on Beamline I19 at Diamond Light Source. *Crystals 2017,* **7**, 336 (2017).
4. Evans, P. Scaling and assessment of data quality. in *Acta Crystallogr. D Struct. Biol.*, **62** 72–82 (International Union of Crystallography, 2006).
5. Winter, G. Xia2: An expert system for macromolecular crystallography data reduction. *J. Appl. Crystallogr.* **43**, 186–190 (2010).
6. Winter, G. *et al.* DIALS: Implementation and evaluation of a new integration package. *Acta Crystallogr. D Struct. Biol.* **74**, 85–97 (2018).
7. Farrugia, L. J. WinGX and ORTEP for Windows: An update. *J. Appl. Crystallogr.* **45**, 849–854 (2012).
8. Hübschle, C. B., Sheldrick, G. M. & Dittrich, B. *ShelXle*: a Qt graphical user interface for *SHELXL*. *J. Appl. Crystallogr.* **44**, 1281–1284 (2011).
9. Sheldrick, G. M. SHELXT - Integrated space-group and crystal-structure determination. *Acta Crystallogr. A* **71**, 3–8 (2015).

10. Sheldrick, G. M. Crystal structure refinement with SHELXL. *Acta Crystallogr. C Struct. Chem.* **71**, 3–8 (2015).
11. Bricogne, G. *et al. BUSTER. 2.11.2 Ed.; Global Phasing Ltd.: Cambridge, United Kingdom, 2011.*
12. Smart, O. S. & Womack, T. O. *Grade Web Server*. (Global Phasing Ltd., 2014).
13. Sluis, P. Van Der & Spek, A. L. BYPASS: an effective method for the refinement of crystal structures containing disordered solvent regions. *Acta Crystallogr. A* **46**, 194–201 (1990).
14. Spek, A. L. *PLATON: A Multipurpose Crystallographic Tool*. (Utrecht University: Utrecht, The Netherlands, 2008).
15. OPTIM: A program for geometry optimisation and pathway calculations. *http://www-wales.ch.cam.ac.uk/software.html*.
16. Wesołowski, P. A., Wales, D. J. & Pracht, P. Multilevel framework for analysis of protein folding involving disulfide bond formation. *J. Phys. Chem. B* **128**, 3145–3156 (2024).
17. Li, Z. & Scheraga, H. A. Monte Carlo-minimization approach to the multiple-minima problem in protein folding. *Proc. Natl. Acad. Sci. USA* **84**, 6611-6615 (1987).
18. Wales, D. J. & Doye, J. P. K. Global optimization by basin-hopping and the lowest energy structures of Lennard-Jones clusters containing up to 110 atoms. *J. Phys. Chem. A* **101**, 5111–5116 (1997).
19. GMIN: A program for basin-hopping global optimisation, basin-sampling, and parallel tempering. *http://www-wales.ch.cam.ac.uk/software.html*.
20. Miller, M. A. & Wales, D. J. Novel structural motifs in clusters of dipolar spheres: Knots, links, and coils. *J. Phys. Chem. B* **109**, 23109–23112 (2005).
21. Spicher, S. & Grimme, S. Robust atomistic modeling of materials, organometallic, and biochemical systems. *Angew. Chem. Int. Ed.* **59**, 15665–15673 (2020).
22. Bannwarth, C., Ehlert, S. & Grimme, S. GFN2-xTB—An accurate and broadly parametrized self-consistent tight-binding quantum chemical method with multipole electrostatics and density-dependent dispersion contributions. *J. Chem. Theory Comput.* **15**, 1652–1671 (2019).
23. Bursch, M., Neugebauer, H. & Grimme, S. Structure optimisation of large transition‐metal complexes with extended tight‐binding methods. *Angew. Chem.*

Int. Ed. **58**, 11078–11087 (2019).

24. Allinger, N. L., Yuh, Y. H. & Lii, J. H. Molecular mechanics. The MM3 force field for hydrocarbons. 1. *J. Am. Chem. Soc.* **111**, 8551–8566 (1989).
25. Lii, J. H. & Allinger, N. L. Molecular mechanics. The MM3 force field for hydrocarbons. 2. Vibrational frequencies and thermodynamics. *J. Am. Chem. Soc.* **111**, 8566–8575 (1989).
26. Lii, J. H. & Allinger, N. L. Molecular mechanics. The MM3 force field for hydrocarbons. 3. The van der Waals' potentials and crystal data for aliphatic and aromatic hydrocarbons. *J. Am. Chem. Soc.* **111**, 8576–8582 (1989).
27. Marchand, N., Lienard, P., Siehl, H.-U. & Izato, H. Applications of molecular simulation software SCIGRESS in industry and university. *FUJITSU Sci. Tech. J* **50** (2014).
28. Vanommeslaeghe, K. *et al.* CHARMM general force field: A force field for drug-like molecules compatible with the CHARMM all-atom additive biological force fields. *J. Comput. Chem.* **31**, 671–690 (2010).
29. Bayly, C. I., Cieplak, P., Cornell, W. & Kollman, P. A. A well-behaved electrostatic potential based method using charge restraints for deriving atomic charges: the RESP model. *J. Phys. Chem.* **97**, 10269–10280 (1993).
30. Vanquelef, E. *et al.* R.E.D. Server: a web service for deriving RESP and ESP charges and building force field libraries for new molecules and molecular fragments. *Nucleic Acids Res.* **39**, W511–W517 (2011).
31. Frisch, M. J. *et al.* Gaussian 16, Revision C.01. Preprint at (2016).
32. Becke, A. D. Density-functional thermochemistry. III. The role of exact exchange. *J. Chem. Phys.* **98**, 5648–5652 (1993).
33. Duarte, F. *et al.* Force field independent metal parameters using a nonbonded dummy model. *J. Phys. Chem. B* **118**, 4351–4362 (2014).
34. Hess, B., Kutzner, C., van der Spoel, D. & Lindahl, E. GROMACS 4: Algorithms for highly efficient, load-balanced, and scalable molecular simulation. *J. Chem. Theory Comput.* **4**, 435–447 (2008).
35. Abraham, M. J. *et al.* GROMACS: High performance molecular simulations through multi-level parallelism from laptops to supercomputers. *SoftwareX* **1–2**, 19–25 (2015).
36. Bussi, G., Donadio, D. & Parrinello, M. Canonical sampling through velocity

rescaling. *J. Chem. Phys.* **126**, (2007).

37. Parrinello, M. & Rahman, A. Polymorphic transitions in single crystals: A new molecular dynamics method. *J. Appl. Phys.* **52**, 7182–7190 (1981).
38. Essmann, U. *et al.* A smooth particle mesh Ewald method. *J. Chem. Phys.* **103**, 8577–8593 (1995).
39. Hess, B., Bekker, H., Berendsen, H. J. C. & Fraaije, J. G. E. M. LINCS: A linear constraint solver for molecular simulations. *J. Comput. Chem.* **18**, 1463–1472 (1997).
